# Comparative Lipidomic Study of Human Milk from Different Lactation Stages and Milk Formulas

**DOI:** 10.3390/nu12072165

**Published:** 2020-07-21

**Authors:** Weronika Hewelt-Belka, Dorota Garwolińska, Michał Młynarczyk, Agata Kot-Wasik

**Affiliations:** Department of Analytical Chemistry, Chemical Faculty, Gdańsk University of Technology, 80-233 Gdańsk, Poland; dorota.garwolinska@pg.edu.pl (D.G.); michal.mlynarczyk2000@gmail.com (M.M.); agawasik@pg.edu.pl (A.K.-W.)

**Keywords:** human milk, milk formula, human milk lipid composition, lipidomics, infant nutrition, lactation, human milk composition, infant feeding, foodomics, breastfeeding, infant formula

## Abstract

In this report, we present a detailed comparison of the lipid composition of human milk (HM) and formula milk (FM) targeting different lactation stages and infant age range. We studied HM samples collected from 26 Polish mothers from colostrum to 19 months of lactation, along with FM from seven brands available on the Polish market (infant formula, follow-on formula and growing-up formula). Lipid extracts were analysed using liquid chromatography coupled to high-resolution mass spectrometry (LC–Q-TOF–MS). We found that the lipid composition of FM deviates significantly from the HM lipid profile in terms of qualitative and quantitative differences. FM had contrasting lipid profiles mostly across brands and accordingly to the type of fat added but not specific to the target age range. The individual differences were dominant in HM; however, differences according to the lactation stage were also observed, especially between colostrum and HM collected in other lactation stages. Biologically and nutritionally important lipids, such as long-chain polyunsaturated fatty acids (LC-PUFAs) containing lipid species, sphingomyelines or ether analogues of glycerophosphoethanoloamines were detected in HM collected in all studied lactation stages. The observed differences concerned all the major HM lipid classes and highlight the importance of the detailed compositional studies of both HM and FM.

## 1. Introduction

Human milk (HM) is considered an optimal source of nutrients and bioactive compounds for new-borns [1]. Human milk contains 3–5% (*w*/*v*) fat that provides 40–50% of total energy and essential fatty acids for infants [2]. However, HM lipids have far more biological roles than just providing energy [3]. HM lipids are very diversified in chemical structure and concentration levels. The HM lipid fraction contains glycerolipids, such as triacylglycerol (TG), diacylglycerol (DG), monoacylglycerol (MG), glycerophospholipids (GP), sphingolipids (SP), sterols (ST) and free fatty acids. The highly abundant triacylglycerol (TG, 98% of total lipids), which has a specific fatty acid composition and structure, is a major source of energy and a core of the milk fat globule (MFG). Phospholipids (PLs) are low-abundant HM components (0.5–1% of total lipids) but not of less importance. Phospholipids, namely glycerophospholipids (GP), sphingolipids (SP), and gangliosides, form the milk fat globule membrane (MFGM) [4]; they are positively associated with neurological and cognitive development, immunological system modulation and the antimicrobial properties of human milk [5].

The World Health Organization (WHO) and United Nations Children’s Fund (UNICEF) recommends exclusive breastfeeding for six months after birth and continued breastfeeding along with appropriate complementary foods for up to two years of age or beyond, while human milk is a source of a wide range of different valuable components [6]. However, the Breastfeeding Report Card, published by the Centers for Disease Control and Prevention (CDC) in 2018 states that 57.6% of children in the United States were breastfed at 6 months of life and, at 12 months of life, that number decreased to 35.9% [7]. In Poland, 38% of children are breastfed at 6 months of life and only 17% at 12 months of life [8]. The decision to breastfeed or resign from breastfeeding is highly personal and influenced by historical, socioeconomic, cultural and individual factors [9]. When breastfeeding is not possible, formula milk (FM) is widely used as a human milk substitute. FMs are specialised products designed for the dietary management of infants.

Many studies have shown that human milk metabolite composition differs significantly from the composition of milk formula due to the dynamic nature of HM and unique chemical structure of HM lipids [10,11,12,13,14,15,16]. Milk is species-specific, and its composition is dynamic along with lactation in terms of nutritive value and bioactive compounds [17,18]. HM composition also differs worldwide [19], making HM uniquely suited for feeding infants and not easily mimicked by FM. Breastfed infants were reported to have better short- and long-term outcomes than formula-fed infants. These outcomes include cognitive development, reduced risk of infectious diarrhoea and acute otitis media for breastfed children and different growth patterns, nutritional status, and gut microbiota that may be associated with a higher risk of obesity, diabetes, and cardiovascular diseases in adulthood for artificially fed infants [20,21]. Moreover, HM and FM nutrition regimens affect neonatal metabolism differently [22,23,24].

Lipidomics is now widely used in the comprehensive analysis of the lipidomes of various biological samples and is a valuable tool to analyse human milk [12,25,26,27,28,29,30]. Although, only a few studies have investigated lipidomic differences between HM and FM [31]. Generally, previous comparative analyses between FM and HM have focused mainly on specific lipid classes, such as TGs [14,15] or phospholipids [16,32,33]. Sun et al. [14] evaluated the triacylglycerol composition in 180 commercial infant formulas on the Chinese market based on the fat source and compared them with mature HM samples. They observed many lipidomic differences, such as higher proportions of TAGs with short-chain/medium-chain saturated fatty acids in the formulas compared with those in HM. They also revealed that TGs in the formulas were significantly affected by fat sources. Similar results were obtained by Tu et al. [15] when studying the composition of DGs and TGs in HM and FM samples. However, in these studies, HM was obtained at only two points of lactation: the seventh and 42nd days. Additionally, the phospholipid (PL) composition between the FMs and HM differed significantly. Jiang et al. [16] compared the phospholipid fingerprint of HM in Chinese women with the FMs available on the Chinese market. The PL concentrations varied significantly across the samples of HM and infant formulas. Zhu et al. [32] analysed the phospholipid profiles of powdered infant formulas using the different age range targets. The results showed that the PL content varied among FMs concerning various formulations used in processing. Another study by Furse et al. [28] revealed that the lipid profiles of infant formulas differ according to the manufacturer, region and date sold. In this study, the authors analysed FM samples from major brands in the UK, the Netherlands and South Africa.

Besides these studies, data concerning lipid composition differences between HM and FM, especially regarding advanced lactation (HM after 1 year postpartum) and corresponding growing-up formula, are remarkably scarce. Filling this knowledge gap is necessary to further determine the impact of these components on infant health, growth, and development, as well as to provide suitable nutrition for all infants.

In this study, we performed comparative LC–MS-based lipidomic analysis of the HM and FM samples to evaluate the differences between HM at different lactation stages, including HM samples at advanced lactation (>12 months postpartum) and the corresponding FM. The lipid fingerprints were compared and analysed using statistical and/or chemometric methods in terms of differences in the lipid composition: (i) within the HM samples collected in various stages of lactation; (ii) within the FM samples with various age range targets and fat sources; (iii) between the HM and FM samples.

To our best knowledge, this study is the first report to comprehensively describe differences in HM and FM lipidomes regarding the lactation stage, including longitudinal changes in HM samples collected after 1 year postpartum.

## 2. Materials and Methods

### 2.1. Chemicals

LC–MS-grade methanol, 2-propanol, and HPLC-grade chloroform were purchased from Merck (Darmstadt, Germany). Chemicals—ammonium formate (99.9% purity), formic acid and ammonium hydroxide solution (28%)—were purchased from Sigma-Aldrich (St. Louis, MO, USA). All aqueous solutions were prepared with ultra-pure water produced by an HLP5 system (Hydrolab, Wislina, Poland). The lipid standards 1-pentadecanoyl-2-oleoyl(d7)-sn-glycero-3-phosphoethanolamine (15:0-18:1-d7-PE) and 1,3-dipentadecanoyl-2-oleyol(d7)-glycerol (15:0-18:1-d7-15:0 TG) were purchased from Sigma-Aldrich (St. Louis, MO, USA).

### 2.2. Samples

Human milk samples were donated by 26 healthy women living in Pomeranian Voivodship, Poland, who had delivered healthy full-term neonates and had met the criteria of inclusion to the study (Inclusion criteria for the study are listed in Supplementary Materials). The samples were collected in several lactation periods: colostrum samples (*n* = 11); samples collected between 0 and 6 months (*n* = 10); samples collected between 6 and 12 months (*n* = 8); samples collected after 12 months (*n* = 16). Colostrum samples were obtained by a qualified midwife at the Obstetric Clinic, University Clinical Centre of the Medical University of Gdańsk. All volunteering mothers were given oral and written instructions for the standardised collection of milk samples. Written informed consent was obtained from each participant. The milk samples were obtained by the full expression of one breast using an electronic breast pump in the morning and/or in the evening. After collection, 10 mL of HM was transferred to the polypropylene laboratory tube and kept frozen at −20 °C until delivery to the laboratory. Samples were stored at −80 °C until analysis for a maximum of three months.

The formula milk samples were collected from the local pharmacy in Gdańsk, Poland. FM starting formula (age range target 0–6 months of life), follow-on formula (age range target 6–12 months of life), and the growing-up formula milk (age range target >12 months of life, available only for five brands of FM) of seven brands were included in the study. The detailed characteristics of all analysed samples are presented in Table S1 in the Supplementary Materials Section.

Research ethics approval was obtained from the Human Research Ethics Committee of the Medical University of Gdańsk, Poland (decision no. NKBBN/389/2019, date of approval: 8th of July 2019).

### 2.3. Sample Preparation

Sample preparation was performed using a previously developed extraction method based on the LLE and SPE techniques [30]. The HM and FM samples were extracted in duplicate. FM powders were weighed accurately and dissolved in deionised water before extraction, according to the manufacturer’s instructions included on the label.

#### 2.3.1. LLE Extraction

A total of 225 μL of the milk sample was transferred to a borosilicate-glass tube with a PTFE cap, followed by the addition of 950 μL of a chloroform/methanol mixture (1/2, *v/v*) and vigorous mixing for 10 s. After that, 310 μL chloroform and 310 μL deionised water were added. Next, the samples were mixed for another 30 s and centrifuged for 10 min at 4300 rpm to separate the aqueous and organic phases. The upper phase was discarded and the lower phase was transferred to a new borosilicate glass tube by a Pasteur pipette. Next, 20 μL of the obtained extract was diluted by adding 980 μL methanol to further dissolve the SPE extract.

#### 2.3.2. SPE-Based Phospholipid Extraction

To a 1.5-mL polypropylene tube, 100 μL milk sample, 890 μL 1% formic acid in methanol and 10 μL internal standard (1) 15:0-18:1-d7-PE were added. The contents were mixed for 30 s at 2000 rpm, followed by centrifugation for 10 min at 10,000 rpm. After that, 900 μL of the supernatant was loaded onto a HybridSPE-Phospholipid (bed weight, 20 mg) cartridge (Supelco, Sigma Aldrich, St. Louis, MO, USA), washed in sequence with 2 × 1 mL methanol and 2 × 1 mL 2-propanol. Finally, the phospholipids were eluted with 2 mL 5% ammonia in methanol. The extract was evaporated under a nitrogen stream at 35 °C. Next, 90 μL of the diluted LLE extract was used to dissolve the dried phospholipid extract. Additionally, 10 μL internal standard (2) 15:0-18:1-d7-15:0 TG was added to the final extract, which was then transferred to a 1.5-mL chromatographic vial and subsequently analysed by LC–Q-TOF–MS.

### 2.4. Lipid Fingerprinting by RPLC–Q-TOF–MS

Lipid analysis was conducted using an LC–Q-TOF–MS system—an Agilent 1290 LC system equipped with a binary pump, an online degasser, an autosampler and a thermostated column compartment coupled to a 6540 Q-TOF–MS with a dual electrospray ionisation (ESI) source (Agilent Technologies, Santa Clara, CA, USA). Lipid extracts were injected into a reversed-phase column Poroshell 120 EC-C8, 2.1 × 150 mm, 1.9 μm particle size (Agilent InfinityLab, Agilent Technologies, Santa Clara, CA, USA) with a 0.2-μm in-line filter. The column was maintained at 60 °C. The mobile phase comprised component A (5 mM ammonium formate in water/methanol (20/80, *v/v*)) and component B (2-propanol). The mobile phase was pumped with a flow rate of 0.3 mL/min. The gradient elution program was initiated with 20% component B, which was ramped to 40% from 0 to 20 min, then from 40% to 60% from 20 to 40 min and finally from 60% to 100% from 40 to 45 min. The column was then equilibrated with the starting conditions for 10 min. The total run time was 55 min, and the injection volume was set to 0.5 μL. The data were collected in the positive ion mode using the SCAN acquisition mode in a range from 100 to 1700 *m/z* in the high-resolution mode (4 GHz). MS analysis was carried out using the following parameters: capillary voltage, 3500 V; fragmentation voltage, 120 V; nebulising gas, 35 psig; drying gas temperature, 300 °C. MS/MS analysis was performed using identical chromatographic and ion source conditions. The collision energy was set to the following values: 35 V and 80 V. The two most abundant peaks were selected for fragmentation and excluded for the next 0.3 min. The MS/MS spectra were acquired in the *m*/*z* range of 50–1700. Lipid extracts were injected randomly using one Quality Control (QC) sample (pooled milk samples prepared identically to the real samples) injected every five real samples for the LC–MS stability control. The LC–MS batch started with the extraction blank and the five subsequent QC samples to equilibrate the chromatographic column. The lipid extracts were kept in the autosampler at 10 °C during the batch run.

### 2.5. Lipid Identification

Lipid identification was carried out using the two-step procedure: (1) a custom HM database automated search based on an accurately measured *m*/*z* value (Δ5 ppm tolerance) and (2) manual interpretation of the obtained MS/MS spectra of milk samples. Identification resulted in the determination of the lipid class, number of carbon atoms, and number of unsaturated bonds in fatty acid residues, as well as the presence of ether bonds instead of ester bonds in the lipid structure (e.g., TG 58:8 means triacylglycerol molecules with 58 carbon atoms and 8 double bonds in fatty acyl substituents; TG-O means ether analogue of triacylglycerol; SMd18:1/16:0 means sphingomyelin molecules with C18:1 sphingoid base backbone and C16:0 fatty acyl substituent). The fatty acid composition was evaluated based on MS/MS spectra interpretation. Lipid species with ether-linked substituents were not differentiated regarding ether and vinyl ether bonds in position sn-1. The position of fatty acyl substituents and the position of double bonds were not evaluated. The fragmentation lipid patterns for TGs, DGs, PEs, PCs and SMs were previously published [34]. The fragmentation pattern for PIs, PSs and ether analogues of TGs and PEs are presented in Figure S1 in the Supplementary Materials. The diagnostic ions for the lipid class confirmation were as follows: *m*/*z* 184.0726 for confirmation of the SM and PC identity; neutral loss of 141.02 Da for the confirmation of the PE identity; neutral loss of 185.01 Da for the confirmation of PS identity; *m*/*z* 264.27 for confirmation of the C18 sphingoid base backbone.

### 2.6. Data Treatment

The Molecular Feature Extraction (MFE) algorithm, implemented in the Agilent MassHunter Workstation Profinder 10.0 (Agilent Technologies, Santa Clara, CA, USA), was used to extract the total molecular features (MFs) from the raw LC–MS data using the following parameters: ion threshold, >1000 counts; ion type, H^+^; isotope model, common organic (no halogens); charge state range, 1–2; MFE score, ≥70. Next, the peak areas of the identified lipids (a list of these lipids is presented in Supplementary Materials, Table S2) were obtained using the Batch Targeted Feature Extraction algorithm with the following parameters: positive ions, charge carriers—H^+^, Na^+^, NH_4_^+^; match tolerance, 15 ppm; retention time, 0.3 min; Gaussian smoothing before extracted ion chromatogram extraction (EIC) filtering on peak height, 1000 counts. In both data pre-treatment approaches, the .cef files were exported and imported to Mass Profiler Professional 15.1 software (Agilent Technologies, Santa Clara, CA, USA) for data alignment and filtration. Missing values were exported as missing. The alignment parameters were set as follows: alignment slope = 0.0%; alignment intercept = 0.4 min; mass tolerance slope = 20.0 ppm; intercept = 2.0 mDa. Filtration was based on frequency (the MFs remained in the dataset if they were present in 80% of the samples in at least one specified group) and the QC %RSD (MFs remained if %RSD <20% in all the QC samples). The MFs that were present in the extraction blank, with the average peak volume higher than 10% of the average peak volume in the real samples, were removed. The statistical analyses and fold change calculation (ANOVA unequal variance, Mann–Whitney unpaired test) were conducted using Mass Profiler Professional 15.1 software (Agilent Technologies, Santa Clara, CA, USA) The parameters in the statistical tests were as follows: *p* ≤ 0.01; Multiple Testing Correction: Benjamini–Hochberg; *p*-value computation—asymptotic; missing values were excluded from the fold change and *p*-value calculations (statistical tests conducted only on detectable values); corrected *p*-value cut-off, 0.01; Post-hoc test: Tukey HSD(for ANOVA). Statistical tests and fold change calculations were conducted using the average peak area of samples within defined group of samples. The Metaboanalyst 4.0 web tool [35] was used for chemometric analysis (PCA, heatmap). The data for the analysis in Metaboanalyst 4.0 were prepared in Microsoft Excel 2016 software (Microsoft Corporation, Redmond, WA, USA). The data were log-transformed and auto scaled before PCA analysis. For heatmap analysis, the data were log-transformed and the Euclidean distance algorithm and Ward clustering algorithm were used. The missing values were treated as a very small value for PCA analysis, and as a zero value for a heatmap analysis. PCA analysis was also used to evaluate the drift in the MS signal abundance and MS stability through the analytical batch. The percentage relative amount of lipids within the specified lipid class was calculated in Microsoft Excel 2016 software (Microsoft Corporation, Redmond, WA, USA) by dividing the lipid species peak area by the sum of the peak area of all lipid species detected within the class.

## 3. Results

The analytical procedure adapted from our previous studies [30] allowed for obtaining extended lipidome coverage of the studied milk samples. The lipid extracts were analysed using the RPLC–Q-TOF–MS technique. The exemplary Total Ion Chromatograms (TICs) of the lipid extracts of the HM and FM samples, along with the identity of the most abundant lipid are presented in Figure S2 in the Supplementary Materials Section. The list of the identified lipids, along with the calculated percentage relative abundance within a specified lipid class is available in Supplementary Materials, Tables S3–S7. We compare the lipid compositions in the studied samples concerning the following: (i) percentage relative abundance of lipid species within a specific class of lipids (calculated by the division of a peak area of a lipid species by the sum of the peak areas of all lipids within the class); (ii) amount of each lipid species between a studied group of samples (the fold change value representing the average peak area response of lipid species in one group of samples over another, *p* < 0.01)

### 3.1. HM Lipidome Composition

**Figure 1:**
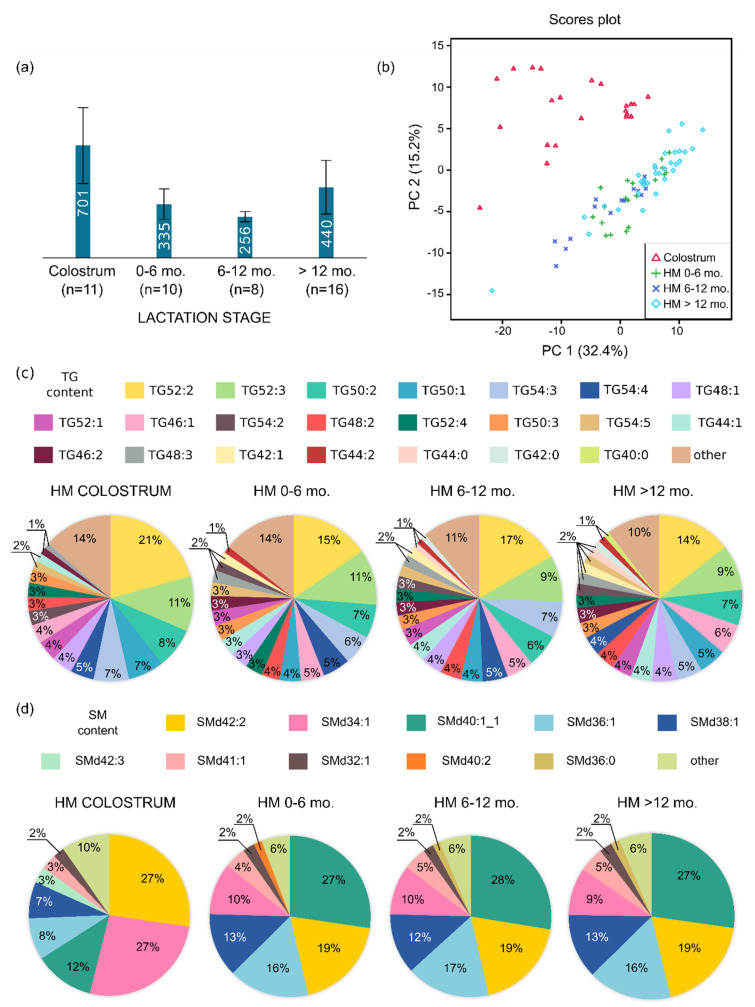
(**a**) Average number of MFs detected in HM samples collected in different lactation stages. (**b**) PCA score plot of the lipid fingerprints of HM samples: colostrum (red triangles); HM 0–6 months (green plus sign); HM 6–12 months (dark blue cross); HM >12 months (light blue diamonds). (**c**) Average percentage relative amount of lipid species in HM samples within the TG class. (**d**) Average percentage relative amount of lipid species in HM samples within the SM class. MF, molecular feature; SM, sphingomyelin; TG, triacylglycerol.

First, we studied the detailed composition of human milk according to the lactation stage. We analysed 90 lipid extracts of 45 samples of human milk collected from 26 women. The highest number of molecular features (MFs) was detected in the colostrum samples (average, 701), as shown in Figure 1a, and in the samples collected after 1 year postpartum (average, 440), as shown in Figure 1a.

The lipidome coverage of both colostrum and mature (HM) comprised lipid species belonging to the classes of triacylglycerols (TGs), diacylglycerols (DGs), monoacylglycerols (MGs), sphingomyelines (SMs), glycerophosphoethanoloamines (PEs), glycerophosphocholines (PCs), glycerophosphoinositoles (PIs) and glycerophosphoserines (PSs). We also detected ether analogues of PE, PC, and TG containing one ether-linked fatty acid in its structure apart from the acyl-linked FA. The percentage relative amount of many lipid species within all detected lipid classes was diversified between individual mothers and lactation stage, with the highest difference between colostrum and other lactation stages HM, as shown in Figure 1 and Supplementary Materials, Tables S4–S7. For example, the most abundant SMs in the colostrum samples were SMd42:2 (21–34%), SMd34:1 (19–36%) and SMd40:1 (9–15%), while in further lactation stage samples, they were SM40:1 (24–31%), SM42:2 (15–24%) and SMd36:1 (12–20%). The detailed characteristics of the lipid percentage relative amount of the individual mothers with a specified lactation stage are presented in Tables S4–S7 in the Supplementary Materials Section.

PCA was used to visualise the differences in the lipid pattern among the collected HM samples. The results of the unsupervised analysis are shown in Figure 1b. The first principal component (PC1) with PC2 describes 47.6% of the variation contained in the data responsible for the grouping of HM lipid fingerprints into two distinct groups, with colostrum samples separated from the samples collected in the further stages of lactation, which are positioned close to each other. This result shows that the lipid composition of colostrum samples is distinct from that of mature HM. The dispersion of the lipid fingerprints within particular groups can also be observed on the PCA score plot, as shown in Figure 1b, and indicates a large variance in lipid composition among the collected milk samples from individual mothers.

To further study the difference between the colostrum samples and mature milk samples, we performed statistical tests using the dataset containing only the identified lipids. Fifty-five percent of the lipid compounds included in the test (*n* = 215) were statistically significantly changed (*p* < 0.01) with a fold change (fc) ≥2 among colostrum and mature HM samples, with the peak area of 76 lipid species higher in mature milk and that of 40 lipid species higher in colostrum. The detailed list of the lipid components that were statistically different between colostrum and mature milk is shown in Tables S8 and S9 in the Supplementary Materials Section, while a short list can be seen in Table 1. The observed differences correspond to all of the major and minor lipid classes. Interestingly, we did not observe one trend for specific classes of lipids (i.e., an increase or decrease in all lipid species within one lipid class according to the lactation stage (colostrum vs. mature HM)).

Generally, the content of statistically different TG species (measured by the peak area) was higher in mature HM (mainly medium-chain triacylglycerols (MCTGs) and long-chain triacylglycerols (LCTGs) with a low level (1–3 double bonds) of unsaturation (HM 0–6 months and HM >12 months)) than in colostrum. However, the content of some TGs was significantly higher in colostrum samples than in mature HM, corresponding to TGs containing long-chain polyunsaturated fatty acids (LC-PUFAs) (e.g., TG 58:6, which contains in its structure 22:4, 18:1 and 18:1 fatty acyls (fc = 2.4) or TG 58:5, which contains a 22:4-18:0-18:1 fatty acyl composition (fc = 3.5)). Interestingly, the value of a fold change of many TG species was lower between colostrum and HM 6-12 months than the value of a fold change between colostrum and HM 0-6 months and HM >12 months (e.g., TG46:1, TG46:2, and TG46:3).

The colostrum samples also had a higher content of TGs containing fatty acyl substituents with more than 20 carbon atoms, such as TG 62:3 and TG 62:4, than mature milk (average fc >20). This also concerned alkyldiacylglycerols (captioned here as TG-O)—TG-O-52:2 (*p* < 0.01, fc = 3.5), TG-O-52:1 (*p* < 0.01, fc = 3.2) and TG-O 50:1 (*p* < 0.01, fc = 4.5)—which were all upregulated in colostrum samples.

Colostrum also contained statistically significantly more ether glycerophospholipids, such as PE-O 36:5 (fc = 2.6) and PE-O 38:5 (fc = 2.0), which contain LC-PUFAs eicosatetraenoic acid (C20:4) in their structure, PC-O 34:1 (fc = 1.8) and more PC34:0 (fc = 2.2), PC32:0 (fc = 2.8) and PC30:0 (fc = 2.7) containing saturated fatty acids compared with mature HM.

**Figure 2:**
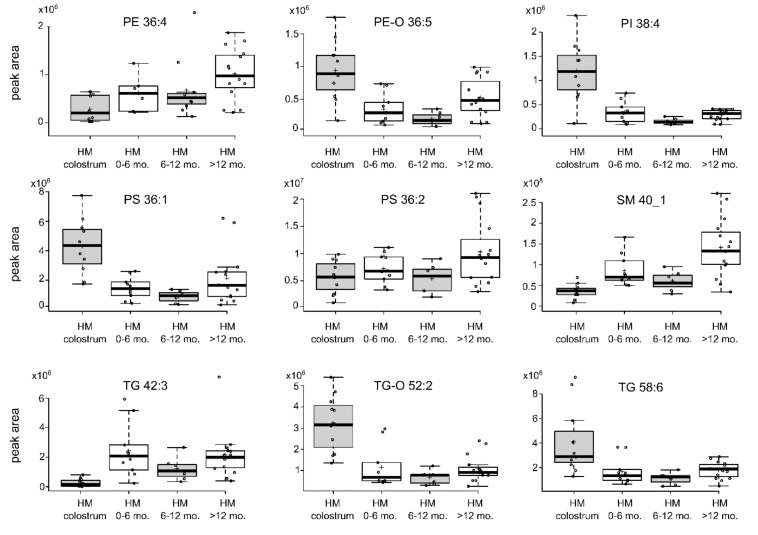
Comparison of the lipid peak area of samples collected in four lactation stages. The centre lines show the medians. The box limits indicate the 25th and 75th percentiles, as determined by R software. The whiskers extend 1.5-times the interquartile range from the 25th and 75th percentiles. The outliers are represented by dots; crosses represent sample means; data points are plotted as open circles. PE, glycerophosphoethanoloamine; PE-O, ether analogue of glycerophoshoethanoloamine; PS, glycerophosphoserine, PI, glycerophosphoinositol; SM, sphingomyelin; TG, triacylglycerol; TG-O, ether analogue of triacyglycerol.

We observed an increased content of PI38:4 (fc = 4.0) and PS36:1 (fc= 3.1) in colostrum compared with mature milk and some species of SM (SMd34:1, fc = 2.1; SMd34:21, fc = 2.6). However, the content of many other lipid species of these classes was higher in mature milk. Further exploration of HM lipid composition dynamics by visualising the variance in HM specific lipids, according to the four lactation stages on whiskers and box plots, showed that the lipid composition pattern is even more ambiguous, as shown in Figure 2. For example, the content of some lipid species was the highest in the colostrum samples, with a decrease in the period of 1 year of lactation and an increase after one year postpartum (e.g., PE-O36:5).

### 3.2. Formula Milk Lipidome

Next, we investigated the lipid composition of the milk formulas available on the Polish market. We studied infant formulas from seven brands, including starting formulas (0–6 months), follow-on formulas (6–12 months), and growing-up formulas (>12 months, available only for five brands). The lipid fraction of infant formulas was based on various sources of fat, including caprine whole milk, vegetable oils, fish oil, and microorganism lipids (e.g., oils from *Crypthecodinium cohnii* or *Mortierella alpine*). It was not always possible to determine whether FM contained isolated DHA or ARA from oils or contained them in the bound form in other lipid molecules, such as TGs. One of the formulas (FM.4.1.MFGM) was claimed to be enriched in milk fat globule membranes. The detailed characteristics of milk formulas and contained fat sources are presented in Table 2.

**Figure 3:**
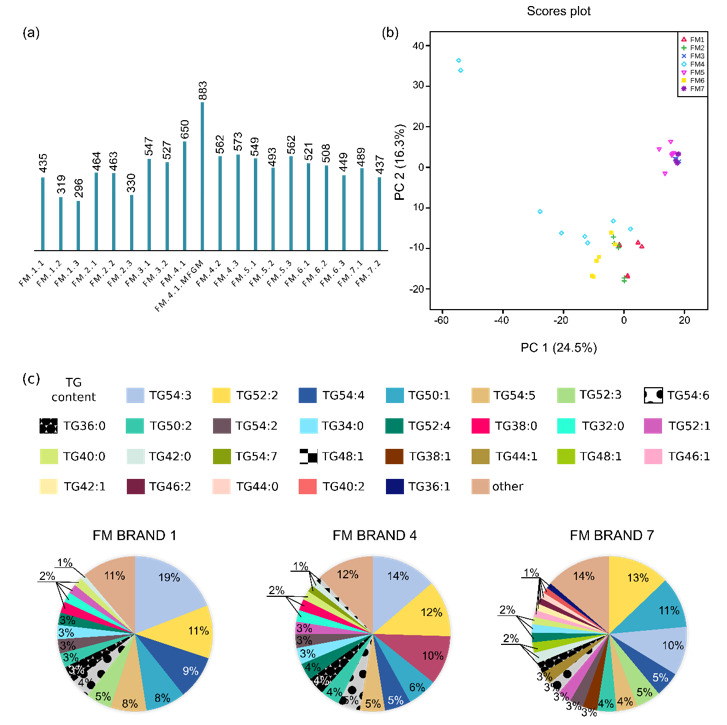
(**a**) Average number of MFs detected in the studied FM samples. FM1.1 stands for formula milk of brand one with an age range target of 0–6 months; FM1.2 stands for formula milk of brand one with an age range target of 6–12 months; FM1.3 stands for formula milk of brand one with an age range target <12 months. (**b**) The score plot of the lipid fingerprints of FM samples: brand one (red triangles), brand two (green plus sign), brand three (dark blue cross), brand four (light blue rhombi), brand five (reversed pink triangles), brand six (yellow rectangles), brand seven (purple star). (**c**) Average percentage relative amount of lipid species in all FM samples of brands one, four and seven. MF, molecular feature; TG, triacylglycerol.

The number of MFs detected in the lipid extracts varied between the analysed FM samples, as shown in Figure 3a, with a significantly higher number of compounds detected in the FM of brand four enriched in bovine MFGM (883) among other FMs. A trend with a decreasing number of MFs in FMs dedicated for older children can also be observed among all studied brands (i.e., the highest number of MFs was detected for starting formula and the smallest for growing-up formula).

The percentage relative amount of lipid species was diversified among various FM samples according to the brand and fat used as a human fat substituent, especially the soy lecithin or the caprine whole milk, as shown in Figure 3c and Table S7 in the Supplementary Materials section. Generally, the percentage relative amount of lipid species within specific classes does not change among the FMs of one brand dedicated to different age range targets.

To further explore the lipid patterns between FM samples of various brands and age targets, we performed PCA. PCA showed the most prominent distinction of lipid fingerprints based on the fat source as two denser areas could be seen in the PCA score plot presented in Figure 3b—one for FM1, FM2, FM4 and FM 6, which are milk formulas enriched with soy lecithin, and the other for FM3, FM5, and FM7, which are based on caprine whole milk and are not supplemented with soy lecithin. Therefore, the presence of caprine milk or soy lecithin is the main distinction among milk formula samples, based on lipid composition.

Generally, we did not observe grouping of the lipid fingerprints based on the age range target; however, the lipid fingerprints of the growing-up formulas of brands one, two and six (FM 1.3, FM 2.3 and FM 6.3, respectively; bottom corner of the PCA score) form a subgroup within the group of soy-lecithin-based FM, likely associated with the absence of coconut oil compared with the starting and follow-on formulas from the same brand. The lipid fingerprints of one of the studied FMs (FM4.1.MFGM; upper left corner of the PCA score) were positioned on the PCA score far from two groups of FM samples and correspond to FM enriched in milk fat globule membranes. Some of the statistically significant differences (*p* < 0.01) between the FMs classified into two groups (soy-lecithin supplemented FM and caprine milk FM), as indicated by PCA, are presented in Table 3 and the complete list is presented in Table S10 in the Supplementary Materials Section.

**Figure 4:**
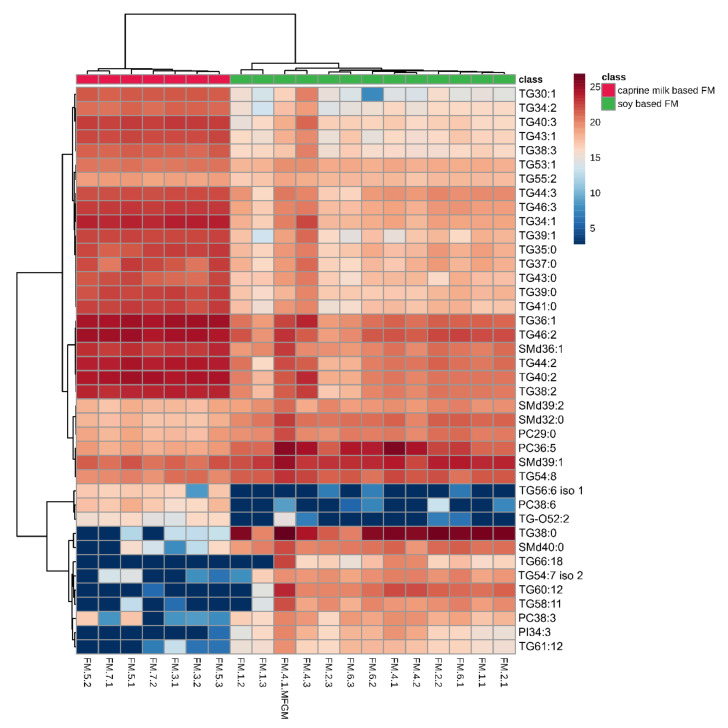
Clustering shown as a heatmap (distance measured using the Euclidean algorithm, and the clustering algorithm using Ward’s method. Top 40 lipid species differentiating HM and FM samples according to Mann–Whitney test unpaired.

Detailed insight into the variance in the lipid composition among the studied FM samples with respect to different brands and specific age targets was obtained by heatmaps presented in Figure 4.

The differences were observed in the content (measured by peak area fold change) of many medium-chain triacylglycerols (MCTGs) and long-chain TGs (LCTGs). FM with caprine whole milk (FM of brands three, five and seven) contained a significantly (*p* < 0.01) higher content of many lipid species belonging to the MCTs and LCTs with a low unsaturation level (total number of unsaturated bonds in FA substituents, 0–3) and with an odd total number of carbon atoms in FAs compared with FMs not supplemented with caprine milk (e.g., TG34:1, fc = 31.7). However, soy lecithin-supplemented FMs contained a higher level of some LC-PUFA TGs than caprine-based FMs (e.g., TG54:8, fc = 3.1).

The FM samples also differed in the content of many PC and PE species, with an especially high difference for species containing LCFA with the total number of double bonds = 4–5 in caprine whole milk-based FM compared with soy lecithin-supplemented FMs. Additionally, FMs containing soy lecithin fat (FMs of brands one, two, four and six) were richer in phospholipids than FMs not containing this ingredient.

For SM species, no specific trend of differences was observed, the content of some SM molecules was higher and others were lower in the FM caprine whole milk-based samples than in soy lecithin-supplemented FM.

The observed results show that the lipid composition is generally brand-specific and depends strongly on the used fat source.

### 3.3. Comparative Study of HM and FM Samples

Next, we compared the lipid composition of the HM and FM samples to explore the differences between them. For the statistical testing of differences at various lactation stages between HM and FM, all FM samples dedicated to the particular age target range were considered as one group without differentiation based on the fat source. We considered colostrum and HM samples collected in the period of 0–6 months separately to evaluate in detail the differences between HM samples obtained in this lactation stage and starting FM, because infants who cannot be breastfed directly after birth with colostrum are fed with FM dedicated for infants in age range 0–6 months.

The short-list of lipids significantly different (*p* < 0.01) between HM and FM samples is presented in Table 4—the detailed list with all statistically significantly different lipids is shown in Table S11 in the Supplementary Materials Section. The highest number of statistically significant different lipids was observed between colostrum/HM 0-6 month samples and starting formula samples—68% and 59% of the total lipids, respectively—and samples of HM >12 months and growing-up FM, 53% of the total lipids.

**Figure 5:**
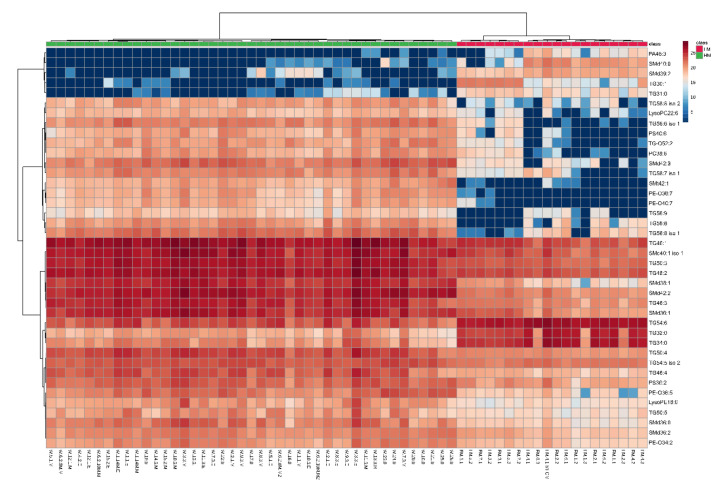
Clustering shown as a heatmap (distance measured using the Euclidean algorithm, and the clustering algorithm using Ward’s method). Top 40 lipid species differentiating HM and FM samples according to Mann–Whitney test unpaired.

To visualise the large diversity in the lipid composition in HM and FM samples, we used the heatmap presented in Figure 5. Clear clustering of HM and FM samples, based on the 40 statistically significant features, was observed. The detailed heatmaps performed for TGs, GPs and SMs can be found in the Supplementary Materials Section in Figures S3–S6.

The results clearly showed the HM and FM lipid composition varied significantly within all the classes of lipids. The HM and FM samples varied in the pattern and percentage relative abundance of the most abundant lipids within lipid classes, corresponding to all major HM lipid classes, as shown in Tables S3–S6 in the Supplementary Materials Section. For example, the most abundant TG in HM was TG52:2 with a FA composition 18:1-16:0-18:1, while in FM samples, the most abundant TG had different fatty acid compositions than those in HM: TG 54:3 in soy-supplemented FMs (18:1-18:1-18:1) and TG50:1 in caprine whole-milk-based FMs (18:1-16:0-16:0), as shown in Table S7 in the Supplementary Materials Section.

Statistically significant (*p* < 0.01) differences included MCTGs whose content was higher in formula milk than in HM, especially saturated TGs with the number of carbon atoms in fatty acid substituents lower than 40. Additionally, HM samples contained a higher content of TGs containing LC-PUFAs, such as DHA C22:6 (in TG50:6, TG52:7, TG58:9, TG58:8, TG58:7), DPA C22:5 (in TG58:7) and ALA C18:3 (in TG50:4), compared with FM. These differences were the highest between colostrum and FM dedicated for infants aged 0–6 months but also between HM collected after 12 months postpartum and the corresponding FM (e.g., TG58:8 (fc = 45) or TG 56:6 (fc = 53), TG58:7 (22:5-18:1-18:1) and TG58:7 (22:6-18:0/18:1)). In further lactation stages, the largest difference between HM and FM was observed in the content of TGs containing both MCFAs and LCFAs in its structure (e.g., TG 48:4 with an FA composition of 12:0-18:1-18:3 (fc for HM colostrum vs. FM 0–6 months = 5, fc for HM 0–6 months vs. FM 0–6 months = 26, fc for HM 6–12 months vs. FM 6–12 months = 17, fc for HM >12 months vs. FM >12 months = 70)). Moreover, the difference was observed in the fatty acid substituents of TG 50:2 between HM and FM samples, as shown in Figure S7 in the Supplementary Materials.

Different lipid patterns were also observed for SMs. In HM, the most abundant SM was SMd42:2 (15–34%), while it comprised, on average, only 2–4% of the total SMs in FMs. SMd39:1 comprising 11–15% of the SM fraction in soy-supplemented FM was detected in HM samples in a much lower amount (0.05–0.69%). These lipid species were also statistically significantly different (*p* < 0.01) between the studied groups of FM and HM samples concerning the lactation stages. In particular, a higher peak area fold change was observed between samples of growing-up FM and HM samples collected after 1 year (e.g., SM 42:2 (fc = 24.4 for HM colostrum vs. FM 0-6 months, and fc = 45.1 for HM >12 months vs. FM >12 months). Considering differences between colostrum and FM, the greatest differences corresponded to SMs with an LCFA residue attached to the d18:1 sphingoid base, such as SMd38:1 (fc = 40), SMd44:2 (detected only in HM), and SMd42:2 (fc = 24).

In the PE class, PE38:4 and PE36:2 were among the most abundant PEs in both HM and FM samples, with PE 38:4 comprising 36–52% of all PEs in HM, and 32–52% in FM. The percentage relative amounts of the ether analogues of glycerophosphoethanolamines were much lower in FM than in HM. Moreover, the contents of all the detected ether analogues were statistically significantly higher in HM samples than in FM (6–13% in colostrum HM, 1–3% in further lactation stages, <0.5% in FM).

A diversified lipid pattern was also observed for PCs. In HM samples, the percentage relative amounts of PC34:1 and PC32:0 were the highest in colostrum and were decreased in further stages of lactation along with an increase in percentage relative amounts of PC36:2 and PC34:2. In FM samples, percentage contribution of these components was almost equal in all FMs concerning the age range target. Regarding these lipids separately, the content of some PCs was statistically significantly higher in FM than in HM; however, that of the others was lower (e.g., the content of PC38:6 was higher in HM and that of PC33:1 was higher in FM).

The FM samples contained lipid species not detected in HM, such as TG66:18 or PI34. On the other hand, HM contained lipids that FM lacks or has at very low levels, such as PE-O36:5 and PE-O38-5, ether analogues of TGs, some LC-PUFAs TGs, SMs and PIs.

## 4. Discussion

Human milk is considered an ideal source of nutrients and bioactive compounds for a new-born; however, it remains a high-value liquid in further stages of lactation [36]. According to previous reports, many children are fed by formula milk at the early stage of their life [7,8]. Considering the reports presenting differences in the health outcomes of breastfed and formula-fed children and how these differences impact metabolism [20,21,22,23,24], it is reasonable to explore the chemical composition of both human milk and commercially available FM, especially from a longitudinal perspective.

FMs are designed to mimic human milk composition; therefore, various sources of lipids are added to FM to mimic the chemical composition and nutritional function of HM, such as vegetable (soy, palm, coconut, rapeseed), fish and microorganisms oils, but also whole caprine milk. The detailed lipid compositions of some of these oils are not known in the literature (e.g., oil from *Schizochytrium* sp.) and predicting the FM lipid composition is not possible based on the list of ingredients. This raises a question about the similarity of the lipid composition of FM, its bioavailability, fate in the digestive system, biological functions compared with HM, and how it affects infants. Evaluation of the impact of feeding type on an infant’s growth and development should be performed using knowledge about the chemical composition of the food. Therefore, the knowledge gap regarding the molecular composition of HM and FM should be fulfilled. This should also concern lipid composition because the biological roles of these lipid components are of great importance for infant proper growth and development [37].

Formula milk should fulfil the nutritional needs of the child regardless of the used fat source. Although the lipid profiles of FM differ based on the fat source, we compared HM and FM samples and classified them into the lactation stages and age range target. High variance in the lipid composition of both HM and FM hinders the comparative studies and can influence the obtained results, as the average value may not be representative. Such an approach also ignores the differences in the lipid composition of individual mothers, which can occur for specific lipids and would be considered an outlier, as we observed in this study. Therefore, we decided to analyse data with various approaches, including statistical tests conducted based on average values for specific groups of samples, heatmaps and PCA analyses based on individual values of each analysed sample.

The results of this study showed significant differences in the lipid composition between FM and HM samples, not only in the content of particular lipids calculated as a fold change but also in the proportion (percentage relative amount) of various lipids within specific classes of lipids.

Analysis of the lipid composition of the HM samples collected at various stages of lactation showed the most significant difference between colostrum samples and the remaining HM samples. Samples collected in periods from the first to the 19th month of lactation showed no clear pattern according to child age as designated by FM dedication, namely 0–6 months, 6–12 months and >12 months.

The individual differences were dominant among analysed samples. Based on statistical tests, specific lipid species within the lipid class undergo different trends during lactation (i.e., the content of some TGs is higher in colostrum samples than in mature HM samples); however, we observed a contrasting trend for other molecules. Additionally, the individual HM samples were characterised by a higher concentration of specific compounds than the other samples. It is not without meaning when we consider the individual nutritional requirements of growing and developing infants. The intra-population variance of the HM samples can be determined by many factors, including maternal diet. The influence of maternal diet on the HM fatty acid composition has been very well documented [38,39,40,41]; however, the correlation between other lipids and maternal diet still requires investigation. High variance in lipid composition between mothers observed in this study can also suggest that HM lipid composition changes according to the actual needs of the infant. Thus, designing formula milk based on the average lipid composition, even for a specific lactation stage to completely mimic the HM composition, is hindered.

Especially alarming is the distinct lipid composition of FM from colostrum and mature milk obtained during advanced lactation (after 1 year postpartum) or lack of some lipid compounds in FM. This condition can possibly lead to the loss of intake of biologically important lipids by infants when fed with starting formula milk and at an age after 12 months when only a small number of children are still breastfed. Our results also proved that, at the advanced lactation (after 1 year postpartum), lactation milk is still rich in lipid species. This includes easily digested and accessible sources of energy—triacylglycerols—and also lipid species containing LC-PUFAs, such as arachidonic acid (ARA, C20:4), linolenic acid (ALA, C18:3), docosahexaenoic acid (DHA, C22:6), docosapentaenoic acid (DPA, C22:5), eicosatetraenoic acid (ETA; C20:4) and adrenic acid (C22:4). LC-PUFAs are fatty acids known for their great nutritional value because they are important components of the brain and retina; for example, the concentration of DHA in an infant’s brain increases until at least 2 years of age [42,43].

On average, the most abundant TGs in the collected HM samples were as follows: TG52:2 (18:1-16:0-18:1), TG 52:3 (18:1-16:0-18:2), TG 50:2 (18:1-16:0-16:1) and TG 54:3 (18:1-18:1-18:1). It is a slightly different composition than that reported in other studies of the composition of TGs in HM [15,26,44,45]. The observed differences are likely associated with the inclusion of HM samples in this study collected at further lactation stages (>1 year postpartum) compared with the mentioned studies. However, the difference might also be associated with the diets of women of different nationalities. Thus, not only the FA content but also the general lipid composition of HM can vary worldwide with respect to maternal lifestyle and the environment; a phenomenon that has already been proven for polar metabolites [19].

In contrast to HM, differences in the lipid composition within FM samples were generally brand-specific and linked to a contained fat source. The analysed FM samples contained a distinct TG pattern compared with human milk. All of the studied FMs contained a higher content of MCTGs and SCTGs than HM. However, milk formula with the addition of whole caprine milk was characterised by a higher content of these compounds than FM with other sources of fat. MCTGs and SCTGs are added to formula milk to directly provide energy because of the lower level of lipase in new-born babies [46]. These compounds were not detected in human milk or were present at significantly lower concentration levels.

Another important aspect of HM and FM compositional differences is the positional distribution of fatty acids in triacylglycerols, palmitic acid in the *sn*-2 position and unsaturated fatty acids primarily at the *sn*-1,3 positions in the TG structure enhancing the fat and calcium absorption [47]. This unique stereochemistry of TG molecules is difficult to replicate in milk formulas that contain mainly fat originated from a blend of three or more vegetable oils and/or mammalian milk fats [48]. We could not evaluate the position distribution of fatty acids in TG structures but could determine the fatty acyl composition in TGs. We observed the presence of TG species of distinct FAs compared with that observed in HM. The presence of TGs with structures different from those in HM may cause the problems of constipation in infants [49,50]. One example of TG not present in HM can be TG 66:18 (22:6/22:6/22:6), which is probably derived from *Crypthecodinium cohnii* and detected in FM, described as rich in DHA. The composition of LC-PUFAs in TGs and the level of specific TGS in FM are distinct from that observed in HM; therefore, the bioavailability and digestibility, as well as the nutritional properties of TGs contained in FM should be investigated.

We also detected ether analogues of TGs in HM samples (TG-O). Their content was statistically significantly higher in the colostrum samples than in the mature HM samples. Importantly, these compounds were not detected in many milk formula samples; if they were, their content was statistically significantly lower than that in HM. Information about these components in the literature is scarce. Recently, it was shown by Yu et al. that alkylglycerol-type (AKG-type) ether lipids are specific lipid signals of breast milk that are essential for healthy adipose tissue development [51] and therefore the knowledge about their presence in HM and FM and nutritional role should be extended.

We also observed that formula milk is also poor in the ether analogues of glycerophosphoethanolamines. In many formulas, the concentration level was below the detection limit. In other formulas, the content was significantly lower than that in HM. The PE-Os detected in HM contain LC-PUFAs in the structure, especially 20:4. This is in accordance with previous studies by Moukarzel et al. showing that human milk plasmalogens are highly enriched in long-chain PUFAs [52]. We did not find any report describing the study of these lipid compounds in milk formulae. Plasmalogens are components of the brain and their concentration increases during infant development [43]. Therefore, it should be investigated if the lack of these lipid components in milk formula may impact the nutritional value of FM regarding the development of an infant’s brain, especially in the critical early period of life.

Our study showed that HM also contains a higher concentration of other glycerophospholipids containing LC-PUFAs than FM, which contains a higher content of saturated phospholipids than HM. Although these LC-PUFAs are provided to the child by FM in the TG structure, the TGs and phospholipids might not be equivalent to dietary sources of PUFAs with different metabolic FA handling. In particular, it was shown that the brain accretion of AA and DHA was more effective for dietary phospholipids containing AA and DHA than for TGs [53]. Moreover, the different stereospecific distribution of LC-PUFAs in dietary TGs (LC-PUFA in *sn*-1 and *sn*-3 position) and phospholipids (LC-PUFA in the sn2 position) raises speculation of the possible dissimilar role of LC-PUFAs in these categories of lipids [47].

Notably, we observed that phospholipids containing LC-PUFAs are present in the HM of all lactation stages. Interestingly, the content of many lipids known to be involved in the brain and neurological system development, such as PC and SMs, were higher in mature milk obtained during the advanced stage of lactation (after 1 year postpartum) than at earlier stages of lactation. For these compounds, the difference in the content between HM and FM was higher after the age of 1 year because the content of these compounds in FM was almost at the same level considering various age targets. The higher content of SMs in HM in the advanced lactation stage may be associated with the decreased number of those feeding with HM after introducing the complimentary food and providing the sufficient amount of these lipid compounds with HM.

Differences in the content of phospholipids—not only GPs but also SMs—in FM and HM may refer to the differences in neurocognitive development between formula-fed and breastfed infants [54]. Exogenous phospholipids cross the blood–brain barrier, and the supplementation of FM with phospholipids structurally similar to those in HM may be crucial to promote brain development and enhance cognition in formula-fed infants. It was shown that the supplementation of FM in bioactive lipids, such as phospholipids and gangliosides, affect brain growth, structure, chemistry and spatial learning in neonatal piglets [55]. Recently, formula milk was improved by the supplementation of bovine MFGM and PL-enriched materials which have positive health effects, including promising outcomes in terms of neurodevelopment and defence against infections [48]. Still, many commercially available FMs on the Polish market are not enriched by MFGM.

## 5. Conclusions

In this study, we employed a lipidomic approach to comprehensively and semi-quantitatively compare the lipid composition of HM at various lactation stages and FM with different age range targets. The results of our study clearly showed that human milk and formula milk vary within all milk lipid classes. Although HM lipid composition varies individually between mothers and in the stage of lactation, FM lipid content differs concerning the fat source and brand used. Human milk lipid components change over the course of lactation. However, no trend can be indicated because this process is lipid species-specific. We also observed a higher content of lipids related to neurodevelopment in samples collected after 1 year postpartum than at the earlier lactation stages. We did not observe similar changes in formula milk samples. HM contains different contributions of lipid species within specific classes of lipids than FM and contains a higher content of many biologically important lipids than FM, such as ether analogues of glycerophosphoethanolamines. Detailed knowledge about changes in the lipid composition of HM and FM, including a long-term perspective, is required to investigate the impact of lipid components on child health and development and to move from standardised nutritional protocols to tailored, individualised nutrition in infants [11].

## Figures and Tables

**Table 1:** Lipids statistically significantly different between the colostrum and further lactation stage samples (ANOVA unequal variance test, *p* < 0.01, multiple testing correction: Benjamini–Hochberg, colostrum versus HM 0–6 months, versus HM 6–12 months, and versus HM >12 months. Mann–Whitney test, *p* < 0.01, multiple testing correction: Benjamini-Hochberg, colostrum vs. mature HM (lactation stages other than colostrum considered as a mature HM)). The complete list is presented in the Supplementary Materials in Tables S8 and S9.

Compound	Average Peak Area Fold Change HM 0–6 Months (*n* = 10) vs. Colostrum (*n* = 11)	Average Peak Area Fold Change HM 6–12 Months (*n* = 8) vs. Colostrum (*n* = 11)	Average Peak Area Fold Change HM >12 Months (*n* = 16). vs. Colostrum (*n* = 11)	Average Peak Area Fold Change Mature HM (*n* = 34) vs. Colostrum (*n* = 11)
DG36:2	2.8 ^a^	2.8	2.7	2.7
LysoPC18:3	2.8	2.6	3.9	3.2
LysoPE18:2	3.5	3.4	4.5	3.9
PC30:0	−3.4	−3.1	−2.1	−2.7
PC32:0	−2.7	−3.9	−2.4	−2.8
PC34:0	−2.0	−3.1	−1.9	−2.2
PC-O34:1	−3.6	−5.3	−3.0	−3.6
PE-O36:5	−2.8	−4.7	−1.9	−2.6
PE-O38:5	−2.2	−3.5	−1.5 (ns)	−2.0
PI38:4	−3.4	−6.7	−3.6	−4.0
PS36:1	−3.1	−4.9	−2.6	−3.1
SMd34:1	−2.3	−3.4	−1.7	−2.1
SMd34:2	−2.9	−3.4	−2.2	−2.6
SMd42:0	3.6	3.6	9.6	5.8
TG40:0	4.2	2.9	7.1	5.0
TG42:0	3.1	2.5	5.9	4.0
TG42:1	5.4	3.7	6.7	5.5
TG44:1	3.6	2.7	4.9	3.9
TG44:2	5.9	3.3	6.7	5.6
TG46:1	2.1	1.7	3.2	2.5
TG46:2	3.6	2.3	4.3	3.6
TG48:2	2.2	1.8	2.8	2.4
TG48:3	3.9	2.5	4.2	3.6
TG56:5	−1.5 (ns)	−2.2	−1.8 (ns)	−1.8
TG56:6_1	−1.2 (ns)	−2.7	−1.5 (ns)	−1.6
TG58:2	−4.8	−4.9	−3.9	−4.4
TG58:3	−2.3	−2.3	−2.1	−2.2
TG58:5	−2.4	−4.4	−4.1	−3.5
TG58:6	−2.3	−3.3	−2.0	−2.4
TG60:2	−5.6	−3.8	−5.8	−5.2
TGO-50:1	−4.7	−7.9	−3.8	−4.5
TGO-52:1	−4.9	−4.5	−2.3 (ns)	−3.2
TGO-52:2	−3.4	−4.9	−3.1	−3.5

^a^ Negative fold change (fc) value means average higher peak area in colostrum samples than in other HM samples; positive fold change value means average higher peak area in other HM samples than in the colostrum samples. DG, diacylglycerol; PC, glycerophosphocholine; PC-O, ether analogue of glycerophosphocholine; PE, glycerophosphoethanoloamine; PE-O, ether analogue of glycerophoshoethanoloamine; PS, glycerophosphoserine, PI, glycerophosphoinositol; SM, sphingomyelin; TG, triacylglycerol; TG-O, ether analogue of triacyglycerol; ns, not statistically significant change.

**Table 2:** Characteristics of milk formula included in the study regarding the fat source and content of LC-PUFAs according to the product label.

Brand	Age Range Target (Months)	Fat Source	LC-PUFAs
FM1	1 (0–6)	palm oil, coconut oil, rapeseed oil, sunflower oil, soy lecithin, fish oil	EPA, DHA, ARA
	2 (6–12)	palm oil, coconut oil, rapeseed oil, sunflower oil, soy lecithin	α-linolenic acid
	3 (12–18)	palm oil, rapeseed oil, sunflower oil, soy lecithin	α-linolenic acid
FM2	1 (0–6)	palm oil, coconut oil, rapeseed oil, sunflower oil, soy lecithin, fish oil, sunflower lecithin	DHA
	2 (6–12)	palm oil, coconut oil, rapeseed oil, sunflower oil, soy lecithin, fish oil, sunflower lecithin	DHA, ARA
	3 (12–18)	palm oil, rapeseed oil, sunflower oil, soy lecithin, fish oil, sunflower lecithin	α-linolenic acid
FM3	1 (0–6)	rapeseed oil, sunflower oil, sunflower lecithin, caprine milk, high olein sunflower oil	α-linolenic acid, linoleic acid
	2 (6–12)	rapeseed oil, sunflower oil, sunflower lecithin, caprine milk, high olein sunflower oil	α-linolenic acid, linoleic acid
FM4	1 (0–6)	palm olein, coconut oil, soy oil, high olein sunflower oil, soy lecithin, DHA from *Crypthecodinium cohnii* ARA from *Mortierella* alpine	DHA, ARA, α-linolenic acid
	2 (6–12)	palm olein, coconut oil, soy oil, high olein sunflower oil, soy lecithin, DHA from oils from *Crypthecodinium cohnii*, ARA from *Mortierella* alpine	DHA, ARA, α-linolenic acid
	3 (12–18)	palm olein, coconut oil, soy oil, rapeseed oil, fish oil, soy lecithin	α-linolenic acid, linoleic acid
	1 MFGM (0–6) MFGM	palm olein, coconut oil, soy oil, high olein sunflower oil, soy lecithin, oils from *Crypthecodinium cohnii* and *Schizochytrium* sp.	DHA, ARA, α- linolenic acid, linoleic acid
FM5	1 (0–6), 2 (6–12), 3 (12–18)	palm oil, caprine milk, rapeseed oil, sunflower oil	
FM6	1 (0–6), 2 (6-12)	sunflower oil, coconut oil, rapeseed oil, fish oil, soy lecithin	DHA, α-linolenic acid, linoleic acid
	3 (12–18)	palm olein, rapeseed oil, sunflower oil, soy lecithin, fish oil, oil from *Mortierella* alpine	
FM7	1 (0–6)	caprine milk, palm oil, rapeseed oil, sunflower oil	
	2(6–12)	caprine milk, palm oil, rapeseed oil, sunflower oil	

ARA, arachidonic acid; DHA, docosahexaenoic acid; EPA, Eicosapentaenoic acid; MFGM, milk fat globule membrane.

**Table 3:** The short-list of statistically significantly different lipids between the samples of caprine whole milk-based FM and FM supplemented with soy lecithin, accordingly to the Mann–Whitney test unpaired (multiple testing correction: Benjamini–Hochberg, *p* < 0.01). The complete list is presented in Table S10 in the Supplementary Materials.

Compound	Average Peak Area Fold Change Caprine Whole Milk-Based FM (*n* = 7) vs. Soy Lecithin-Supplemented FM (*n* = 13)
DG36:2	−1.9 ^a^
DG36:3	−2.1
LysoPC14:0	−6.2
LysoPC16:0	−2.4
LysoPC18:1	−3.2
LysoPC18:2	−6.5
LysoPE18:1	−2.2
PC30:0	−2.7
PC32:1	−2.1
PC34:0	1.8
PC36:4	−5.8
SMd39:1	−4.4
SMd41:1	1.9
TG38:0	2.0
TG38:1	7.4
TG42:0	3.1
TG42:1	3.5
TG44:0	2.6
TG44:1	6.3
TG46:1	3.1
TG48:1	2.3
TG48:2	1.7
TG50:3	1.6
TG52:4	−1.6
TG52:5	−2.3
TG54:3	−1.6
TG54:4	−1.4
TG54:5	−1.5
TG54:6	−1.6
TG54:7	−2.9
TG56:3	−1.4
TG56:4	−1.4
PI34:2	−10.3
TG54:8	−3.1
PC36:5	−20.8
PE36:4	−4.7
PC-O34:1	1.9
PC34:0	1.8
SMd40:1_1	3.0
TG34:1	31.7
TG37:0	13.9
SMd42:2	2.1

^a^ Negative fold change value means higher average peak area in the soy lecithin supplemented FM samples than in the caprine whole milk based FM samples. Positive fold change value means higher peak area in caprine whole milk based FM samples than in the soy lecithin supplemented FM. DG, diacylglycerol; PC, glycerophosphocholine; PE, glycerophosphoethanoloamine; SM, sphingomyeline; TG, triacylglycerol.

**Table 4:** The short-list of lipids indicating a statistically significant difference between HM and FM samples in the different lactation stages and age range targets according to ANOVA unequal variance test (multiple testing correction: Benjamini–Hochberg, *p* < 0.01).

Lipid Name	Average Peak Area Fold Change Colostrum (*n* = 11) vs. FM 0–6 Months (*n* = 8)	Average Peak Area Fold Change HM 0-6 Months (*n* = 10) vs. FM 0–6 Months (*n* = 8)	Average Peak Area Fold Change HM 6- 12 Months (*n* = 8) vs. FM 6–12 Months (*n* = 7)	Average Peak Area Fold Change HM > 12 Months (*n* = 16) vs. FM >12 Months (*n* = 5)
LysoPC22:6	12.2 ^a^	15.1	ns	ns
PC28:0	−5.4	−9.6	−5.3	−4.8
PC29:0	−5.8	−15.0	−8.2	−8.4
PC31:0	ns	−3.9	−3.3	−3.0
PC33:0	ns	−4.7	−5.6	−3.5
PC33:1	−2.7	−5.5	−4.1	−2.9
PC33:1	−2.7	−5.5	−4.1	−2.9
PC36:4	−11.8	−6.3	−6.4	−3.7
PC36:5	−36.4	−13.9	−16.5	−15.4
PC38:6	13.6	5.1	ns	ns
PE-O36:5	33.5	11.9	12.0	25.4
PE-O38:7	79.9	26.6	nd in FM	nd in FM
PI34:3	−12.9	nd in HM	nd in HM	nd in HM
SMd36:0	5.8	18.2	30.9	73.5
SMd36:1	6.0	12.8	14.0	54.0
SMd36:2	4.9	3.6	4.5	8.4
SMd38:1	39.6	77.1	112.6	227.5
SMd40:0	−28.3	−19.5	−23.6	nd in HM
SMd40:1_1	2.8	7.0	9.1	19.6
SMd42:2	24.4	18.8	22.3	45.1
SMd42:3	108.3	39.2	ns	ns
SMd44:2	182.4	33.6	ns	ns
TG46:1	2.7	5.8	5.3	21.3
TG46:2	ns	6.8	5.3	44.1
TG46:3	ns	13.1	7.6	33.8
TG48:1	2.3	3.2	3.4	6.9
TG48:2	2.7	5.8	6.7	11.8
TG48:3	4.9	19.0	14.8	64.2
TG48:4	4.8	25.9	16.6	69.5
TG48:5	4.9	41.8	31.0	51.3
TG50:3	4.7	9.2	7.9	13.4
TG50:4	3.6	9.5	7.1	14.0
TG50:5	4.0	15.3	12.8	20.4
TG50:6	10.5	22.7	ns	54.9
TG52:7	7.8	22.7	ns	ns
TG56:6_1	53.4	43.8	ns	ns
TG56:7_2	13.2	9.1	7.2	27.2
TG56:8	11.8	12.9	ns	ns
TG58:2	−2.4	−11.5	−7.8	−6.7
TG58:6	74.1	31.6	17.1	20.3
TG58:7_2	19.4	15.0	9.9	31.1
TG58:8_1	45.4	45.3	ns	ns
TG58:8_2	12.5	15.5	ns	ns
TG60:2	−3.2	−17.6	−8.6	−13.8

^a^ Negative fc value means higher peak area in the FM samples than in the HM samples, while positive fc value means higher peak area in the HM samples than in the FM samples. nd, not detected; ns, not statistically significant different; PC, glycerophosphocholine; PC-O, ether analogue of glycerophosphocholine; PE, glycerophosphoethanoloamine; PE-O, ether analogue of glycerophoshoethanoloamine; PS, glycerophosphoserine; PI, glycerophosphoinositol; SM, sphingomyelin; TG, triacylglycerol; TGO, ether analogue of triacyglycerol.

## Supplementary Materials

The following are available online at https://www.mdpi.com/2072-6643/12/7/2165/s1, Inclusion criteria for HM sample donors. Table S1: Characteristics of the analysed milk samples. Figure S1: The fragmentation pattern for PIs, PSs and ether analogues of TGs and PEs. Table S2: List of the identified lipids included in the batch targeted extraction. Figure S2: The exemplary total ion chromatograms (TICs) of the lipid extracts of the FM and HM samples. Table S3: The percentage relative amount of lipid species within specific class of lipids in colostrum samples. Table S4: The percentage relative amount of lipid species within specific class of lipids in HM samples collected between 0 and 6 months. Table S5: The percentage relative amount of lipid species within specific class of lipids in HM samples collected between 7 and 12 months. Table S6: The percentage relative amount of lipid species within specific class of lipids in HM samples collected samples collected after 12 months. Table S7: The percentage relative amount of lipid species within specific class of lipids in FM samples. Table S8: The complete list of statistically significantly differences between the colostrum and further lactation stage samples. Table S9: The complete list of lipids statistically significantly different between the colostrum and further lactation stage samples. Table S10: The complete list of statistically significantly (*p* < 0.01) different lipids between the samples of caprine whole milk-based FM and FM supplemented with soy lecithin. Table S11: The complete list of lipids indicating a statistically significant (*p* < 0.01) difference between HM and FM samples in the different lactation stages and age range targets. Figure S3: Clustering shown as a heatmap for the top 80 TGs. Figure S4: Clustering shown as a heatmap for the PCs detected in HM and/or FM samples. Figure S5: Clustering shown as a heatmap for the SMs detected in HM and/or FM samples. Figure S6: Clustering shown as a heatmap for the PEs, PIs and PSs detected in HM and/or FM samples.
