# Supplementary material for: Comparative Lipidomic Study of Human Milk from Different Lactation Stages and Milk Formulas
Source: Nutrients. 2020 Jul 21;12(7):2165. doi: 10.3390/nu12072165 (PMC7401268; doi:10.3390/nu12072165)
Supplement: Supplementary file 1 [file nutrients-12-02165-s001.pdf]

Supplementary Materials

Comparative lipidomic study of human milk from different lactation stages, and milk formulas

Weronika Hewelt-Belka [1,*], Dorota Garwolińska [1], Michał Młynarczyk [1] and Agata Kot-Wasik [1]

[1] Department of Analytical Chemistry, Chemical Faculty, Gdańsk University of Technology
* Correspondence: weronika.belka@pg.edu.pl

Table of contents

Inclusion criteria for HM sample donors	S-2
Table S1. Characteristics of the analyzed milk samples.	S-2
Figure S1. The fragmentation pattern for PIs, PSs and ether analogues of TGs and PEs	S-4
Table S2. List of the identified lipids included in the batch targeted extraction.	S-5
Figure S2. The total ion chromatograms (TICs) of the lipid extracts of the FM and HM	S-11
Table S3. The % relative amount of lipid species within specific class of lipids in colostrum samples	S-12
Table S4. The % relative amount of lipid species within specific class of lipids in HM samples collected between 0 and 6 months	S-14
Table S5. The % relative amount of lipid species within specific class of lipids in HM samples collected between 6 and 12 months.	S-16
Table S6. The % relative amount of lipid species within specific class of lipids in HM samples collected after 12 months.	S-19
Table S7. The % relative amount of lipid species within specific class of lipids in FM samples	S-22
Table S8. The complete list of statistically significantly different between the colostrum and further lactation stage samples accordingly to the ANOVA unequal variance test	S-26
Table S9. Lipids statistically significantly different between the colostrum and further lactation stage HM samples accordingly to the Mann-Whitney test unpaired.	S-32
Table S10. The complete list of statistically significantly different lipids between the samples of caprine whole milk-based FM and FM supplemented with soy lecithin accordingly to the Mann-Whitney test unpaired.	S-35
Table S11. Lipids indicating a statistically significant (p <0.01) difference between HM and FM samples in the different lactation stages and age range targets.	S-38
Figure S3. Clustering shown as a heatmap for the top 80 TGs indicated in the test t.	S-46
Figure S4. Clustering shown as a heatmap for the PCs detected in HM and/or FM samples.	S-47
Figure S5. Clustering shown as a heatmap for the SMs detected in HM and/or FM samples.	S-47
Figure S 6. Clustering shown as a heatmap for the PEs, PIs and PSs detected in HM and/or FM samples.	S-48
Figure S7. The MS/MS spectra and FA distribution of the TG 50:2 detected in HM and FM samples.	S-49

Inclusion criteria for HM sample donors

Women and their children participating in the study had to meet the inclusion criteria:

- mothers of singleton infants breastfeeding exclusively/fully lactating for the first 6 months,
- breastfeeding mothers of singleton infants at age 6 months or higher (complementary foods starting at 6 months was permitted for all infants as per American Academy of Pediatrics (AAP) guidelines and World Health Organization recommendations as long as their sole milk source was maternal breast milk up to the age of 1 year,
- infants had to be ≥35 weeks' gestation and in good general health at the time of enrollment.

Subject exclusion criteria included:

- mothers diagnose: preexisting type I or II diabetes, hypertension, parathyroid disease, and uncontrolled thyroid disease.
- twins or multiple births
- infants < 35 weeks' gestation; with a history of 72 hours in the NICU; any inborn error of metabolism; the history of congenital anomalies; or a history of consuming 10% of their diet as formula at the time of enrollment
- combined feeding at the time of enrollment (i.e., partially breastfeeding and formula-feeding their infants).

Table S2. Characteristics of the analyzed milk samples.

Sample ID	Milk type	Lactation month	Lactation stage/age target	FM brand
**FM.1.1**[a]	FM	Not applicable	0-6	1
**FM.1.2**	FM	Not applicable	6-12	1
**FM.1.3**	FM	Not applicable	>12	1
**FM.2.1**	FM	Not applicable	0-6	2
**FM.2.2**	FM	Not applicable	6-12	2
**FM.2.3**	FM	Not applicable	>12	2
**FM.3.1**	FM	Not applicable	0-6	3
**FM.3.2**	FM	Not applicable	6-12	3
**FM.4.1**	FM	Not applicable	0-6	4
**FM.4.2**	FM	Not applicable	6-12	4
**FM.4.3**	FM	Not applicable	>12	4
**FM.4.1.FMGM**	FM	Not applicable	0-6	4
**FM.5.1**	FM	Not applicable	0-6	5
**FM.5.2**	FM	Not applicable	6-12	5
**FM.5.3**	FM	Not applicable	>12	5
**FM.6.1**	FM	Not applicable	0-6	6
**FM.6.2**	FM	Not applicable	6-12	6
**FM.6.3**	FM	Not applicable	>12	6
**FM.7.1**	FM	Not applicable	0-6	7
**FM.7.2**	FM	Not applicable	6-12	7
**W.1.1.E**	HM	6	0-6	Not applicable
**W.1.1.M**	HM	6	0-6	Not applicable
**W.1.1(4M).E**	HM	4	0-6	Not applicable
**W.1.1(4M).M**	HM	4	0-6	Not applicable
**W.2.1.M**	HM	4	0-6	Not applicable

**W.2.1.E**	HM	4	0-6	Not applicable
**W.3.3.M**	HM	18	>12	Not applicable
**W.3.3.E**	HM	18	>12	Not applicable
**W.4.2.E**	HM	7	6-12	Not applicable
**W.5.1.M**	HM	6	0-6	Not applicable
**W.5.1.E**	HM	6	0-6	Not applicable
**W.6.2.(8M).M**	HM	8	6-12	Not applicable
**W.6.2.(10M).M**	HM	10	6-12	Not applicable
**W.6.2.(8M).M2**	HM	8	6-12	Not applicable
**W.6.2.(10M).M2**	HM	10	6-12	Not applicable
**W.6.2.(10M).M2**	HM	10	6-12	Not applicable
**W.7.3.M**	HM	13	>12	Not applicable
**W.7.3.E**	HM	15	>12	Not applicable
**W.8.3.M**	HM	13	>12	Not applicable
**W.8.3.E**	HM	13	>12	Not applicable
**W.9.3.M**	HM	15	>12	Not applicable
**W.9.3.E**	HM	15	>12	Not applicable
**W.10.3.M**	HM	16	>12	Not applicable
**W.10.3.E**	HM	16	>12	Not applicable
**W.11.3.M**	HM	18	>12	Not applicable
**W.11.3.E**	HM	18	>12	Not applicable
**W.12.1.M**	HM	6	0-6	Not applicable
**W.12.1.E**	HM	6	0-6	Not applicable
**W.13.3.**	HM	19	>12	Not applicable
**W.13.3.R**	HM	19	>12	Not applicable
**W.14.3.M**	HM	14	>12	Not applicable
**W.14.3.M**	HM	14	>12	Not applicable
**W.15.2.M**	HM	10	6-12	Not applicable
**W.15.2.E**	HM	10	6-12	Not applicable
**W.16.0**	HM	0	Colostrum	Not applicable
**W.17.0**	HM	0	Colostrum	Not applicable
**W.18.0**	HM	0	Colostrum	Not applicable
**W.19.0**	HM	0	Colostrum	Not applicable
**W.20.0**	HM	0	Colostrum	Not applicable
**W.21.0**	HM	0	Colostrum	Not applicable
**W.22.0**	HM	0	Colostrum	Not applicable
**W.23.0**	HM	0	Colostrum	Not applicable
**W.24.0**	HM	0	Colostrum	Not applicable
**W.25.0**	HM	0	Colostrum	Not applicable
**W.26.0**	HM	0	Colostrum	Not applicable

[a] FM1.1, sample of formula milk from brand 1 dedicated for the 0-6 mo. age range target; FM1.2, sample of formula milk from brand 1 dedicated for the 6-12 mo. age range target; FM1.3, sample of formula milk from brand 1 dedicated for the > 12 mo. age range target; [b] W.16.0, sample of human milk collected from woman with the ID no. 1 in the lactation stage HM colostrum; W.1.1.E, sample of human milk collected from woman with the ID no. 1 in the lactation stage (0-6 mo.) in the evening; W.1.1.M, sample of human milk collected from woman with the ID no. 1 in the lactation stage (0-6 mo.) in the morning;

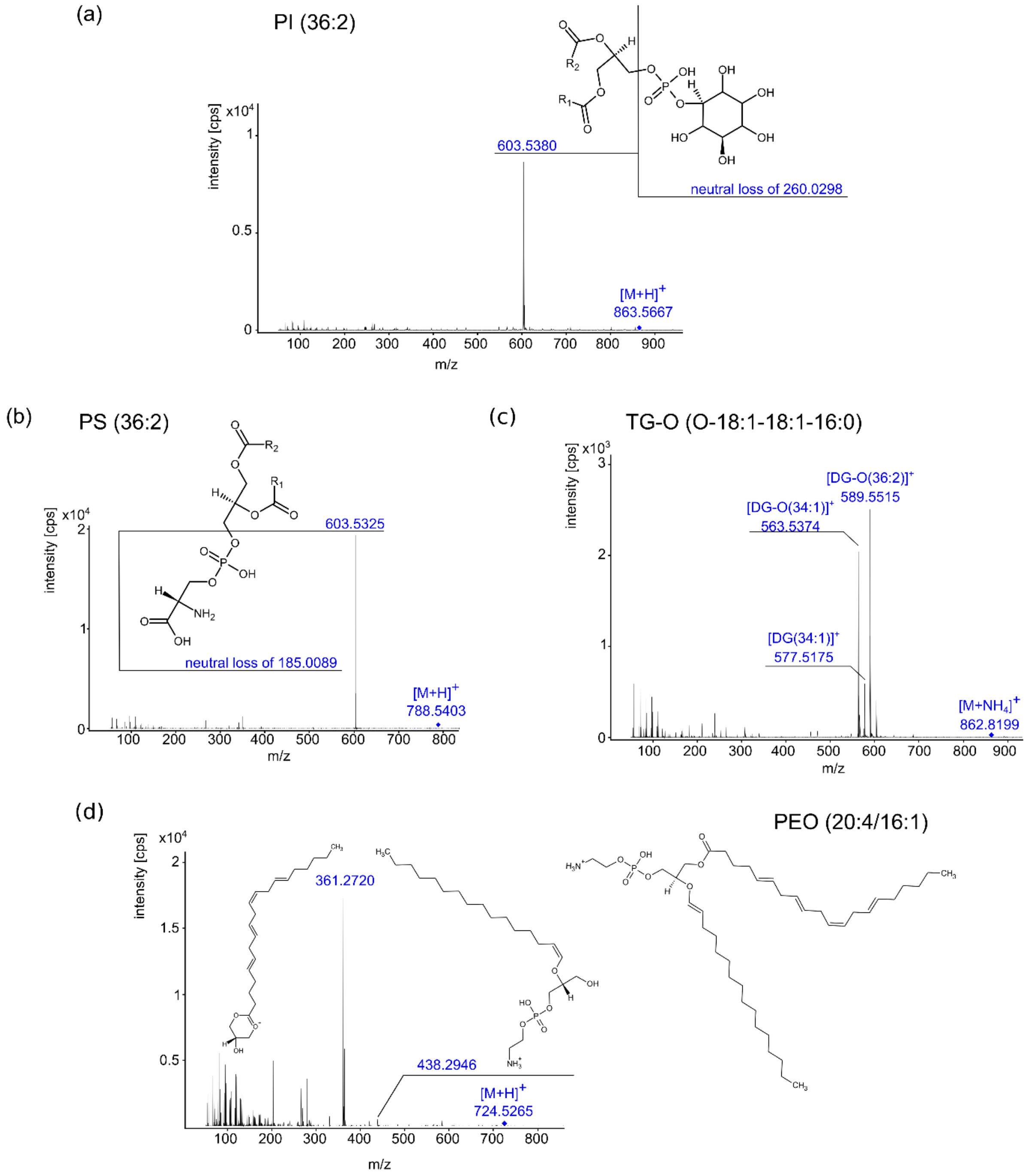

Figure S1. The fragmentation pattern for PIs, PSs and ether analogues of TGs and PEs

Table S2. List of the identified lipids included in the batch targeted extraction.

Lipid name	Formula	Retention time [min]	Measured mass[a]	Fatty acid composition
**DG18:0**	C21 H40 O5	2.76	394.268	
**DG20:0**	C23 H44 O5	3.29	422.2997	
**DG22:0**	C25 H48 O5	4.25	450.3306	
**DG24:0**	C27 H52 O5	5.31	478.3612	
**DG32:0**	C35 H68 O5	13.34	590.4877	
**DG32:1**	C35 H66 O5	11.60	588.4717	
**DG34:0**	C37 H72 O5	15.71	618.5182	
**DG34:1**	C37 H70 O5	13.93	616.5023	
**DG34:2**	C37 H68 O5	12.47	614.4865	
**DG36:1**	C39 H74 O5	16.70	644.5332	
**DG36:2**	C39 H72 O5	14.74	642.5172	
**DG36:3**	C39 H70 O5	13.46	640.5025	
**DG36:4**	C39 H68 O5	11.77	638.4865	
**LysoPC12:0**	C20 H42 N1 O7 P1	1.50	439.2688	
**LysoPC14:0**	C22 H46 N1 O7 P1	1.78	467.3627	
**LysoPC16:0**	C24 H50 N1 O7 P1	2.11	495.3314	
**LysoPC16:1**	C24 H48 N1 O7 P1	1.88	493.3155	
**LysoPC18:0**	C26 H54 N1 O7 P1	2.58	523.3620	
**LysoPC18:1**	C26 H52 N1 O7 P1	2.25	521.3468	
**LysoPC18:2**	C26 H50 N1 O7 P1	1.99	519.3336	
**LysoPC18:3**	C26 H48 N1 O7 P1	1.80	517.3150	
**LysoPC22:6**	C30H50NO7P	1.94	567.3324	
**LysoPCO-18:0/LysoPEO-21:0**	C26 H54 N1 O6 P1	2.55	507.3675	
**LysoPE18:0**	C23 H48 N1 O7 P1	2.56	481.3154	
**LysoPE18:1**	C23 H46 N1 O7 P1	2.23	479.2999	
**LysoPE18:2**	C23 H44 N1 O7 P1	1.98	477.2855	
**MG16:0**	C19H38O4	2.59	352.2575	
**MG18:0**	C21H42O4	3.29	380.2886	
**MG18:1**	C21H40O4	2.76	378.2731	
**PA46:3**	C49H91O8P	17.10	838.6451	
**PC24:0**	C32 H64 N1 O8 P1	3.82	621.4350	
**PC26:0**	C34 H68 N1 O8 P1	4.78	649.4668	
**PC28:0**	C36 H72 N1 O8 P1	6.07	677.4979	
**PC29:0**	C37 H74 N1 O8 P1	6.90	691.5127	
**PC30:0**	C38 H76 N1 O8 P1	7.60	705.5292	
**PC30:1**	C38 H74 N1 O8 P1	6.60	703.5142	
**PC31:0**	C39 H78 N1 O8 P1	8.60	719.5448	
**PC32:0**	C40 H80 N1 O8 P1	9.30	733.5607	
**PC32:1**	C40 H78 N1 O8 P1	8.10	731.5518	
**PC32:2**	C40 H76 N1 O8 P1	7.10	729.5299	
**PC33:0**	C41 H82 N1 O8 P1	10.40	747.5759	
**PC33:1**	C41 H80 N1 O8 P1	9.20	745.5607	
**PC33:2**	C41 H78 N1 O8 P1	8.07	743.5440	

**PC34:0**	C42 H84 N1 O8 P1	11.54	761.5923	
**PC34:1**	C42 H82 N1 O8 P1	10.10	759.5760	
**PC34:2**	C42 H80 N1 O8 P1	8.84	757.5607	
**PC34:3**	C42 H78 N1 O8 P1	7.75	755.5459	
**PC35:1**	C43 H84 N1 O8 P1	11.30	773.5911	
**PC35:2**	C43 H82 N1 O8 P1	9.90	771.5763	
**PC36:1**	C44 H86 N1 O8 P1	12.33	787.6074	
**PC36:2**	C44 H84 N1 O8 P1	10.89	785.5917	
**PC36:3**	C44 H82 N1 O8 P1	9.48	783.5763	
**PC36:4**	C44 H80 N1 O8 P1	8.40	781.5603	
**PC36:5**	C44 H78 N1 O8 P1	7.15	779.5427	
**PC36:6**	C44 H76 N1 O8 P1	6.25	777.5279	
**PC38:3**	C46 H86 N1 O8 P1	11.76	811.6069	
**PC38:4**	C46 H84 N1 O8 P1	10.30	809.5917	18:0-20:4
**PC38:6**	C46 H80 N1 O8 P1	8.70	805.5609	16:-22:6
**PCO-34:1**	C42 H84 N1 O7 P1	11.60	745.5968	
**PE34:1**	C39 H76 N1 O8 P1	10.11	717.5281	
**PE34:2**	C39 H74 N1 O8 P1	8.78	715.5135	
**PE36:0**	C41 H82 N1 O8 P1	13.680211	747.5785	
**PE36:1**	C41 H80 N1 O8 P1	12.24	745.5606	
**PE36:2**	C41 H78 N1 O8 P1	10.82	743.5448	
**PE36:3**	C41 H76 N1 O8 P1	9.49	741.5280	
**PE36:4**	C41 H74 N1 O8 P1	8.10	739.5123	
**PE38:4**	C43 H78 N1 O8 P1	10.93	767.5447	20:4-18:0
**PE40:4**	C45 H82 N1 O8 P1	12.70	795.5779	22:6-18:0
**PE40:6**	C45 H78 N1 O8 P1	10.68	791.5448	
**PEO-34:2**	C39 H76 N1 O7 P1	11.41	701.5364	
**PEO-36:5**	C41 H74 N1 O7 P1	10.06	723.5186	20:4-16:1
**PEO-38:5**	C43 H78 N1 O7 P1	12.27	751.5500	20:4-18:1
**PEO-38:7**	C43 H74 N1 O7 P1	9.90	747.5188	
**PEO-40:7**	C45H78N1O7P1	11.97	775.5506	
**PI34:2**	C43 H79 O13 P1	5.65	834.5238	
**PI34:3**	C43 H77 O13 P1	4.95	832.5066	
**PI36:1**	C45 H85 O13 P1	8.10	864.5600	
**PI36:2**	C45 H83 O13 P1	6.90	862.5535	
**PI36:3**	C45H81O13P1	6.17	860.5402	
**PI36:4**	C45 H79 O13 P1	5.22	858.5222	
**PI38:3**	C47 H85 O13 P1	7.66	888.5700	
**PI38:4**	C47 H83 O13 P1	7.13	886.5540	
**PS36:1**	C42 H80 N1 O10 P1	8.60	789.5497	
**PS36:2**	C42 H78 N1 O10 P1	7.48	787.5348	
**PS40:6**	C46 H78 N1 O10 P1	7.30	835.5341	
**SMd28:1**	C33 H67 N2 O6 P1	3.81	618.4716	
**SMd30:1**	C35 H71 N2 O6 P1	4.77	646.5030	
**SMd32:0**	C37 H77 N2 O6 P1	6.53	676.5496	

**SMd32:1**	C37 H75 N2 O6 P1	5.98	674.5349	d18:1/14:0
**SMd32:2**	C37 H73 N2 O6 P1	5.12	672.5187	
**SMd33:1/SMt32:2**	C38 H77 N2 O6 P1	6.70	688.5500	
**SMd34:0**	C39 H81 N2 O6 P1	8.20	704.5815	
**SMd34:1**	C39 H79 N2 O6 P1	7.47	702.5662	d18:1/16:0
**SMd34:2**	C39 H77 N2 O6 P1	6.50	700.5513	d18:1/16:1
**SMd36:0**	C41 H85 N2 O6 P1	10.05	732.6125	
**SMd36:1**	C41 H83 N2 O6 P1	9.30	730.5976	d18:1/18:0
**SMd36:2**	C41 H81 N2 O6 P1	8.10	728.5818	
**SMd38:0**	C43 H89 N2 O6 P1	12.65	760.6435	
**SMd38:1**	C43 H87 N2 O6 P1	11.44	758.6288	d18:1/20:0
**SMd39:0**	C44 H91 N2 O6 P1	13.81	774.6584	
**SMd39:1**	C44 H89 N2 O6 P1	13.10	772.6429	
**SMd39:2**	C44 H87 N2 O6 P1	11.48	770.6270	
**SMd40:0**	C45 H93 N2 O6 P1	15.18	788.6742	
**SMd40:1 _1**	C45 H91 N2 O6 P1	13.83	786.6596	d18:1/22:0
**SMd40:1 _2**	C45 H91 N2 O6 P1	14.25	786.6599	
**SMd40:2**	C45 H89 N2 O6 P1	12.10	784.6436	
**SMd41:1**	C46 H93 N2 O6 P1	15.10	800.6759	d18:1/23:0
**SMd41:2**	C46 H91 N2 O6 P1	13.57	798.6587	
**SMd42:0**	C47 H97 N2 O6 P1	17.43	816.7052	
**SMd42:1**	C47 H93 N2 O6 P1	16.37	814.6912	d18:1/24:0
**SMd42:2**	C47 H93 N2 O6 P1	14.17	812.6753	d18:1/24:1
**SMd42:3**	C47 H91 N2 O6 P1	12.60	810.6598	
**SMd43:2**	C48H96N2O6P	15.40	826.6915	
**SMd44:1**	C49 H99 N2 O6 P1	19.10	842.7219	
**SMd44:2**	C49 H97 N2 O6 P1	16.80	840.7064	
**SMt34:1**	C39 H79 N2 O7 P1	6.71	718.5599	
**SMt42:1**	C47 H95 N2 O7 P1	12.90	830.6855	
**TG26:0**	C29 H54 O6	6.64	498.4171	
**TG28:0**	C31 H58 O6	8.51	526.4485	
**TG30:0**	C33 H62 O6	10.82	554.4798	
**TG30:1**	C33 H60 O6	9.44	552.4629	
**TG31:0**	C34 H64 O6	8.52	568.4948	
**TG32:0**	C35 H66 O6	13.10	582.5107	
**TG32:1**	C35 H64 O6	11.68	580.4952	
**TG33:0**	C36 H68 O6	14.41	596.5255	
**TG33:1**	C37 H72 O6	13.00	594.5092	
**TG34:0**	C37 H70 O6	15.56	610.5414	
**TG34:1**	C37 H68 O6	14.00	608.5266	
**TG34:2**	C37 H66 O6	12.45	606.5109	
**TG35:0**	C38 H72 O6	17.00	624.5567	
**TG36:0**	C39 H74 O6	18.30	638.5732	12:0-12:0-12:0
**TG36:1**	C39 H72 O6	16.75	636.539	
**TG37:0**	C40 H76 O6	19.62	652.589	
**TG38:0**	C41 H78 O6	20.92	666.6048	14:0-12:0-12:0
**TG38:1**	C41 H76 O6	19.46	664.5904	
**TG38:2**	C41 H74 O6	17.63	662.5722	

**TG38:3**	C41 H72 O6	15.99	660.5578	
**TG39:0**	C42 H80 O6	22.43	680.6189	
**TG39:1**	C42 H78 O6	20.70	678.6042	
**TG40:0**	C43 H82 O6	23.50	694.6362	14:0-12:0-14:0
**TG40:1**	C43 H80 O6	21.83	692.6199	
**TG40:2**	C43 H78 O6	20.25	690.6052	
**TG40:3**	C43 H76 O6	18.77	688.5878	
**TG41:0**	C44 H84 O6	24.88	708.6512	
**TG41:1**	C44 H82 O6	23.31	706.6342	
**TG42:0**	C45 H86 O6	25.98	722.6672	14:0/14:0/14:0, 14:0/16:0/12:0
**TG42:1**	C45 H84 O6	24.45	720.6518	12:0/14:0/16:1
**TG42:2**	C45 H82 O6	22.76	718.6362	
**TG42:3**	C45 H80 O6	21.13	716.6207	
**TG43:0**	C46 H88 O6	27.16	736.6837	
**TG43:1**	C46 H86 O6	25.76	734.6673	
**TG44:0**	C47 H90 O6	28.32	750.6985	16:0/14:0/14:0, 18:0/14:0/12:0
**TG44:1**	C47 H88 O6	26.86	748.6829	18:1/16:0/10:0, 10:0/12:0/22:1
**TG44:2**	C47 H86 O6	25.27	746.6675	18:2/14:0/12:0
**TG44:3**	C47 H84 O6	23.84	744.6511	
**TG45:1**	C48 H90 O6	28.00	762.6981	
**TG46:0**	C49 H94 O6	30.53	778.7294	16:0/14:0/16:0
**TG46:1**	C49 H92 O6	29.12	776.7138	16:1/14:0/14:0, 12:0/10:0/22:1
**TG46:2**	C49 H90 O6	27.65	774.6987	18:2/16:0/12:0, 14:0/12:0/20:2
**TG46:3**	C49 H88 O6	26.28	772.6807	
**TG46:4**	C49 H86 O6	24.71	770.6664	
**TG47:1**	C50 H94 O6	30.31	790.729	
**TG47:2**	C50 H92 O6	28.91	788.7117	
**TG47:3**	C50 H90 O6	27.43	786.6961	
**TG48:0**	C51 H98 O6	32.54	806.7603	
**TG48:1**	C51 H96 O6	31.27	804.7461	18:1/16:0/14:0
**TG48:2**	C51 H94 O6	29.72	802.7282	
**TG48:3**	C51 H92 O6	28.45	800.7139	18:1/18:2/12:0
**TG48:4**	C51 H90 O6	26.90	798.6985	12:0/18:1/18:3
**TG48:5**	C51 H88 O6	25.41	796.6825	18:3/18:2/12:0
**TG49:1**	C52 H98 O6	32.56	818.7591	
**TG49:2**	C52 H96 O6	31.01	816.7441	
**TG50:1**	C53 H100 O6	33.31	832.777	18:1/16:0/16:0
**TG50:2**	C53 H98 O6	32.00	830.7614	18:1/16:0/16:1
**TG50:3**	C53 H96 O6	30.61	828.7452	18:1/18:2/16:0
**TG50:4**	C53 H94 O6	29.15	826.7295	18:2/18:3/18:1
**TG50:5**	C53 H92 O6	27.78	824.7147	18:2/18:3/14:0
**TG50:6**	C53 H90 O6	27.34	822.696	22:6/16:0/12:0

**TG51:1**	C54 H100 O6	34.30	846.7903	
**TG51:2**	C54 H98 O6	33.03	844.7765	18:1/17:1/16:0
**TG51:3**	C54 H96 O6	31.66	842.7605	
**TG51:4**	C54 H94 O6	30.60	840.7453	
**TG52:1**	C55 H104 O6	35.27	860.8077	18:0/18:1/16:0
**TG52:2**	C55 H102 O6	34.02	858.7925	18:1/16:0/18:1
**TG52:3**	C55 H100 O6	32.70	856.7767	18:1/16:0/18:2
**TG52:4**	C55 H98 O6	31.32	854.7608	18:2/18:2/16:0
**TG52:5**	C55 H96 O6	29.87	852.745	18:2/18:3/16:0
**TG52:6**	C55 H94 O6	28.56	850.7293	14:0/16:0/22:6
**TG52:7**	C55 H92 O6	28.13	848.7121	22:6/18:1/12:0
**TG53:1**	C56 H106 O6	36.34	874.8208	
**TG53:2**	C56 H104 O6	35.21	872.8068	
**TG53:3**	C56 H102 O6	33.72	870.7907	
**TG53:6**	C56 H96 O6	29.61	864.7449	
**TG54:1**	C57 H108 O6	37.13	888.8376	16:0/18:1/20:0
**TG54:2**	C57 H106 O6	36.04	886.9232	
**TG54:3**	C57 H104 O6	34.72	884.808	18:1/18:1/18:1
**TG54:4**	C57 H102 O6	33.42	882.7925	18:1/18:1/18:2
**TG54:5**	C57 H100 O6	32.07	880.7766	18:1/18:2/18:2, 18:3/18:1/18:1
**TG54:5 _2**	C57 H100 O6	32.73	880.7763	20:4/18:1/16:0
**TG54:6**	C57 H98 O6	30.66	878.7602	18:2/18:2/18:2 (FM), 18:1-18:2-18:3 (HM)
**TG54:7**	C57 H96 O6	29.17	876.7424	18:2/18:3/18:2
**TG54:7_2**	C57 H96 O6	30.31	876.7451	
**TG54:8**	C57 H94 O6	27.72	874.7281	18:3/18:3/18:2
**TG55:2**	C58 H108 O6	36.96	900.8392	
**TG55:3**	C58 H106 O6	35.73	898.821	
**TG56:1**	C59 H112 O6	38.96	916.8695	
**TG56:2**	C59 H110 O6	37.74	914.854	
**TG56:3**	C59 H108 O6	36.54	912.8386	20:1/18:1/18:1
**TG56:4**	C59 H106 O6	35.56	910.8223	
**TG56:5**	C59 H104 O6	34.22	908.8075	18:1/16:0/22:4
**TG56:6_2**	C59 H102 O6	33.60	906.7914	18:1/18:1/20:4
**TG56:6_1**	C59 H102 O6	33.03	906.7914	16:0/18:1/22:5
**TG56:7_2**	C59 H100 O6	32.49	904.775	22:6/18:1/16:0
**TG56:7_1**	C59 H100 O6	31.79	904.7757	
**TG56:8**	C59 H98 O6	31.01	902.7599	
**TG56:9**	C59 H96 O6	29.61	900.7456	
**TG57:2**	C60 H112 O6	38.79	928.8691	
**TG58:1**	C61 H112 O6	40.63	944.9016	
**TG58:10**	C61 H98 O6	30.31	926.7591	
**TG58:11**	C61 H96 O6	29.54	924.7444	
**TG58:2**	C61 H114 O6	39.57	942.8862	
**TG58:3**	C61 H112 O6	38.44	940.8701	
**TG58:4**	C61 H110 O6	37.30	938.8531	
**TG58:5**	C61 H108 O6	35.82	936.8375	22:4/18:1/18:1

**TG58:6**	C61 H106 O6	34.96	934.822	22:4/18:1/18:1
**TG58:7_2**	C61 H104 O6	34.51	932.8052	22:6/18:1/18:0
**TG58:7_1**	C61 H104 O6	33.68	932.807	22:5/18:1/18:1
**TG58:8_1**	C61 H102 O6	33.11	930.7905	18:1/18:1/22:6
**TG58:8_2**	C61 H102 O6	32.41	930.7915	
**TG58:9**	C61 H100 O6	31.79	928.7753	22:6/18:1/18:0
**TG59:2**	C62 H116 O6	40.35	956.8987	23:0/18:1/18:1
**TG60:1**	C63 H120 O6	42.20	972.9311	
**TG60:12**	C63 H98 O6	30.53	950.7604	
**TG60:2**	C63 H118 O6	41.24	970.916	
**TG60:3**	C63 H116 O6	40.27	968.9023	
**TG60:4**	C63 H114 O6	39.14	966.8838	
**TG60:5**	C63 H112 O6	38.09	964.8699	
**TG60:6**	C63 H110 O6	36.61	962.8534	22:4/18:1/20:1
**TG61:12**	C64 H100 O6	18.53	964.7694	
**TG62:3**	C65 H120 O6	41.48	996.9322	
**TG62:4**	C65 H118 O6	40.37	994.9149	
**TG62:5**	C65 H116 O6	39.32	992.8987	
**TG62:6**	C65 H114 O6	38.53	990.8852	
**TG62:7**	C65 H112 O6	37.31	988.8698	
**TG62:8**	C65 H110 O6	37.39	986.8511	
**TG63:2**	C66 H124 O6	41.19	1012.963	
**TG63:6**	C66 H116 O6	42.55	998.9437	
**TG64:4**	C67 H122 O6	41.85	1022.947	
**TG64:6**	C67 H118 O6	40.10	1018.916	
**TG64:8**	C67 H114 O6	39.23	1014.884	
**TG66:18**	C69 H98 O6	29.42	1022.757	22:6/22:6/22:6
**TGO-50:1**	C53 H102 O5	35.85	818.7943	
**TGO-52:1**	C55 H106 O5	37.73	846.826	
**TGO-52:2**	C55 H104 O5	36.50	844.8121	O-18:1-18:1-16:0
**TGO-54:2**	C57 H108 O5	38.27	872.843	
**TGO-54:3**	C57 H106 O5	37.13	870.8291	
**TGO-58:2**	C61 H116 O5	41.60	928.9016	

[a] measured mass calculated based on m/z value of observed ions on MS spectrum, [M+H]+ for SMs, PCs, PEs, PSs, PIs, [M+NH4]+ for TGs, [M+Na]+ for DGs and MGs. DG, diacylglycerol; MG, monoacylglycerol; PC, glycerophosphocholine; PC-O, ether analogue of glycerophosphocholine; PE, glycerophosphoethanoloamine; PE-O, ether anlogue of glycerophoshoethanoloamine; PS, glycerophosphoserine, PI, glycerophosphoinositol; SM, sphingomyelin; TG, triacylglycerol; TGO, ether analogue of triacyglycerol.

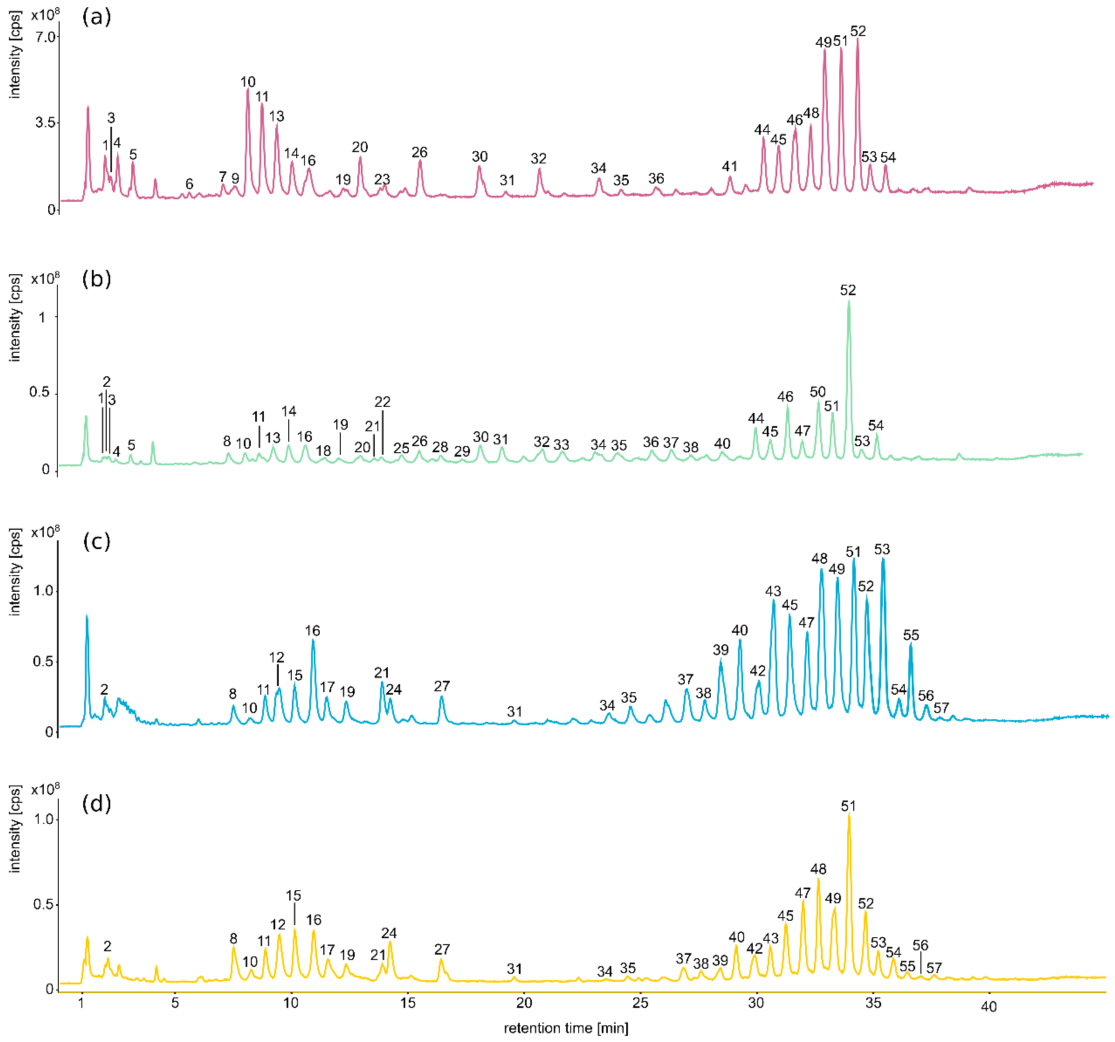

No.	Lipid	No.	Lipid	No.	Lipid	No.	Lipid	No.	Lipid	No.	Lipid
**1**	lyso PC 18:0	**11**	PC 34:2	**21**	SM d:40:1	**31**	TG 38:1	**41**	TG 54:7	**51**	TG 52:2
**2**	lyso PC 16:0	**12**	PC 32:0	**22**	TG 34:1	**32**	TG 38:0	**42**	TG 48:2	**52**	TG 54:3
**3**	lyso PC 18:1	**13**	PC 36:3	**23**	DG 34:1	**33**	TG 40:1	**43**	TG 50:3	**53**	TG 52:1
**4**	MG 16:0	**14**	PC 34:1	**24**	SM d:42:2	**34**	TG 40:0	**44**	TG 54:6	**54**	TG 54:2
**5**	MG 18:0	**15**	SM 32:1	**25**	SM 41:1	**35**	TG 42:1	**45**	TG 52:4	**55**	TG 56:3
**6**	PI 34:2	**16**	PC 36:2	**26**	TG 34:0	**36**	TG 42:0	**46**	TG 54:5	**56**	TG 58:4
**7**	PC 36:5	**17**	SM d:38:1	**27**	SM 42:1	**37**	TG 44:1	**47**	TG 50:2	**57**	TG 56:2
**8**	Sm d:34:1	**18**	TG 32:1	**28**	TG 36:1	**38**	TG 46:2	**48**	TG 52:3		
**9**	PC 30:0	**19**	PC 36:1	**29**	TG 38:2	**39**	TG 48:3	**49**	TG 50:1		
**10**	PC 36:4	**20**	TG 32:0	**30**	TG 36:0	**40**	TG 46:1	**50**	TG 54:4		

Figure S2. The total ion chromatograms (TICs) of the lipid extracts of the FM 4.2 (a), FM 3.1 (b), mature HM – W.13.3 (c), and colostrum HM – W.24.0 (c) samples analysed using RP-LC-Q-TOF-MS in the positive ionisation mode. Number corresponds to the identified lipids based on m/z value and interpretation of MS/MS spectra. DG, diacylglycerol; MG, monoacylglycerol; PC, glycerophosphocholine; PC-O, ether analogue of glycerophosphocholine; PE, glycerophosphoethanoloamine; PE-O, ether anlogue of glycerophoshoethanoloamine; PS, glycerophosphoserine, PI, glycerophosphoinositol; SM, sphingomyelin; TG, triacylglycerol; TGO, ether analogue of triacyglycerol.

Table S3. The % relative amount of lipid species within specific class of lipids in colostrum samples. The average value calculated for extraction duplicates is shown The empty space indicate that lipid specie was below the specified threshold (% contribution within class was lower than 1%, this include not detected species)

Compound	W.16.0	W.17.0	W.18.0	W.19.0	W.20.0	W.21.0	W.22.0	W.23.0	W.24.0	W.25.0	W.26.0
					**PC**						
**LysoPC16:0**	5%	8%	8%	4%	6%	4%	4%	4%	4%	3%	3%
**LysoPC18:0**	2%	3%	4%	2%	3%		2%	1%	3%	1%	1%
**LysoPC18:1**	4%	2%	4%	2%	3%	3%	2%	2%		2%	2%
**LysoPC18:2**	3%	2%	4%	3%	2%	2%	3%	3%	2%	1%	1%
**PC30:0**	2%	3%	4%	4%	2%	3%	2%	3%	4%	2%	2%
**PC32:0**	14%	27%	14%	11%	15%	13%	15%	16%	13%	16%	18%
**PC32:1**	2%		2%	2%	2%	2%	1%	2%	1%	2%	1%
**PC34:0**	4%	10%	6%	6%	6%	6%	6%	5%	6%	5%	6%
**PC34:1**	25%	14%	19%	18%	23%	22%	18%	19%	19%	25%	27%
**PC34:2**	10%	5%	9%	11%	9%	8%	12%	11%	11%	12%	8%
**PC36:1**	6%	4%	4%	5%	5%	7%	4%	4%	5%	5%	7%
**PC36:2**	13%	13%	13%	17%	13%	17%	18%	17%	19%	14%	13%
**PC36:3**	4%	2%	4%	6%	4%	5%	7%	7%	6%	5%	3%
**PC36:4**							1%	1%	1%		
**PCO-34:1**	3%	3%	3%	2%	3%	3%	2%	2%	2%	3%	5%
					**PE**						
**LysoPE18:0**		1%	3%		3%	1%			2%		
**LysoPE18:1**	9%	11%	10%	5%	9%	5%	4%	4%	5%	5%	4%
**PE34:1**	3%	2%	2%	2%	3%	2%	4%	3%	2%	3%	1%
**PE34:2**				1%			1%	1%	1%	1%	
**PE36:1**	6%	6%	6%	6%	6%	6%	4%	5%	6%	6%	8%
**PE36:2**	11%		16%	21%	9%	13%	15%	14%	14%	16%	13%
**PE36:3**	4%	2%	4%	6%	2%	3%	7%	7%	4%	5%	2%
**PE38:4**	48%	46%	40%	41%	45%	47%	49%	47%	50%	46%	42%
**PE40:6**		1%		3%	1%	6%	3%	3%	1%	1%	

**PEO-34:2**	3%	5%	3%	2%	3%	2%	2%	2%	2%	2%	4%
**PEO-36:5**	7%	13%	8%	6%	10%	7%	6%	7%	7%	8%	11%
**PEO-38:5**	5%	6%	4%	3%	5%	4%	3%	3%	3%	4%	9%
**PEO-38:7**	2%	3%	1%	2%	2%		1%	1%	1%	1%	2%
					**SM**						
**SMd32:1**	3%	1%	2%	3%	2%	2%	2%	2%	3%	2%	2%
**SMd34:1**	36%	19%	22%	19%	33%	30%	23%	21%	24%	31%	33%
**SMd36:0**										1%	
**SMd36:1**	6%	13%	9%	12%	7%	8%	10%	11%	4%	8%	7%
**SMd36:2**	2%		1%	1%	1%	1%	1%	1%		1%	
**SMd38:1**	6%	10%	9%	8%	5%	6%	7%	8%	6%	7%	7%
**SMd40:1_1**	9%	15%	15%	13%	9%	9%	12%	13%	13%	10%	13%
**SMd40:1_2**	3%		1%	1%	2%	1%	1%	1%	1%		2%
**SMd40:2**	1%	1%	3%	3%	2%	2%	1%	3%	1%	1%	1%
**SMd41:1**	3%	4%	4%	3%	3%	3%	3%	3%	3%	3%	3%
**SMd42:2**	21%	28%	25%	29%	26%	28%	31%	29%	34%	21%	22%
**SMd42:3**	3%	2%	3%	3%	4%	4%	4%	3%	4%	3%	3%
					**TG**						
**TG42:0**				2%				1%			
**TG42:1**				2%				1%			
**TG44:0**			1%	2%							
**TG44:1**	1%	2%	2%	4%		1%	2%	3%	2%		1%
**TG44:2**				1%							
**TG46:1**	3%	3%	5%	7%	2%	3%	4%	5%	3%	3%	3%
**TG46:2**	1%	1%	2%	2%		1%	2%	2%	1%		
**TG48:1**	4%	3%	5%	5%	4%	3%	3%	4%	4%	4%	5%
**TG48:2**	3%	3%	3%	4%	2%	3%	2%	3%	4%	2%	2%
**TG48:3**		1%	1%	2%		1%	1%	2%	2%		
**TG50:1**	7%	7%	7%	6%	7%	6%	7%	6%	5%	9%	9%
**TG50:2**	6%	9%	8%	8%	8%	7%	8%	8%	7%	8%	8%
**TG50:3**	2%	3%	3%	3%	3%	2%	3%	3%	3%	2%	2%

**TG52:1**	6%	3%	4%	3%	4%	4%	3%	3%	2%	5%	6%
**TG52:2**	19%	22%	20%	17%	23%	20%	20%	19%	17%	23%	20%
**TG52:3**	9%	13%	9%	9%	11%	10%	12%	12%	11%	10%	9%
**TG52:4**	2%	4%	2%	2%	3%	3%	3%	3%	4%	2%	2%
**TG54:2**	4%	2%	3%	2%	3%	4%	2%	2%	3%	3%	4%
**TG54:3**	8%	5%	6%	5%	8%	9%	6%	5%	8%	8%	8%
**TG54:4**	5%	4%	4%	3%	5%	5%	4%	4%	6%	5%	4%
**TG54:5**	2%	2%	1%	1%	2%	2%	2%	1%	3%	2%	2%

DG, diacylglycerol; MG, monoacylglycerol; PC, glycerophosphocholine; PC-O, ether analogue of glycerophosphocholine; PE, glycerophosphoethanoloamine; PE-O, ether anlogue of glycerophoshoethanoloamine; PS, glycerophosphoserine, PI, glycerophosphoinositol; SM, sphingomyelin; TG, triacylglycerol; TGO, ether analogue of triacyglycerol.

Table S4. The % relative amount of lipid species within specific class of lipids in HM samples collected between 0 and 6 months. The average value calculated for extraction duplicates is shown The empty space indicate that lipid specie was below the specified threshold (% contribution within class was lower than 1%, this include not detected species)

Compound	W.1.1(4M).E	W.1.1(4M).M	W.1.1.E	W.1.1.M	W.12.1.E	W.12.1.M	W.2.1.E	W.2.1.M	W.5.1.E	W.5.1.M
					**PC**					
**LysoPC16:0**	3%	4%	4%	3%	3%	4%	3%	3%	3%	6%
**Lyso-PC18:0**	2%	2%	2%	1%	3%	2%	2%	2%	2%	4%
**LysoPC18:1**	2%	2%	3%	2%	2%	2%	2%	2%	2%	3%
**LysoPC18:2**	4%	5%	6%	3%	6%	6%	5%	7%	7%	12%
**PC30:0**	1%	1%	1%	1%	2%	2%			1%	1%
**PC32:0**	8%	8%	8%	8%	7%	8%	6%	6%	7%	8%
**PC32:1**	1%	1%	1%	1%		1%				
**PC34:0**	4%	4%	3%	4%	4%	4%	4%	4%	3%	3%
**PC34:1**	17%	17%	18%	20%	15%	15%	13%	12%	13%	11%
**PC34:2**	11%	11%	12%	11%	13%	12%	13%	13%	13%	11%
**PC36:1**	5%	4%	4%	5%	4%	4%	4%	3%	3%	2%
**PC36:2**	28%	26%	23%	25%	26%	27%	31%	30%	29%	25%
**PC36:3**	10%	10%	9%	9%	9%	8%	10%	10%	11%	8%

**PC36:4**		2%	2%	1%	2%	1%	3%	2%	2%	2%
**PCO-34:1**	1%	1%	1%	1%		1%				
					**PE**					
**LysoPE18:1**	2%	3%	4%	4%	2%	4%	2%	3%	3%	5%
**LysoPE18:2**				1%	1%	2%			2%	2%
**PE34:1**	3%	3%	3%	3%	2%	2%	2%	3%	2%	2%
**PE34:2**	1%	1%	1%		1%	1%	2%	2%	1%	1%
**PE36:1**	6%	5%	6%	5%	4%	4%	5%	5%	3%	4%
**PE36:2**	25%	23%	27%	21%	26%	24%	27%	25%	22%	26%
**PE36:3**	8%	7%	9%	8%	10%	6%	8%	9%	9%	9%
**PE38:4**	45%	43%	41%	51%	45%	50%	46%	44%	50%	42%
**PE40:6**		5%			2%			2%	5%	3%
**PEO-34:2**	2%	1%	1%	1%						
**PEO-36:5**	3%	3%	3%	2%	2%	2%	2%	2%	1%	1%
**PEO-38:5**	2%	2%	2%	2%	1%	1%	1%	1%		
**PEO-38:7**										
					**SM**					
**SMd32:1**	2%	2%	2%	2%	3%	3%	2%	2%	3%	3%
**SMd34:1**	10%	10%	10%	10%	9%	12%	11%	10%	10%	11%
**SMd36:0**	1%	1%	1%		1%	1%	1%	2%	1%	1%
**SMd36:1**	16%	16%	18%	17%	18%	17%	15%	16%	14%	13%
**SMd36:2**						1%				
**SMd38:1**	13%	13%	13%	12%	14%	12%	12%	12%	14%	11%
**SMd40:1_1**	27%	26%	28%	29%	28%	25%	24%	26%	28%	31%
**SMd40:1_2**								2%		2%
**SMd40:2**	1%		1%	2%	2%	3%	2%	1%	2%	2%
**SMd41:1**	3%	4%	4%	5%	4%	4%	5%	4%	5%	5%
**SMd42:2**	21%	21%	18%	18%	17%	17%	23%	21%	18%	15%
**SMd42:3**	1%	1%	1%			2%	2%	1%		
					**TG**					
**TG40:0**					1%	1%				2%

**TG42:0**					1%	2%		1%	1%	2%
**TG42:1**	2%	2%	2%	2%	2%	2%	2%	2%	2%	2%
**TG44:0**					1%	2%		1%	1%	3%
**TG44:1**	3%	3%	3%	3%	4%	4%	3%	3%	3%	4%
**TG44:2**	1%	2%	1%		1%	1%	2%	2%	1%	1%
**TG46:1**	4%	4%	4%	3%	5%	6%	3%	4%	4%	6%
**TG46:2**	3%	3%	3%	3%	3%	3%	3%	3%	3%	3%
**TG48:1**	3%	2%	3%	3%	4%	5%	2%	3%	3%	5%
**TG48:2**	3%	2%	3%	4%	4%	5%	3%	3%	3%	4%
**TG48:3**	2%	3%	2%	2%	2%	2%	3%	2%	2%	2%
**TG50:1**	5%	2%	4%	3%	4%	6%	3%	4%	3%	6%
**TG50:2**	7%	5%	8%	6%	6%	8%	6%	7%	5%	6%
**TG50:3**	3%	3%	3%	2%	3%	3%	3%	3%	3%	2%
**TG52:1**	3%	1%	2%	3%	4%	4%	3%	3%	3%	6%
**TG52:2**	14%	14%	15%	17%	15%	17%	14%	15%	15%	14%
**TG52:3**	12%	14%	12%	9%	10%	9%	13%	13%	9%	7%
**TG52:4**	4%	5%	4%	3%	3%	2%	5%	5%	3%	2%
**TG54:2**	2%	1%	2%	3%	2%	2%	1%	1%	2%	2%
**TG54:3**	7%	5%	7%	13%	5%	4%	5%	4%	7%	5%
**TG54:4**	6%	6%	5%	7%	5%	3%	6%	4%	7%	4%
**TG54:5**	3%	4%	2%	2%	3%	1%	4%	3%	4%	2%

DG, diacylglycerol; MG, monoacylglycerol; PC, glycerophosphocholine; PC-O, ether analogue of glycerophosphocholine; PE, glycerophosphoethanoloamine; PE-O, ether anlogue of glycerophoshoethanoloamine; PS, glycerophosphoserine, PI, glycerophosphoinositol; SM, sphingomyelin; TG, triacylglycerol; TGO, ether analogue of triacyglycerol.

Table S5. The % relative amount of lipid species within specific class of lipids in HM samples collected between 6 and 12 months. The average value calculated for extraction duplicates is shown The empty space indicate that lipid specie was below the specified threshold (% contribution within class was lower than 1%, this include not detected species)

Compound	W.15.2.E	W.15.2.M	W.4.2.E	W.6.2.(10M).M	W.6.2.(10M).M2	W.6.2.(8M).M	W.6.2.(8M).M2
				**PC**			

**LysoPC16:0**	3%	3%	4%	4%	5%	4%	3%
**Lyso-PC18:0**	2%	3%	3%	2%	2%	2%	2%
**Lyso-PC18:1**	2%	2%	1%	3%	3%	1%	2%
**Lyso-PC18:2**	6%	6%	7%	6%	8%	7%	5%
**PC30:0**	2%	2%	1%	2%	1%	2%	2%
**PC32:0**	7%	6%	6%	9%	8%	8%	8%
**PC32:1**	1%	1%	1%	1%	1%	1%	1%
**PC34:0**	3%	3%	4%	4%	4%	4%	4%
**PC34:1**	13%	12%	14%	18%	17%	13%	17%
**PC34:2**	14%	13%	11%	14%	14%	13%	13%
**PC36:1**	4%	4%	3%	2%	3%	2%	3%
**PC36:2**	29%	31%	28%	21%	21%	25%	24%
**PC36:3**	8%	9%	11%	9%	8%	11%	10%
**PC36:4**	1%	1%	2%	1%		2%	2%
**PCO-34:1**			1%	1%	1%	1%	1%
**PE**							
**Lyso-PE18:0**		1%			1%		
**Lyso-PE18:1**	3%	3%	4%	5%	7%	5%	4%
**LYsoPE18:2**	1%	1%	2%	2%	3%	2%	1%
**PE34:1**	2%	1%	2%	3%	2%	2%	2%
**PE34:2**	2%	2%	1%	2%	1%		1%
**PE36:1**	4%	4%	5%	5%	4%	4%	4%
**PE36:2**	28%	28%	24%	25%	18%	24%	23%
**PE36:3**	8%	8%	7%	10%	6%	10%	9%
**PE38:4**	44%	43%	46%	41%	52%	46%	49%
**PE40:6**	5%	4%	2%				
**PEO-34:2**			1%	1%			1%
**PEO-36:5**	2%	2%	2%	2%	2%	2%	2%
**PEO-38:5**	1%	1%	1%	2%	2%	1%	1%
**SM**							
**SMd32:1**	3%	3%	2%	2%	2%	2%	2%

**SMd34:1**	10%	11%	9%	9%	11%	9%	10%
**SMd36:0**	1%	2%	1%	2%	1%	1%	2%
**SMd36:1**	19%	20%	15%	18%	18%	12%	16%
**SMd36:2**		1%			1%		
**SMd38:1**	13%	12%	12%	13%	11%	11%	12%
**SMd40:1_1**	27%	26%	26%	27%	23%	35%	27%
**SMd40:2**	3%	1%	2%	2%	3%		
**SMd41:1**	4%	4%	4%	6%	6%	5%	4%
**SMd42:2**	15%	15%	24%	17%	18%	20%	22%
**SMd42:3**	1%	1%	1%	1%	2%	1%	1%
				**TG**			
**TG40:0**	1%	2%				1%	
**TG42:0**	1%	2%	1%			2%	
**TG42:1**	2%	3%	2%	1%		3%	1%
**TG44:0**	1%	2%	1%			1%	
**TG44:1**	4%	6%	3%	3%	2%	4%	3%
**TG44:2**	1%	2%	1%			1%	
**TG46:1**	6%	8%	4%	5%	4%	6%	4%
**TG46:2**	3%	4%	3%	2%	2%	3%	2%
**TG48:1**	5%	5%	3%	4%	4%	4%	3%
**TG48:2**	3%	4%	5%	3%	4%	5%	4%
**TG48:3**	2%	2%	2%	2%	1%	3%	2%
**TG50:1**	4%	4%	4%	6%	4%	4%	3%
**TG50:2**	7%	7%	6%	8%	6%	6%	5%
**TG50:3**	3%	3%	3%	3%	2%	3%	2%
**TG52:1**	3%	2%	4%	3%	4%	3%	3%
**TG52:2**	16%	14%	14%	19%	22%	12%	15%
**TG52:3**	9%	7%	9%	10%	9%	8%	9%
**TG52:4**	2%	2%	3%	2%	2%	2%	3%
**TG54:2**	2%	2%	3%	3%	3%	2%	3%
**TG54:3**	6%	4%	6%	8%	10%	6%	11%

**TG54:4**	4%	2%	5%	4%	6%	5%	8%
**TG54:5**	2%		3%	1%	2%	3%	4%

DG, diacylglycerol; MG, monoacylglycerol; PC, glycerophosphocholine; PC-O, ether analogue of glycerophosphocholine; PE, glycerophosphoethanoloamine; PE-O, ether anlogue of glycerophoshoethanoloamine; PS, glycerophosphoserine, PI, glycerophosphoinositol; SM, sphingomyelin; TG, triacylglycerol; TGO, ether analogue of triacyglycerol.

Table S6. The % relative amount of lipid species within specific class of lipids in HM samples collected samples collected after 12 months. The average value calculated for extraction duplicates is shown The empty space indicate that lipid specie was below the specified threshold (% contribution within class was lower than 1%, this include not detected species)

Compound	W.10.3.E	W.10.3.M	W.11.3.E	W.11.3.M	W.13.3.	W.13.3.R	W.14.3.M	W.3.3.E	W.3.3.M	W.7.3.E	W.7.3.M	W.8.3.E	W.8.3.M	W.9.3.E	W.9.3.M
							**PC**								
**LysoPC16:0**	7%	3%	4%	3%	3%	3%	4%	4%	5%	3%	3%	4%	4%	4%	3%
**Lyso-PC18:0**	6%	3%	3%	3%	3%	3%	2%	6%	6%	2%	2%	2%	2%	5%	4%
**Lyso-PC18:1**	2%		1%	3%	3%	3%	2%	3%	4%	2%	2%	1%	1%	3%	
**Lyso-PC18:2**	10%	5%	6%	5%	6%	7%	6%	3%	5%	6%	7%	6%	6%	4%	4%
**PC30:0**	2%	2%	1%	1%	1%	1%		2%	2%	1%	2%	1%		2%	1%
**PC32:0**	6%	7%	6%	6%	4%	4%	7%	6%	5%	5%	6%	8%	8%	6%	5%
**PC32:1**		1%	1%	1%					1%		1%			1%	1%
**PC34:0**	3%	3%	3%	3%	2%	2%	4%	2%	2%	4%	4%	3%	3%	3%	3%
**PC34:1**	12%	16%	16%	15%	16%	16%	16%	19%	17%	14%	11%	18%	18%	18%	19%
**PC34:2**	12%	13%	13%	11%	12%	12%	13%	15%	14%	10%	11%	16%	16%	12%	13%
**PC36:1**	4%	5%	6%	6%	6%	7%	3%	6%	6%	4%	4%	3%	3%	6%	6%
**PC36:2**	23%	27%	25%	29%	27%	28%	25%	21%	21%	30%	31%	22%	22%	23%	26%
**PC36:3**	7%	9%	9%	9%	9%	8%	11%	7%	7%	10%	9%	9%	8%	7%	8%
**PC36:4**	2%	2%	2%	2%	2%	1%	2%	2%	2%	2%	2%	2%	2%	1%	2%
**PCO-34:1**			1%	1%			1%			1%	1%				

							**PE**								
**Lyso-PE18:0**	2%	1%	2%	1%				5%	5%			1%	1%	5%	2%
**Lyso-PE18:1**	7%	3%	4%		2%	2%	3%	3%	4%	3%	3%	5%	5%	4%	3%
**LYsoPE18:2**	2%	1%	1%	1%		1%	1%			1%	1%	1%	2%	1%	
**PE34:1**	2%	2%	2%	2%	2%	2%	2%	3%	2%	2%	2%	2%	2%	3%	2%
**PE34:2**	2%	2%	1%	1%	1%	1%	1%	2%	2%	1%	1%	2%	2%	2%	2%
**PE36:1**	4%	4%	5%	5%	4%	5%	4%	7%	6%	4%	4%	5%	5%	6%	5%
**PE36:2**	25%	27%	30%	30%	26%	28%	24%	27%	26%	25%	26%	23%	24%	27%	24%
**PE36:3**	8%	9%	8%	7%	9%	8%	10%	6%	7%	8%	9%	9%	9%	6%	7%
**PE38:4**	41%	44%	39%	43%	45%	42%	46%	36%	37%	46%	45%	46%	45%	41%	48%
**PE40:6**				2%	4%	5%	1%	2%	1%	5%	2%	1%			2%
**PEO-34:2**	1%	1%	1%	1%		1%		2%	2%	1%	1%			1%	
**PEO-36:5**	3%	3%	2%	2%	2%	2%	2%	2%	2%	2%	3%	2%	2%	2%	2%
**PEO-38:5**	2%	2%	2%	2%	1%	2%	2%	2%	2%	1%	1%	1%	2%	1%	1%
							**SM**								
**SMd32:1**	3%	2%	2%	2%	3%	3%	2%	4%	4%	2%	3%	2%	2%	3%	3%
**SMd34:1**	10%	9%	9%	9%	9%	10%	9%	9%	10%	8%	8%	11%	10%	9%	8%
**SMd36:0**	2%	2%	2%	1%	2%	2%	2%	2%	2%	2%	1%	1%	2%	2%	2%
**SMd36:1**	14%	15%	17%	16%	19%	18%	20%	14%	15%	16%	14%	17%	16%	15%	14%
**SMd38:1**	13%	13%	15%	14%	14%	14%	14%	11%	10%	11%	10%	13%	13%	10%	12%
**SMd40:1_1**	24%	28%	26%	29%	29%	27%	28%	24%	24%	30%	29%	25%	26%	26%	27%
**SMd40:1_2**								1%			1%				
**SMd40:2**	3%	2%	2%	1%	1%	2%	1%	1%	2%	2%	2%	3%	3%	1%	2%
**SMd41:1**	4%	4%	4%	4%	5%	5%	5%	4%	4%	4%	4%	6%	6%	6%	5%
**SMd42:2**	21%	19%	18%	19%	15%	15%	15%	23%	23%	21%	22%	18%	16%	22%	22%
**SMd42:3**	2%	1%	2%			1%	1%	1%	1%	1%	1%	2%	1%	1%	1%

							**TG**								
**TG40:0**	1%	1%	2%	1%	1%	1%		2%	1%		2%			3%	1%
**TG42:0**	2%	2%	2%	2%	2%	2%		4%	2%	1%	2%			3%	2%
**TG42:1**	2%	2%	2%	2%	2%	2%	2%	1%	2%	2%	3%	1%		3%	3%
**TG44:0**	2%	1%	2%	1%	2%	4%		6%	1%	1%	1%		1%	2%	2%
**TG44:1**	4%	5%	5%	4%	5%	4%	3%	3%	4%	4%	6%	2%	2%	5%	5%
**TG44:2**	1%	2%	2%	2%	1%	1%	1%	1%	2%	1%	2%			2%	2%
**TG46:1**	7%	7%	7%	6%	8%	7%	4%	5%	6%	5%	7%	3%	4%	7%	7%
**TG46:2**	3%	3%	3%	3%	3%	2%	2%	2%	3%	3%	4%	2%	2%	3%	3%
**TG48:1**	6%	5%	5%	4%	6%	7%	4%	7%	4%	4%	4%	3%	4%	6%	4%
**TG48:2**	4%	4%	4%	4%	4%	4%	4%	3%	3%	3%	4%	3%	3%	4%	3%
**TG48:3**	2%	2%	2%	3%	2%	1%	2%	2%	3%	2%	3%	1%	1%	2%	2%
**TG50:1**	6%	5%	5%	3%	6%	9%	4%	7%	3%	5%	3%	5%	9%	4%	5%
**TG50:2**	7%	7%	7%	7%	8%	7%	7%	6%	7%	6%	7%	6%	6%	6%	7%
**TG50:3**	3%	3%	3%	4%	3%	2%	3%	3%	4%	3%	3%	2%	2%	3%	3%
**TG52:1**	4%	3%	3%	2%	3%	10%	4%	8%	2%	4%	2%	7%	8%	3%	4%
**TG52:2**	15%	14%	14%	13%	14%	12%	17%	7%	12%	16%	12%	21%	19%	10%	14%
**TG52:3**	8%	9%	8%	10%	8%	6%	10%	5%	9%	10%	7%	9%	12%	6%	9%
**TG52:4**	3%	3%	2%	3%	3%	2%	2%	2%	3%	3%	2%	3%	3%	2%	2%
**TG54:2**	2%	2%	2%	2%	2%	2%	3%	4%	2%	2%	2%	4%	3%	2%	2%
**TG54:3**	4%	4%	4%	5%	3%	3%	8%	4%	5%	6%	5%	8%	6%	3%	4%
**TG54:4**	3%	3%	3%	4%	2%	2%	5%	3%	5%	5%	4%	5%	4%	2%	4%
**TG54:5**	1%	1%	1%	2%	1%		2%	2%	3%	2%	1%	3%	2%	1%	2%

DG, diacylglycerol; MG, monoacylglycerol; PC, glycerophosphocholine; PC-O, ether analogue of glycerophosphocholine; PE, glycerophosphoethanoloamine; PE-O, ether anlogue of glycerophoshoethanoloamine; PS, glycerophosphoserine, PI, glycerophosphoinositol; SM, sphingomyelin; TG, triacylglycerol; TGO, ether analogue of triacyglycerol.

Table S7. The % relative amount of lipid species within specific class of lipids in FM samples. The average value calculated for extraction duplicates is shown The empty space indicate that lipid specie was below the specified threshold (% contribution within class was lower than 1%, this include not detected species)

Compound	FM. 1.1	FM. 1.2	FM. 1.3	FM. 2.1	FM. 2.2	FM. 2.3	FM. 3.1	FM. 3.2	FM. 4.1	FM.4.1.F MGM	FM. 4.2	FM. 4.3	FM. 5.1	FM. 5.2	FM. 5.3	FM. 6.1	FM. 6.2	FM. 6.3	FM. 7.1	FM. 7.2
										**PC**										
**Lyso-PC14:0**	2%		2%	2%	1%	1%														
**LysoPC16:0**	6%	2%	8%	7%	4%	7%	2%	1%	3%	2%	2%	2%	3%	3%	4%	2%	3%	4%	1%	4%
**Lyso-PC18:0**	1%		1%	1%	1%	1%	1%		1%			1%	1%		1%		1%	1%		1%
**Lyso-PC18:1**	7%	2%	8%	8%	3%	7%	4%	1%	3%	2%	3%	2%	3%	3%	4%	2%	4%	4%	1%	1%
**Lyso-PC18:2**	5%	3%	7%	6%	4%	7%	3%	1%	11%	4%	6%	6%	1%	1%	2%	3%	8%	9%		2%
**PC28:0**	2%	2%	1%	2%	2%	1%				1%		1%	1%		1%	2%	1%	1%		
**PC30:0**	8%	8%	6%	8%	8%	5%	2%	2%	3%	5%	2%	4%	3%	3%	3%	7%	3%	3%	3%	3%
**PC31:0**	1%	2%	1%	1%	1%	1%	1%	1%		1%			1%	1%	1%	1%	1%		1%	1%
**PC32:0**	8%	5%	6%	8%	8%	6%	8%	8%	2%	6%	2%	4%	10%	10%	11%	8%	4%	3%	10%	10%
**PC32:1**	3%	4%	3%	3%	3%	3%	1%	1%	1%	2%	1%	2%	2%	2%	2%	3%	2%	1%	2%	2%
**PC33:0**		1%	1%		1%	1%		1%					1%	1%	1%	1%				1%
**PC33:1**	1%	1%	1%	1%	1%	1%	1%	1%		1%			1%	1%	1%	1%			1%	1%
**PC34:0**	2%	2%	2%	2%	2%	2%	4%	3%	1%	1%	1%	1%	4%	4%	4%	2%	1%	1%	4%	4%
**PC34:1**	17%	15%	14%	15%	16%	14%	19%	23%	7%	13%	9%	10%	27%	26%	26%	18%	11%	11%	29%	26%
**PC34:2**	8%	13%	9%	9%	10%	10%	11%	11%	16%	17%	19%	16%	8%	8%	7%	11%	16%	17%	7%	8%
**PC34:3**	1%	1%	1%	1%	1%	1%	1%	1%	2%	1%	1%	2%	1%	1%	1%	1%	1%	1%	1%	1%
**PC36:1**	4%	4%	3%	4%	4%	4%	5%	5%	2%	3%	2%	2%	6%	6%	5%	4%	2%	2%	7%	7%
**PC36:2**	8%	10%	8%	8%	9%	9%	15%	17%	8%	9%	8%	8%	17%	16%	15%	10%	9%	8%	19%	17%
**PC36:3**	6%	9%	7%	5%	7%	8%	9%	10%	9%	11%	16%	10%	6%	7%	6%	9%	13%	12%	7%	7%
**PC36:4**	5%	12%	8%	5%	9%	9%	10%	11%	23%	16%	23%	22%	3%	2%	2%	10%	17%	17%	2%	2%

**PC36:5**	1%	2%	1%	1%	1%	1%			5%	2%	3%	5%				1%	2%	2%		
**PCO-34:1**							1%	1%					1%	1%	1%				1%	1%
**PE**																				
**Lyso-PE18:1**	14%	5%	19%	16%	8%	15%	5%	1%	6%	3%	4%	6%	4%	5%	6%	4%	6%	8%	2%	7%
**LYsoPE18:2**	2%	1%	2%	1%	1%	2%	1%		2%	1%	1%	1%			1%	1%	1%	1%		1%
**PE34:1**	7%	6%	6%	7%	6%	5%	5%	5%	4%	6%	5%	5%	6%	5%	7%	8%	7%	7%	5%	6%
**PE34:2**	4%	6%	4%	1%	6%	5%	4%	4%	18%	11%	17%	14%	3%	2%	3%	8%	15%	15%	2%	2%
**PE36:1**	5%	5%	5%	6%	5%	4%	4%	5%	2%	4%	1%	2%	4%	5%	5%	4%	2%	2%	5%	5%
**PE36:2**	22%	16%	16%	22%	22%	14%	19%	20%	10%	19%	8%	11%	21%	21%	22%	22%	12%	10%	24%	21%
**PE36:3**	7%	8%	6%	9%	9%	7%	7%	8%	8%	10%	11%	9%	9%	8%	9%	10%	11%	11%	8%	7%
**PE36:4**	2%	3%	3%	2%	3%	3%	2%	3%	16%	8%	13%	13%	1%	1%	1%	4%	11%	11%	1%	1%
**PE38:4**	36%	48%	39%	35%	37%	43%	50%	50%	32%	34%	37%	37%	47%	48%	42%	36%	33%	33%	52%	46%
**PE40:6**	1%	2%			2%	2%	3%	3%	2%	2%	2%	1%	3%	3%	2%	2%	2%	2%		3%
**PEO-34:2**					1%		1%	1%					1%	1%	1%				1%	1%
**SM**																				
**SMd32:0**	3%	3%	3%	3%	3%	2%			3%	2%	3%	3%				3%	3%	3%		
**SMd32:1**	6%	7%	7%	6%	6%	7%	5%	6%	6%	6%	7%	7%	6%	5%	5%	6%	6%	6%	6%	5%
**SMd33:1/SMt32:2**	4%	5%	4%	4%	4%	4%	2%	2%	4%	4%	5%	4%	2%	2%	2%	4%	4%	4%	2%	2%
**SMd34:0**	3%	3%	3%	3%	4%	2%	1%	1%	3%	3%	3%	3%	1%	1%	1%	4%	3%	3%	1%	1%
**SMd34:1**	28%	25%	26%	26%	24%	27%	30%	31%	24%	26%	25%	28%	31%	32%	32%	25%	27%	24%	33%	31%
**SMd34:2**	1%	1%	1%	1%	1%	1%		1%	1%	1%	1%	1%				1%	1%	1%		1%
**SMd36:1**	1%	2%	2%	2%	2%	1%	9%	11%	2%	2%	2%	1%	8%	9%	7%	1%	1%	1%	9%	8%
**SMd36:2**							1%	1%					1%	1%	1%				1%	1%
**SMd38:0**	3%	4%	4%	3%	3%	3%			4%	3%	3%	4%				3%	3%	3%		
**SMd38:1**							1%	2%					1%	1%	1%				2%	2%
**SMd39:0**	4%	4%	4%	3%	4%	3%			4%	3%	4%	4%				4%	4%	4%		
**SMd39:1**	12%	14%	14%	13%	14%	13%	3%	2%	16%	11%	13%	13%	3%	2%	2%	14%	15%	14%	2%	2%
**SMd39:2**	1%	1%	2%	1%	1%	2%			1%	1%	2%	1%				1%	1%	1%		

**SMd40:0**	2%	2%	2%	2%	2%	2%			2%	2%	2%	2%				2%	2%	2%		
**SMd40:1_1**	6%	7%	5%	9%	8%	7%	14%	14%	6%	10%	5%	7%	18%	17%	17%	6%	5%	8%	18%	16%
**SMd40:1_2**	5%	6%	5%	4%	5%	5%	2%	2%	6%	5%	5%	5%	1%	1%	1%	6%	5%	5%	1%	3%
**SMd40:2**	2%	1%	3%	2%	2%	3%	1%	1%	3%	3%	3%	2%	1%	1%	1%	3%	2%	1%	1%	1%
**SMd41:1**	14%	12%	13%	13%	13%	12%	22%	19%	14%	14%	11%	13%	20%	21%	22%	14%	14%	15%	17%	22%
**SMd41:2**	1%	1%	1%	2%	1%	2%	2%	1%		1%	2%		1%	1%	1%			1%	1%	1%
**SMd42:2**	2%	2%	2%	2%	2%	2%	4%	4%	2%	2%	3%	2%	3%	3%	4%	2%	2%	2%	3%	3%
										**TG**										
**TG30:0**	1%	1%		1%	1%		1%		1%	1%	1%	1%	1%	1%		1%	1%			1%
**TG32:0**	3%	3%		3%	3%		1%	1%	3%	3%	3%	2%	1%	1%	1%	3%	2%		1%	1%
**TG34:0**	4%	4%		4%	4%		1%	1%	3%	4%	4%	3%	2%	1%	2%	3%	4%		1%	2%
**TG34:1**							1%	1%					1%	1%	1%				1%	1%
**TG36:0**	5%	5%		5%	5%		2%	2%	4%	4%	4%	2%	2%	2%	3%	4%	4%		2%	3%
**TG36:1**							1%	1%				1%	1%	1%	1%				1%	1%
**TG38:0**	3%	4%		3%	4%				3%	3%	3%	1%				3%	3%			
**TG38:1**	1%	1%		1%	1%		3%	3%		1%		2%	3%	3%	3%	1%			3%	3%
**TG38:2**							1%	1%					1%	1%	1%				1%	1%
**TG40:0**	3%	3%		3%	3%		1%	2%	2%	2%	2%	1%	2%	2%	1%	2%	2%			3%
**TG40:1**							1%	1%				1%								
**TG40:2**							1%	1%				1%	1%	1%	1%				1%	1%
**TG42:0**	2%	2%		2%	2%		2%	2%	1%	1%	1%	1%	3%	3%	3%	1%	1%		2%	2%
**TG42:1**	1%	1%		1%	1%		2%	1%	1%	1%	1%	1%	1%	2%	1%	1%	1%		1%	1%
**TG42:2**							1%	1%						1%					1%	1%
**TG44:0**	1%	1%		1%	1%		1%	1%	1%	1%	1%	1%	1%	1%	1%	1%	1%		1%	1%
**TG44:1**	1%	1%		1%	1%		3%	3%		1%		1%	3%	3%	2%	1%	1%		3%	3%
**TG44:2**							1%	1%					1%	1%	1%				1%	1%
**TG46:1**	1%	1%		1%	1%		1%	1%		1%		1%	2%	2%	2%	1%	1%		1%	2%
**TG46:2**							1%	1%					1%	1%	1%				1%	1%
**TG48:1**	1%	1%	1%	1%	1%	1%	1%	1%	1%	1%	1%	1%	2%	2%	2%	1%		1%	2%	2%
**TG48:2**		1%		1%	1%		1%	1%		1%	1%	1%	1%	1%	1%				1%	1%
**TG50:1**	8%	7%	9%	8%	8%	9%	2%	2%	12%	10%	12%	7%	11%	11%	11%	1%	1%	13%	10%	11%

**TG50:2**	3%	3%	3%	3%	3%	3%	1%	1%	4%	5%	4%	3%	4%	4%	4%	1%	1%	5%	4%	4%
**TG50:3**													1%	1%	1%					1%
**TG52:1**	2%	2%	2%	2%	2%	2%	1%	1%	3%	3%	2%	2%	3%	3%	3%	1%	1%	3%	3%	3%
**TG52:2**	10%	9%	14%	10%	10%	14%	7%	6%	13%	11%	13%	10%	12%	13%	13%	7%	7%	14%	13%	13%
**TG52:3**	5%	5%	6%	5%	5%	6%	3%	3%	7%	7%	6%	5%	6%	5%	6%	3%	3%	8%	5%	5%
**TG52:4**	2%	3%	3%	2%	2%	3%	2%	2%	4%	4%	4%	3%	2%	2%	2%	3%	3%	4%	2%	2%
**TG52:5**									1%	1%	1%	1%								
**TG54:1**													1%	1%	1%				1%	1%
**TG54:2**	2%	2%	4%	2%	2%	4%	4%	4%	3%	3%	3%	3%	3%	3%	3%	4%	4%	3%	3%	3%
**TG54:3**	15%	16%	27%	15%	14%	26%	23%	23%	14%	12%	14%	15%	8%	8%	8%	25%	28%	13%	13%	8%
**TG54:4**	8%	9%	11%	8%	8%	11%	8%	8%	4%	5%	4%	8%	6%	5%	6%	11%	9%	11%	6%	5%
**TG54:5**	7%	7%	8%	8%	7%	8%	7%	7%	4%	5%	5%	7%	5%	4%	5%	10%	10%	11%	5%	4%
**TG54:6**	4%	4%	4%	4%	4%	4%	4%	4%	5%	5%	5%	5%	3%	3%	3%	6%	6%	6%	3%	3%
**TG54:7**	1%	1%	1%	1%	1%	1%	1%	1%	1%	1%	1%	2%				1%	1%	1%		
**TG56:2**			1%			1%										1%				
**TG56:3**	1%	1%	1%	1%	1%	1%	1%	1%				1%				1%	1%	1%		
**TG58:2**			1%			1%	1%	1%								1%	1%			

DG, diacylglycerol; MG, monoacylglycerol; PC, glycerophosphocholine; PC-O, ether analogue of glycerophosphocholine; PE, glycerophosphoethanoloamine; PE-O, ether anlogue of glycerophoshoethanoloamine; PS, glycerophosphoserine, PI, glycerophosphoinositol; SM, sphingomyelin; TG, triacylglycerol; TGO, ether analogue of triacyglycerol.

Table S8. The complete list of statistically significantly different between the colostrum and further lactation stage samples (ANOVA unequal variance test, p<0.01, multiple testing correction: Benjamini-Hochberg, colostrum versus HM 0-6 mo., colostrum versus HM 6-12 mo., colostrum versus HM > 12 mo.

Compound	p (Corr) (HM colostrum vs HM 0-6 mo. vs HM 6-12 mo. vs HM> 12 mo.)	p HM 0-6 mo. vs colostrum	p (HM6-12 mo. vs colostrum)	p (HM> 12 mo. vs colostrum)	Average peak area fold change HM 0-6 mo. (n=10) vs colostrum (n=11)	Average peak area fold change HM 6-12 mo. (n=8) vs colostrum (n=11)	Average peak area fold change HM > 12 mo (n=16). vs colostrum (n=11)
**DG24:0**	7.91E-08	1.46E-04	1.46E-04	1.46E-04	4.0[a]	3.2	4.3
**DG32:1**	2.73E-07	1.71E-04	1.65E-04	1.46E-04	2.8	3.2	3.2
**DG36:1**	1.90E-05	7.37E-03	1.31E-03	1.49E-04	2.1	2.7	3.0
**DG36:2**	3.51E-05	3.49E-04	1.15E-03	2.09E-04	2.8	2.8	2.7
**DG36:3**	9.96E-10	1.46E-04	9.69E-03	1.54E-04	4.4	2.8	3.5
**DG36:4**	1.32E-11	1.46E-04	1.46E-04	1.46E-04	9.0	6.7	6.7
**LysoPC18:3**	5.21E-08	1.81E-04	1.19E-03	1.46E-04	2.8	2.6	3.9
**LysoPCO-18:0/LysoPEO-21:0**	4.76E-03	1.48E-04	1.46E-04	5.37E-04	-3.3	-2.6	-1.0
**LysoPE18:2**	9.17E-14	1.46E-04	1.46E-04	1.46E-04	3.5	3.4	4.5
**PC30:0**	3.41E-07	1.46E-04	1.58E-04	8.54E-04	-3.4	-3.1	-2.1
**PC31:0**	9.56E-11	1.46E-04	1.46E-04	1.46E-04	-3.2	-3.7	-2.9
**PC32:0**	3.56E-10	1.46E-04	1.46E-04	1.46E-04	-2.7	-3.9	-2.4
**PC33:0**	9.37E-06	3.20E-04	1.72E-04	2.44E-04	-2.7	-3.3	-2.5
**PC34:0**	2.74E-06	9.86E-04	1.46E-04	6.24E-04	-2.0	-3.1	-1.9
**PCO-34:1**	1.67E-14	1.46E-04	1.46E-04	1.46E-04	-3.6	-5.3	-3.0
**PEO-36:5**	3.34E-08	1.61E-04	1.46E-04	6.09E-03	-2.8	-4.7	-1.9
**PI36:1**	1.09E-12	1.46E-04	1.27E-03	1.46E-04	2.6	2.0	4.1
**PI38:4**	3.32E-12	1.46E-04	1.46E-04	1.46E-04	-3.4	-6.7	-3.6
**PS36:1**	1.39E-07	1.66E-04	1.46E-04	2.26E-04	-3.1	-4.9	-2.6
**SMd33:1/SMt32:2**	1.72E-09	1.46E-04	1.46E-04	1.68E-04	-2.5	-3.4	-1.9
**SMd34:1**	3.21E-08	1.56E-04	1.46E-04	4.87E-03	-2.3	-3.4	-1.7

**SMd34:2**	1.83E-08	1.46E-04	1.46E-04	1.59E-04	-2.9	-3.4	-2.2
**SMd36:0**	4.72E-13	1.49E-04	5.21E-04	1.46E-04	3.1	2.7	6.6
**SMd42:0**	1.15E-13	1.52E-04	1.96E-04	1.46E-04	3.6	3.6	9.6
**SMt34:1**	1.96E-20	1.46E-04	1.46E-04	1.46E-04	-11.1	-21.6	-6.2
**SMt42:1**	4.86E-13	1.46E-04	1.46E-04	1.47E-04	-3.8	-5.2	-2.3
**TG34:1**	2.45E-15	1.46E-04	1.46E-04	1.46E-04	10.2	7.4	13.8
**TG36:1**	6.92E-14	1.46E-04	1.59E-04	1.46E-04	7.5	4.5	11.6
**TG37:0**	4.49E-05	3.67E-04	4.96E-03	1.46E-04	3.6	3.0	6.8
**TG38:1**	8.94E-17	1.46E-04	1.46E-04	1.46E-04	7.4	4.9	9.6
**TG38:2**	2.96E-16	1.46E-04	1.46E-04	1.46E-04	9.8	5.2	10.6
**TG38:3**	3.10E-09	1.46E-04	1.50E-04	1.46E-04	9.4	3.9	12.3
**TG39:0**	3.56E-10	1.46E-04	8.81E-04	1.46E-04	9.0	4.9	12.8
**TG39:1**	3.99E-15	1.46E-04	1.46E-04	1.46E-04	9.8	5.8	11.0
**TG40:0**	8.56E-10	1.51E-04	4.65E-03	1.46E-04	4.2	2.9	7.1
**TG40:1**	1.51E-14	1.46E-04	1.46E-04	1.46E-04	7.3	5.1	7.6
**TG40:2**	1.67E-14	1.46E-04	1.46E-04	1.46E-04	8.6	5.8	11.1
**TG40:3**	5.19E-14	1.46E-04	1.46E-04	1.46E-04	9.5	5.3	9.7
**TG41:0**	1.33E-10	1.47E-04	3.68E-03	1.46E-04	4.8	3.0	8.2
**TG41:1**	8.27E-11	1.46E-04	1.46E-04	1.46E-04	6.8	4.3	7.8
**TG42:0**	1.03E-09	2.41E-04	7.38E-03	1.46E-04	3.1	2.5	5.9
**TG42:1**	1.76E-13	1.46E-04	1.48E-04	1.46E-04	5.4	3.7	6.7
**TG42:2**	2.57E-13	1.46E-04	1.65E-04	1.46E-04	8.9	4.4	9.4
**TG42:3**	1.50E-13	1.46E-04	1.46E-04	1.46E-04	12.1	6.8	11.9
**TG43:0**	2.55E-09	1.50E-04	2.07E-03	1.46E-04	3.3	2.6	4.8
**TG43:1**	7.80E-12	1.46E-04	1.57E-04	1.46E-04	6.1	4.0	6.9
**TG44:1**	2.97E-12	1.46E-04	1.96E-04	1.46E-04	3.6	2.7	4.9
**TG44:2**	1.22E-13	1.46E-04	1.66E-04	1.46E-04	5.9	3.3	6.7
**TG44:3**	7.18E-14	1.46E-04	1.46E-04	1.46E-04	9.1	4.7	9.6
**TG46:2**	3.35E-12	1.46E-04	4.55E-04	1.46E-04	3.6	2.3	4.3
**TG46:3**	2.06E-14	1.46E-04	1.48E-04	1.46E-04	6.9	3.5	6.0
**TG46:4**	1.22E-13	1.46E-04	1.46E-04	1.46E-04	14.3	6.9	11.0

**TG47:3**	6.79E-10	1.46E-04	1.46E-04	1.46E-04	4.9	2.7	5.0
**TG48:2**	7.59E-09	1.55E-04	4.33E-03	1.46E-04	2.2	1.8	2.8
**TG48:3**	5.66E-12	1.46E-04	2.98E-04	1.46E-04	3.9	2.5	4.2
**TG48:4**	2.73E-12	1.46E-04	2.07E-04	1.46E-04	5.4	3.0	5.2
**TG48:5**	2.40E-09	1.46E-04	1.88E-04	1.46E-04	8.6	6.7	11.2
**TG58:2**	8.10E-08	1.46E-04	1.49E-04	1.47E-04	-4.8	-4.9	-3.9
**TG58:3**	3.23E-04	7.73E-04	4.50E-03	1.37E-03	-2.3	-2.3	-2.1
**TG58:4**	6.90E-05	9.31E-04	3.91E-04	5.37E-04	-2.6	-3.2	-2.5
**TG58:6**	8.39E-08	1.57E-04	1.46E-04	1.89E-04	-2.3	-3.3	-2.0
**TG60:2**	1.43E-08	1.46E-04	4.97E-04	1.46E-04	-5.6	-3.8	-5.8
**TG60:4**	3.71E-05	8.63E-03	3.53E-04	1.46E-04	-3.7	-6.8	-4.9
**TG62:3**	9.12E-18	1.46E-04	1.46E-04	1.46E-04	-25.3	-39.3	-20.1
**TG62:4**	3.99E-21	1.46E-04	1.46E-04	1.46E-04	-17.1	-28.1	-23.1
**TGO-50:1**	6.62E-10	1.46E-04	1.46E-04	4.85E-04	-4.7	-7.9	-3.8
**TGO-52:2**	1.37E-11	1.46E-04	1.46E-04	1.46E-04	-3.4	-4.9	-3.1
**TGO-54:2**	1.42E-05	1.46E-04	1.46E-04	8.81E-04	-2.7	-3.1	-2.4
**DG34:2**	3.13E-05	1.64E-04		3.32E-04	3.1	2.2	2.5
**LysoPC16:0**	3.67E-06	1.35E-03	1.52E-04		-1.7	-2.3	-1.2
**Lyso-PC18:2**	4.31E-07	7.79E-04		1.46E-04	2.0	1.6	2.7
**PC29:0**	6.57E-04	3.22E-03		1.63E-04	-2.6	-2.3	-1.6
**PC32:1**	1.96E-05	2.49E-04	1.87E-04		-2.4	-2.7	-1.6
**PC33:1**	2.24E-03	7.78E-03	3.49E-03		-2.0	-2.3	-1.5
**PC34:1**	3.09E-05	6.50E-03	1.72E-04		-1.8	-2.6	-1.2
**PC35:1**	9.10E-04	1.03E-03	8.73E-03		-3.8	-3.4	-1.8
**PC36:5**	6.40E-04	2.58E-04		1.46E-04	2.6	1.6	3.5
**PC38:6**	2.34E-04	1.28E-03	7.88E-04		-2.6	-3.1	-1.5
**PE36:2**	3.15E-05	3.22E-03		1.51E-04	3.3	2.1	4.8
**PE36:3**	4.31E-07	2.45E-04		1.46E-04	3.1	1.9	4.0
**PE36:4**	6.00E-08	1.57E-04		1.46E-04	4.3	2.6	5.8
**PEO-38:5**	4.59E-06	1.22E-03	1.47E-04		-2.2	-3.5	-1.5
**PEO-38:7**	4.39E-07	9.35E-04	1.46E-04		-3.0	-6.9	-2.1

**PEO-40:7**	3.15E-05	7.81E-03	1.64E-04		-2.2	-3.8	-1.4
**PI36:2**	1.77E-08	2.15E-04		1.46E-04	2.6	1.5	3.6
**PI36:3**	5.40E-05	1.46E-04		1.46E-04	1.4	-1.4	1.7
**PI38:3**	2.43E-05		1.51E-04	4.93E-03	-1.5	-3.8	-2.0
**SMd30:1**	1.89E-10	1.47E-04		1.46E-04	3.0	1.6	3.9
**SMd32:0**	3.03E-08	1.49E-03		1.46E-04	2.4	1.6	4.2
**SMd36:1**	8.33E-09	5.88E-04		1.46E-04	2.1	1.6	3.3
**SMd38:1**	7.32E-09	1.10E-03		1.46E-04	1.9	1.3	3.0
**SMd40:1_1**	3.31E-11	1.50E-04		1.46E-04	2.5	1.8	3.9
**SMd42:3**	7.21E-06	1.68E-04	2.43E-04		-2.8	-2.8	-1.5
**TG30:0**	4.47E-03	1.52E-04		1.46E-04	2.4	1.8	3.6
**TG32:0**	9.22E-07	1.20E-03		1.46E-04	3.3	2.0	5.4
**TG34:0**	3.23E-07	1.53E-04		1.46E-04	4.9	2.5	5.5
**TG36:0**	1.49E-07	2.06E-04		1.46E-04	4.9	3.1	7.6
**TG38:0**	5.05E-06	2.04E-03		1.46E-04	3.9	2.9	6.6
**TG44:0**	7.20E-09	2.14E-03		1.46E-04	2.3	1.8	4.3
**TG46:1**	6.17E-09	3.30E-04		1.46E-04	2.1	1.7	3.2
**TG50:3**	4.98E-06	8.23E-04		1.48E-04	2.0	1.3	2.3
**TG50:4**	4.31E-07	1.55E-04		1.46E-04	2.7	1.6	2.8
**TG52:3**	5.54E-04	4.92E-03		5.45E-03	1.8	1.0	1.6
**TG52:4**	4.49E-05	2.60E-04		1.61E-03	2.2	1.1	1.8
**TG52:5**	2.53E-04	3.51E-04		9.69E-03	2.5	1.2	1.9
**TG52:6**	5.05E-06	3.51E-04		1.47E-04	3.0	1.7	3.4
**TG52:7**	8.50E-07	1.91E-04		1.46E-04	2.9	1.5	3.2
**TG53:3**	6.10E-04	5.82E-04		2.36E-03	2.1	1.5	1.8
**TG54:5**	4.24E-05	1.55E-04		1.89E-03	2.7	1.5	1.9
**TG54:6**	6.01E-06	1.47E-04		3.47E-04	3.8	2.0	2.6
**TG54:7**	8.88E-06	1.47E-04		4.88E-04	5.4	2.9	3.2
**TG58:5**	7.96E-05		1.23E-03	1.48E-04	-2.4	-4.4	-4.1
**TGO-52:1**	5.05E-06	1.46E-04	4.37E-04		-4.9	-4.5	-2.3
**TGO-54:3**	2.85E-04		1.46E-04	9.32E-04	-2.4	-3.6	-2.5

**Lyso-PC14:0**	1.25E-03	7.98E-03			-1.9	-1.6	1.0
**Lyso-PC16:1**	2.71E-03		1.13E-03		-1.5	-1.9	-1.3
**Lyso-PC18:0**	1.79E-05			1.78E-03	-1.0	-1.3	1.9
**LysoPC22:6**	8.22E-03			6.54E-03	1.2	1.1	1.8
**Lyso-PE18:0**	1.25E-05			1.15E-03	-1.3	-1.2	2.6
**PC32:2**	2.68E-03			1.42E-03	1.4	1.4	2.5
**PC36:1**	3.86E-06		1.57E-04		-1.7	-3.1	-1.0
**PC36:2**	3.04E-03			9.68E-03	1.4	-1.1	1.7
**PC36:3**	2.71E-03			2.52E-03	1.6	1.1	2.0
**PC36:4**	5.62E-04			3.15E-04	1.9	1.5	3.6
**PC38:3**	2.89E-03		4.49E-03		-1.1	-2.5	-1.0
**PC38:4**	9.99E-03		4.23E-03		-1.4	-2.7	-1.3
**PE34:2**	9.36E-05			1.90E-04	2.0	1.3	3.5
**PE38:4**	2.60E-03			9.96E-03	1.4	-1.1	1.8
**PEO-34:2**	2.18E-05		1.65E-04		-1.9	-3.3	-1.2
**PS36:2**	6.33E-03			8.95E-03	1.4	1.0	1.8
**SMd32:1**	2.06E-05			1.14E-03	1.2	-1.3	1.9
**SMd34:0**	2.43E-04		1.65E-03		-1.3	-2.7	1.1
**SMd36:2**	3.47E-03		8.34E-03		-1.4	-1.8	-1.0
**SMd41:1**	1.46E-08			1.46E-04	1.5	1.2	2.6
**SMd44:1**	1.42E-03		1.46E-04		-2.5	-2.7	-1.3
**TG48:1**	1.03E-06			1.46E-04	1.4	1.2	2.4
**TG50:2**	4.37E-04			9.30E-04	1.4	-1.0	1.7
**TG50:6**	4.99E-06			1.60E-04	2.2	-1.1	3.4
**TG52:1**	5.12E-04			8.00E-04	1.3	1.0	2.0
**TG53:1**	4.32E-04			2.56E-04	1.6	1.3	2.3
**TG54:4**	1.69E-03	1.01E-03			2.0	1.3	1.6
**TG56:5**	8.05E-03		8.06E-03		-1.5	-2.2	-1.8
**TG56:6_1**	2.28E-05		1.50E-04		-1.2	-2.7	-1.5
**TG56:7_1**	1.62E-03	5.52E-04			2.8	1.5	1.5
**TG56:7_2**	8.22E-04		3.07E-04		-1.5	-2.7	-1.4

**TG56:8**	3.21E-03		6.36E-03		1.1	-2.4	-1.1
**TG58:10**	1.42E-03	8.34E-03			2.4	-1.3	1.6
**TG58:7_1**	2.68E-03		1.11E-03		-1.2	-2.1	-1.4
**TG58:7_2**	4.59E-04		4.54E-04		-1.3	-2.2	-1.1
**TG58:8_1**	7.29E-03		7.90E-03		-1.0	-2.0	-1.1
**PC30:1**	1.67E-03				-1.9	-2.2	-1.3
**PC34:2**	9.51E-03				-1.0	-1.3	1.4
**PE36:1**	1.23E-03				1.1	-1.6	1.6
**PS40:6**	4.75E-03				-2.6	-2.9	-1.5
**SMd40:2**	6.37E-04				1.0	-1.6	1.6
**SMd42:2**	5.54E-04				-1.3	-1.8	1.2
**TG50:1**	1.12E-03				1.0	-1.4	1.5
**TG54:1**	6.52E-03				1.1	-1.0	1.9
**TG54:7_2**	2.33E-03				1.5	-1.3	1.8
**TG56:9**	2.33E-03				1.6	-2.1	1.1
**TG58:9**	3.96E-03				1.4	-1.8	1.1

[a]Negative fold change value value means higher peak area in colostrum samples than in mature HM samples, positive fold change value means higher peak area in mature HM samples than in the colostrum samples; ns, not statistically significant; DG, diacylglycerol; MG, monoacylglycerol; PC, glycerophosphocholine; PC-O, ether analogue of glycerophosphocholine; PE, glycerophosphoethanoloamine; PE-O, ether analogue of glycerophoshoethanoloamine; PS, glycerophosphoserine, PI, glycerophosphoinositol; SM, sphingomyelin; TG, triacylglycerol; TGO, ether analogue of triacyglycerol.

Table S9. The complete list of lipids statistically significantly different between the colostrum and further lactation stage HM samples (Mann-Whitney test, p<0.01, multiple testing correction: Benjamini-Hochberg colostrum vs mature HM (lactation stage other than colostrum).

Compound	p (HM colostrum vs mature HM)	p (Corr) (HM colostrum vs mature HM)	Average peak area fold change colostrum (n=11) vs mature HM (n=34)
**DG24:0**	3.1E-07	1.3E-06	-4.0
**DG32:1**	2.6E-06	8.2E-06	-3.1
**DG34:1**	3.7E-03	6.4E-03	-1.6
**DG34:2**	1.4E-05	3.9E-05	-2.6
**DG36:1**	2.8E-06	8.9E-06	-2.6
**DG36:2**	2.0E-06	6.8E-06	-2.7
**DG36:3**	3.4E-06	1.1E-05	-3.6
**DG36:4**	1.2E-08	7.8E-08	-7.3
**LysoPC16:0**	7.2E-04	1.4E-03	1.5
**Lyso-PC16:1**	3.2E-03	5.5E-03	1.5
**Lyso-PC18:2**	8.1E-06	2.3E-05	-2.2
**lyso-PC18:3**	1.8E-06	6.0E-06	-3.2
**LysoPE18:2**	1.5E-10	4.1E-09	-3.9
**PC24:0**	1.4E-03	2.5E-03	-1.1
**PC29:0**	3.5E-04	7.4E-04	2.0
**PC30:0**	1.6E-06	5.5E-06	2.7
**PC30:1**	2.3E-03	4.1E-03	1.6
**PC31:0**	9.4E-10	1.2E-08	3.1
**PC32:0**	2.6E-09	2.3E-08	2.8
**PC32:1**	5.0E-05	1.2E-04	2.0
**PC33:0**	3.0E-07	1.3E-06	2.7
**PC33:1**	5.2E-04	1.1E-03	1.8
**PC34:0**	1.3E-06	4.6E-06	2.2
**PC34:1**	8.0E-04	1.5E-03	1.6
**PC35:1**	9.2E-04	1.7E-03	2.6
**PC36:4**	1.9E-03	3.5E-03	-2.5
**PC38:6**	1.1E-03	2.0E-03	2.1
**PCO-34:1**	1.2E-10	4.1E-09	3.6
**PE34:2**	1.2E-03	2.2E-03	-2.4
**PE36:0**	2.2E-07	9.8E-07	-1.0
**PE36:2**	2.0E-05	5.0E-05	-3.6
**PE36:3**	1.9E-05	4.9E-05	-3.2
**PE36:4**	7.4E-06	2.1E-05	-4.5
**PEO-34:2**	2.5E-03	4.3E-03	1.7
**PEO-36:5**	7.3E-07	2.8E-06	2.6
**PEO-38:5**	1.8E-04	4.1E-04	2.0
**PEO-38:7**	3.7E-06	1.1E-05	3.0
**PEO-40:7**	5.6E-04	1.1E-03	2.0
**PI36:1**	7.0E-09	4.9E-08	-3.1
**PI36:2**	8.1E-06	2.3E-05	-2.7
**PI38:3**	1.1E-04	2.5E-04	2.1

**PI38:4**	2.9E-09	2.5E-08	4.0
**PS36:1**	8.4E-08	4.3E-07	3.1
**PS40:6**	6.9E-04	1.4E-03	2.1
**SMd30:1**	1.6E-07	7.3E-07	-3.0
**SMd32:0**	3.9E-06	1.2E-05	-2.9
**SMd33:1/SMt32:2**	2.3E-07	1.0E-06	2.4
**SMd34:1**	3.6E-06	1.1E-05	2.1
**SMd34:2**	8.9E-08	4.3E-07	2.6
**SMd36:0**	2.6E-09	2.3E-08	-4.4
**SMd36:1**	4.9E-07	2.0E-06	-2.5
**SMd38:0**	2.0E-03	3.7E-03	-7.5
**SMd38:1**	1.1E-06	4.1E-06	-2.2
**SMd39:0**	1.3E-07	6.0E-07	1.1
**SMd40:1_1**	4.9E-09	3.7E-08	-2.9
**SMd40:1_2**	9.7E-05	2.2E-04	1.9
**SMd41:1**	4.0E-05	9.8E-05	-1.9
**SMd42:0**	1.6E-10	4.1E-09	-5.8
**SMd42:3**	2.0E-05	5.0E-05	2.1
**SMd43:2**	7.4E-05	1.7E-04	16.0
**SMt34:1**	5.1E-10	8.0E-09	9.6
**SMt42:1**	7.8E-09	5.4E-08	3.2
**TG32:0**	9.3E-07	3.5E-06	-3.8
**TG33:1**	7.2E-04	1.4E-03	-3.0
**TG34:0**	1.1E-06	4.0E-06	-4.5
**TG34:1**	5.8E-08	3.1E-07	-11.0
**TG36:0**	6.2E-06	1.8E-05	-5.5
**TG36:1**	6.9E-10	9.6E-09	-8.3
**TG37:0**	3.3E-04	6.9E-04	-4.7
**TG38:0**	1.7E-05	4.3E-05	-4.7
**TG38:1**	4.1E-11	4.1E-09	-7.7
**TG38:2**	6.0E-11	4.1E-09	-8.9
**TG38:3**	2.1E-05	5.4E-05	-8.9
**TG39:0**	8.4E-08	4.3E-07	-9.4
**TG39:1**	2.0E-10	4.6E-09	-9.3
**TG40:0**	6.0E-07	2.3E-06	-5.0
**TG40:1**	1.1E-10	4.1E-09	-6.9
**TG40:2**	1.2E-10	4.1E-09	-8.9
**TG40:3**	2.1E-10	4.6E-09	-8.5
**TG41:0**	2.8E-08	1.6E-07	-5.6
**TG41:1**	4.7E-07	1.9E-06	-6.6
**TG42:0**	4.4E-07	1.9E-06	-4.0
**TG42:1**	1.3E-09	1.4E-08	-5.5
**TG42:2**	1.4E-09	1.4E-08	-7.9
**TG42:3**	5.8E-10	8.5E-09	-10.6
**TG43:0**	2.4E-08	1.4E-07	-3.7
**TG43:1**	1.8E-09	1.8E-08	-5.9
**TG44:0**	8.9E-08	4.3E-07	-2.9
**TG44:1**	4.9E-09	3.7E-08	-3.9

**TG44:2**	4.8E-10	8.0E-09	-5.6
**TG44:3**	2.6E-08	1.5E-07	-8.1
**TG46:1**	1.3E-06	4.4E-06	-2.5
**TG46:2**	1.5E-09	1.5E-08	-3.6
**TG46:3**	9.4E-10	1.2E-08	-5.6
**TG46:4**	8.3E-11	4.1E-09	-10.8
**TG47:3**	9.3E-06	2.6E-05	-4.4
**TG48:1**	1.9E-04	4.2E-04	-1.7
**TG48:2**	8.0E-08	4.2E-07	-2.4
**TG48:3**	3.1E-09	2.6E-08	-3.6
**TG48:4**	4.4E-09	3.5E-08	-4.7
**TG48:5**	2.4E-08	1.4E-07	-9.3
**TG50:3**	1.5E-04	3.3E-04	-1.9
**TG50:4**	8.9E-06	2.5E-05	-2.5
**TG50:5**	4.5E-04	9.4E-04	-3.1
**TG50:6**	2.0E-03	3.7E-03	-2.3
**TG51:3**	3.5E-04	7.4E-04	2.2
**TG52:1**	4.6E-03	7.7E-03	-1.5
**TG52:4**	1.5E-03	2.8E-03	-1.7
**TG52:5**	4.5E-03	7.5E-03	-1.9
**TG52:6**	2.3E-05	5.8E-05	-2.8
**TG52:7**	4.2E-05	1.0E-04	-2.7
**TG53:1**	3.3E-05	8.0E-05	-1.9
**TG53:3**	2.5E-04	5.4E-04	-1.8
**TG54:4**	6.9E-04	1.4E-03	-1.6
**TG54:5**	8.1E-05	1.9E-04	-2.0
**TG54:6**	6.2E-06	1.8E-05	-2.7
**TG54:7**	2.5E-06	8.1E-06	-3.7
**TG56:5**	4.1E-03	6.9E-03	1.8
**TG56:6_1**	2.3E-03	4.0E-03	1.6
**TG58:2**	1.2E-09	1.4E-08	4.4
**TG58:3**	3.0E-05	7.4E-05	2.2
**TG58:4**	1.0E-05	2.7E-05	2.7
**TG58:5**	1.2E-04	2.7E-04	3.5
**TG58:6**	6.0E-07	2.3E-06	2.4
**TG60:2**	1.1E-08	7.2E-08	5.2
**TG60:3**	9.9E-06	2.7E-05	-31.9
**TG60:4**	4.7E-04	9.6E-04	4.8
**TG62:3**	1.1E-06	4.1E-06	24.5
**TG62:4**	1.0E-08	6.9E-08	21.9
**TG62:5**	6.7E-04	1.3E-03	18.2
**TG62:6**	2.5E-10	4.9E-09	9.3
**TG62:8**	4.6E-03	7.7E-03	19.9
**TG64:4**	3.8E-08	2.1E-07	14.4
**TG64:6**	1.6E-07	7.3E-07	12.8
**TG64:8**	6.8E-09	4.9E-08	23.6
**TGO-50:1**	1.3E-03	2.5E-03	4.5
**TGO-52:1**	3.1E-03	5.3E-03	3.2

**TGO-52:2**	4.8E-10	8.0E-09	3.5
**TGO-54:2**	3.1E-04	6.7E-04	2.6

[a]Positive fold change value value means higher peak area in colostrum samples than in mature HM samples, negative fold change value means higher peak area in mature HM samples than in the colostrum samples; DG, diacylglycerol; PC, glycerophosphocholine; PC-O, ether analogue of glycerophosphocholine; PE, glycerophosphoethanoloamine; PE-O, ether anlogue of glycerophoshoethanoloamine; PS, glycerophosphoserine, PI, glycerophosphoinositol; SM, sphingomyelin; TG, triacylglycerol; TG-O, ether analogue of triacyglycerol; ns – not statistically significant change.

Table S10. The complete list of statistically significantly (p<0.01) different lipids between the samples of caprine whole milk-based FM and FM supplemented with soy lecithin accordingly to the Mann-Whitney test unpaired (multiple testing correction: Benjamini-Hochberg).

Compound	p (Corr) caprine whole milk vs soy lecithin	Average peak area fold change caprine whole milk based FM (n=7) vs soy lecithin supplemented FM (n=13)
**TG30:1**	1.2E-05	83.4
**TG40:3**	2.7E-06	57.1
**TG43:1**	2.7E-06	54.9
**TG39:1**	2.7E-06	41.1
**TG41:1**	1.2E-05	41.0
**TG34:2**	2.7E-06	37.5
**TG41:0**	2.7E-06	35.1
**TG34:1**	2.7E-06	31.7
**TG42:3**	1.2E-05	31.5
**TG38:3**	2.7E-06	31.1
**TG39:0**	2.7E-06	29.8
**TG32:1**	9.0E-04	25.8
**TG33:0**	2.2E-04	21.9
**TG35:0**	3.8E-06	17.8
**TG46:3**	2.7E-06	17.6
**TG40:2**	2.7E-06	17.3
**TG43:0**	2.7E-06	17.2
**TG44:2**	2.7E-06	14.4
**TG37:0**	8.1E-06	13.9
**SMd28:1**	1.2E-05	12.7
**TG38:2**	2.7E-06	12.4
**TG36:1**	2.7E-06	12.1
**SMd38:1**	2.2E-05	11.1
**TG44:3**	2.7E-06	9.3
**TG46:2**	2.7E-06	8.6
**TG42:2**	5.4E-06	8.0
**TG38:1**	2.7E-06	7.4
**SMd36:1**	7.0E-06	6.8
**TG44:1**	2.7E-06	6.3
**TG53:1**	2.7E-06	4.2
**SMd36:2**	2.4E-05	3.5

**TG42:1**	1.8E-05	3.5
**TG46:1**	5.4E-06	3.1
**TG42:0**	1.2E-04	3.1
**SMd40:1_1**	1.5E-04	3.0
**TG44:0**	2.2E-04	2.6
**TG48:3**	2.4E-05	2.6
**TG48:4**	2.9E-05	2.4
**TG55:2**	8.1E-06	2.3
**TG48:1**	1.3E-05	2.3
**PEO-34:2**	5.6E-04	2.1
**SMd41:2**	3.6E-03	2.1
**SMd42:2**	1.3E-04	2.1
**SMd36:0**	2.7E-03	2.0
**TG38:0**	4.1E-04	2.0
**PCO-34:1**	6.0E-03	1.9
**SMd41:1**	1.3E-03	1.9
**SMt34:1**	4.7E-03	1.8
**PC34:0**	5.1E-04	1.8
**TG53:2**	1.4E-04	1.7
**TG48:2**	1.2E-03	1.7
**TG50:3**	1.2E-03	1.6
**TG50:4**	1.5E-04	1.5
**TG56:3**	5.6E-03	-1.4
**TG56:4**	4.3E-03	-1.4
**TG54:4**	2.3E-03	-1.4
**TG54:5**	7.5E-04	-1.5
**TG52:4**	1.1E-04	-1.6
**TG56:5**	2.3E-03	-1.6
**TG54:6**	4.1E-04	-1.6
**TG54:3**	6.5E-03	-1.6
**PC24:0**	4.3E-03	-1.6
**TG58:3**	1.9E-03	-1.6
**SMd33:1/SMt32:2**	9.0E-04	-1.8
**PC26:0**	1.3E-03	-1.8
**DG36:2**	3.4E-04	-1.9
**TG60:2**	9.3E-03	-1.9
**DG36:4**	3.1E-04	-2.1
**DG36:3**	5.6E-04	-2.1
**PC32:1**	1.7E-04	-2.1
**LysoPE18:1**	1.1E-03	-2.2
**TG52:5**	8.8E-06	-2.3
**LysoPC18:0**	1.5E-04	-2.4
**LysoPC16:0**	1.1E-03	-2.4
**PC32:2**	1.2E-04	-2.4
**SMd40:1_2**	1.5E-04	-2.6
**TG52:6**	4.7E-05	-2.7
**PC30:0**	2.9E-05	-2.7
**TG54:7**	1.4E-05	-2.9

**LysoPE18:2**	2.0E-04	-2.9
**PC34:3**	4.1E-04	-2.9
**SMd34:0**	2.0E-05	-3.0
**SMd39:2**	8.1E-06	-3.1
**TG54:8**	1.4E-05	-3.1
**LysoPC18:1**	9.0E-04	-3.2
**PC38:4**	6.8E-05	-3.2
**PC30:1**	8.8E-06	-3.5
**PC28:0**	7.8E-06	-3.5
**SMd39:1**	3.0E-06	-4.4
**PC29:0**	3.8E-06	-4.5
**LysoPC16:1**	4.2E-05	-4.6
**PE36:4**	2.7E-03	-4.7
**PC36:4**	1.8E-04	-5.8
**LysoPC14:0**	2.9E-05	-6.2
**lysoPC12:0**	1.3E-04	-6.4
**LysoPC18:2**	2.0E-05	-6.5
**PI34:2**	2.2E-05	-10.3
**SMd32:0**	2.7E-06	-10.5
**lysoPC18:3**	1.3E-05	-12.0
**PC36:5**	2.7E-06	-20.8
**SMd38:0**	6.2E-04	-23.0
**PI34:3**	3.0E-05	-31.4
**TG58:9**	3.3E-04	-38.3
**TG60:12**	1.2E-04	nd in caprine whole milk based FM

[a]Negative fold change value value means higher peak area in the soy lecithin supplemented FM samples than in the caprine whole milk based FM samples, positive fold change value means higher peak area in caprine whole milk based FM samples than in the soy lecithin supplemented FM; DG, diacylglycerol; PC, glycerophosphocholine; PC-O, ether analogue of glycerophosphocholine; PE, glycerophosphoethanoloamine; PE-O, ether anlogue of glycerophoshoethanoloamine; PS, glycerophosphoserine, PI, glycerophosphoinositol; SM, sphingomyelin; TG, triacylglycerol; TG-O, ether analogue of triacygylcerol; nd, not detected; ns – not statistically significant change

Table S11. The complete list of lipids indicating a statistically significant (p <0.01, multiple testing correction: Benjamini-Hochberg) difference between HM and FM samples in the different lactation stages and age range targets.

Lipid name	p colostrum (n=11) vs FM 0-6 mo. (n=8)	Average peak area fold change colostrum (n=11) vs FM 0-6 mo. (n=8)	p HM 0-6 mo. (n=10) vs FM 0-6 mo. (n=8)	Average peak area fold change HM 0-6 mo. (n=10) vs FM 0-6 mo. (n=8)	p HM 6- 12 mo. (n=8) vs FM 6-12 mo. (n=7)	Average peak area fold change HM 6-12 mo. (n=8) vs FM 6-12 mo. (n=7)	p HM > 12 mo. (n=16) vs FM > 12 mo. (n=5)	Average peak area fold change HM > 12 mo. (n=16) vs FM > 12 mo. (n=5)
**DG24:0**	1.2E-04	-9.8		-2.4				
**DG32:1**		1.2	1.3E-04	3.5	7.8E-05	5.0	9.5E-05	4.1
**DG34:2**	4.3E-04	-3.3		-1.1			9.4E-03	-1.8
**DG36:1**	1.2E-04	-3.8		-1.8				
**DG36:2**	1.2E-04	-5.0		-1.8			9.6E-04	-2.6
**DG36:3**	1.2E-04	-7.2		-1.6				
**DG36:4**	1.2E-04	-23.0		-2.6	5.4E-04	-2.6	3.8E-05	-4.1
**LysoPC12:0**	1.2E-04	-3.6		-3.7			1.4E-04	-3.3
**LysoPC14:0**		-1.7	1.0E-03	-3.1			8.2E-03	-2.1
**LysoPC16:0**		1.8		1.0				
**LysoPC18:0**	2.2E-04	2.9	4.0E-04	2.7	2.3E-04	4.0	2.8E-05	5.4
**LysoPC18:3**	2.5E-04	-5.2		-1.8			4.5E-03	-2.5
**LysoPC22:6**	1.2E-04	12.2	1.2E-04	15.1				
**LysoPE18:0**	1.2E-04	9.2	1.2E-04	7.1	6.5E-05	12.7	2.8E-05	26.9
**LysoPE18:2**		-1.2	2.5E-04	2.8	5.4E-04	4.2	3.1E-04	2.8
**PA46:3**	1.2E-04	-23.7	1.2E-04	nd in HM	3.4E-04	nd in HM		
**PC24:0**	1.2E-04	-16.5	1.3E-04	-16.5				
**PC26:0**	1.2E-04	-9.1	3.9E-04	-13.0	1.1E-04	-10.0	1.5E-04	-7.1
**PC28:0**	1.2E-04	-5.4	1.2E-04	-9.6	1.2E-04	-5.3	2.8E-04	-4.8
**PC29:0**	1.2E-04	-5.8	1.2E-04	-15.0	1.1E-04	-8.2	1.4E-04	-8.4
**PC30:0**		-1.4	1.2E-04	-4.8	1.9E-03	-2.6	1.8E-03	-2.4
**PC30:1**	1.3E-04	-2.3	1.3E-04	-4.3	1.4E-03	-3.0	2.0E-04	-2.5
**PC31:0**		-1.2	1.2E-04	-3.9	4.2E-04	-3.3	1.0E-04	-3.0

**PC32:0**	1.3E-04	2.3		-1.2				
**PC32:1**		-1.2	1.5E-04	-2.9	4.1E-03	-2.1		
**PC32:2**	4.4E-03	-2.7		-2.0				
**PC33:0**		-1.8	1.2E-04	-4.7	8.9E-05	-5.6	6.2E-05	-3.5
**PC33:1**	1.3E-04	-2.7	1.2E-04	-5.5	8.9E-05	-4.1	2.0E-04	-2.9
**PC33:2**	2.9E-03	-2.4		-2.1				
**PC34:0**	1.2E-04	2.9		1.4			2.2E-03	2.5
**PC34:1**		1.3		-1.4				
**PC34:3**	9.0E-04	-3.1	1.3E-03	-3.1	5.0E-04	-4.0		
**PC35:1**		1.3		-3.0				
**PC36:1**		1.4		-1.2			6.4E-03	2.0
**PC36:2**		1.5	3.6E-03	2.0			2.5E-04	3.7
**PC36:4**	1.2E-04	-11.8	8.6E-04	-6.3	1.0E-03	-6.4	3.4E-03	-3.7
**PC36:5**	1.2E-04	-36.4	1.3E-04	-13.9	1.5E-04	-16.5	9.5E-05	-15.4
**PC38:3**	1.2E-04	5.7	1.2E-04	5.3			2.0E-03	9.3
**PC38:4**							6.9E-03	2.1
**PC38:6**	1.2E-04	13.6	1.2E-04	5.1				
**PCO-34:1**	1.2E-04	5.7	9.0E-03	1.6				
**PE34:2**	2.0E-03	-4.2		-2.1				
**PE36:1**	5.9E-03	2.0	1.3E-03	2.2				
**PE36:2**		-1.4		2.4	8.7E-03	2.6		
**PE36:3**		-1.6		1.9				
**PE36:4**	1.2E-04	-15.2		-3.5	4.5E-03	-4.4		
**PE38:4**		1.6	1.6E-03	2.2				
**PE40:4**	1.2E-04	21.9	3.4E-04	10.0				
**PEO-34:2**	1.2E-04	7.6	1.2E-04	4.0	6.4E-04	3.8	3.8E-05	11.7
**PEO-36:5**	1.2E-04	33.5	1.2E-04	11.9	5.4E-04	12.0	8.8E-03	25.4
**PEO-38:5**	1.2E-04	28.5	1.2E-04	12.7				
**PEO-38:7**	1.2E-04	79.9	1.2E-04	26.6	1.9E-03	nd in FM	2.8E-05	nd in FM
**PEO-40:7**	1.2E-04	41.1	1.2E-04	18.4			2.8E-05	nd in FM
**PI34:2**	1.2E-04	-12.5	1.2E-04	-8.2			1.1E-03	-10.5

**PI34:3**	1.2E-04	-12.9	1.2E-04	nd in HM	4.5E-03	nd in HM	4.9E-06	nd in HM
**PI36:1**	5.1E-04	-2.3		1.1			1.3E-04	2.4
**PI36:2**		-1.8		1.5			1.7E-04	2.6
**PI36:4**	1.2E-04	-14.7	1.2E-04	-14.9	5.5E-03	nd in HM		
**PI38:3**	1.2E-04	5.9	1.2E-04	3.9			1.7E-03	4.4
**PI38:4**	1.2E-04	10.7	2.0E-04	3.1	2.6E-04	2.6		
**PS36:1**	1.2E-04	3.5		1.1				
**PS36:2**	1.2E-04	3.4	1.2E-04	4.8	1.7E-04	6.9	4.3E-05	8.6
**PS40:6**	1.2E-04	25.0	1.2E-04	9.7			5.8E-03	3.3
**SMd28:1**	1.2E-04	-2.0		3.0			1.8E-03	8.0
**SMd30:1**		-1.3	2.2E-04	2.3			5.1E-05	4.5
**SMd32:0**	6.7E-04	-3.5		-1.4				
**SMd32:1**							1.4E-03	2.2
**SMd32:2**	1.2E-04	5.7	1.2E-04	4.6				
**SMd33:1/SMt 32:2**		-1.4	1.2E-04	-3.6	1.2E-04	-3.0	3.8E-05	-2.4
**SMd34:0**							5.2E-04	2.0
**SMd34:1**	1.3E-04	2.2		-1.1			4.1E-03	1.9
**SMd34:2**	1.2E-04	3.3		1.1			1.5E-03	1.9
**SMd36:0**	1.2E-04	5.8	1.2E-04	18.2	1.1E-04	30.9	2.8E-05	73.5
**SMd36:1**	1.2E-04	6.0	1.2E-04	12.8	1.1E-04	14.0	2.9E-05	54.0
**SMd36:2**	1.2E-04	4.9	1.2E-04	3.6	5.4E-04	4.5	3.7E-05	8.4
**SMd38:0**	1.2E-04	-30.4		-4.0				
**SMd38:1**	1.2E-04	39.6	1.2E-04	77.1	6.5E-05	112.6	4.7E-04	227.5
**SMd39:0**	1.2E-04	-14.6	2.1E-04	-16.3				
**SMd39:1**	1.4E-03	-11.1	1.2E-04	-15.9			2.6E-03	-8.6
**SMd39:2**	1.2E-04	-15.5	1.2E-04	-17.0			6.6E-03	-4.0
**SMd40:0**	1.2E-04	-28.3	1.2E-04	-19.5	7.4E-03	-23.6	5.8E-05	nd in HM
**SMd40:1_1**	1.2E-04	2.8	1.2E-04	7.0	1.1E-04	9.1	2.9E-05	19.6
**SMd40:1_2**					5.1E-03	nd in HM		
**SMd40:2**	1.4E-03	2.2	1.2E-03	2.3			3.8E-05	5.2

**SMd41:1**	1.3E-04	-2.2		-1.4			8.8E-03	1.9
**SMd41:2**		-1.5	5.6E-04	-3.3				
**SMd42:0**		-1.0	2.6E-04	3.5	1.1E-04	7.4	2.8E-05	15.2
**SMd42:1**	1.2E-04	7.6	1.2E-04	7.4				
**SMd42:2**	1.2E-04	24.4	1.2E-04	18.8	1.1E-04	22.3	2.8E-05	45.1
**SMd42:3**	1.2E-04	108.3	1.2E-04	39.2				
**SMd43:2**		5.6	5.0E-03	-16.0				
**SMd44:1**		1.9		-1.3				
**SMd44:2**	1.2E-04	nd in FM	4.2E-04	nd in FM				
**SMt34:1**	1.2E-04	6.8		-1.6				
**SMt42:1**	1.2E-04	123.0	1.2E-04	32.7			2.8E-05	nd in FM
**TG28:0**	1.2E-04	-123.4	1.2E-04	-66.8			3.4E-03	-6.0
**TG30:0**	1.2E-04	-229.7	1.2E-04	-96.9				
**TG31:0**	1.2E-04	-31.9	1.2E-04	-19.9	3.0E-03	nd in HM		
**TG32:0**	1.2E-04	-171.1	1.2E-04	-51.7	1.1E-04	-67.4		
**TG32:1**	1.2E-04	-172.6	1.7E-04	-17.4				
**TG33:0**	1.2E-04	-72.5	1.2E-04	-29.1	2.8E-04	nd in HM		
**TG33:1**	1.6E-03	-3.5		1.0				
**TG34:0**	1.2E-04	-117.5	1.2E-04	-23.8	1.1E-04	-37.8		
**TG34:1**	1.2E-04	-33.2		-3.3				
**TG34:2**	1.2E-04	-30.6		-2.1				
**TG35:0**	1.2E-04	-62.3	1.2E-04	-19.8				
**TG36:0**	1.2E-04	-90.2	1.2E-04	-18.4	1.1E-04	-23.1		
**TG36:1**	1.2E-04	-26.0	8.2E-04	-3.5				
**TG37:0**	1.2E-04	-28.4	3.5E-04	-7.8	5.4E-04	-11.6		
**TG38:0**		-35.6		-9.2	1.1E-04	-10.3		
**TG38:1**	1.2E-04	-20.3	9.4E-04	-2.8				
**TG38:2**	1.2E-04	-13.8		-1.4				
**TG38:3**	1.2E-04	-11.6		-1.2				
**TG39:0**	1.2E-04	-18.9		-2.1				
**TG39:1**	1.2E-04	-16.6		-1.7				

**TG40:0**	1.2E-04	-8.9		-2.1	1.2E-03	-3.6	1.1E-03	8.1
**TG40:1**		-4.5	2.1E-04	1.6				
**TG40:2**	1.2E-04	-7.8		1.1				
**TG40:3**		-3.3		2.9				
**TG41:0**	5.6E-04	-4.6		1.0				
**TG41:1**	6.5E-04	-4.0		1.7				
**TG42:0**	1.2E-04	-5.7		-1.8			6.4E-03	8.1
**TG42:1**	4.8E-04	-2.7		2.0	9.6E-03	1.7	5.1E-05	17.2
**TG42:2**	6.2E-04	-3.5		2.5			7.6E-05	24.8
**TG42:3**		-3.3		3.6				
**TG43:0**		-1.3	9.3E-03	2.6			9.4E-03	7.3
**TG43:1**		-1.5	6.1E-03	4.2				
**TG44:0**	5.8E-03	-1.8		1.3			4.7E-05	10.3
**TG44:1**		-1.1	1.3E-04	3.4	6.7E-03	3.0	3.7E-05	21.1
**TG44:2**		1.2	1.2E-04	6.8	5.2E-03	4.0	3.7E-05	48.8
**TG44:3**	1.4E-04	-2.8	4.0E-03	3.2			3.9E-04	17.7
**TG46:1**	1.2E-04	2.7	1.2E-04	5.8	6.5E-05	5.3	2.8E-05	21.3
**TG46:2**		1.9	1.2E-04	6.8	2.6E-04	5.3	2.8E-05	44.1
**TG46:3**		1.9	1.2E-04	13.1	7.6E-04	7.6	3.2E-05	33.8
**TG46:4**	2.7E-04	-1.6	1.2E-04	9.1				
**TG47:3**	1.2E-04	4.0	1.2E-04	19.7				
**TG48:1**	1.2E-04	2.3	1.2E-04	3.2	2.3E-04	3.4	2.9E-05	6.9
**TG48:2**	1.2E-04	2.7	1.2E-04	5.8	6.5E-05	6.7	2.8E-05	11.8
**TG48:3**	1.2E-04	4.9	1.2E-04	19.0	6.5E-05	14.8	2.8E-05	64.2
**TG48:4**	1.2E-04	4.8	1.2E-04	25.9	6.5E-05	16.6	2.8E-05	69.5
**TG48:5**	1.2E-04	4.9	1.2E-04	41.8	4.5E-03	31.0	8.7E-04	51.3
**TG50:2**		1.7	2.4E-04	2.4	5.9E-03	2.4	1.0E-04	2.8
**TG50:3**	1.2E-04	4.7	1.2E-04	9.2	6.5E-05	7.9	2.8E-05	13.4
**TG50:4**	1.2E-04	3.6	1.2E-04	9.5	6.5E-05	7.1	2.8E-05	14.0
**TG50:5**	1.2E-03	4.0	1.2E-04	15.3	1.1E-04	12.8	1.8E-04	20.4
**TG50:6**	1.2E-04	10.5	1.2E-04	22.7			8.8E-03	54.9

**TG51:3**	2.1E-04	2.2		1.0				
**TG51:4**		-16.0		1.6				
**TG52:1**							1.7E-03	2.3
**TG52:2**							8.7E-04	1.6
**TG52:3**		1.3	2.9E-04	2.2			4.3E-04	2.1
**TG52:4**		-1.6		1.4				
**TG52:5**		-1.7		1.5				
**TG52:6**	1.2E-04	-5.3		-1.8				
**TG52:7**	1.2E-04	7.8	1.2E-04	22.7				
**TG53:1**		1.3	7.6E-04	2.0			1.1E-03	3.6
**TG53:2**	4.7E-03	1.8	1.7E-04	2.3	1.4E-03	3.1	2.0E-04	3.1
**TG53:3**		1.6	1.2E-04	3.4	2.6E-04	2.9	7.6E-05	3.2
**TG54:1**	2.1E-04	-1.8	9.7E-04	-1.7				
**TG54:2**	3.5E-04	-1.8		-1.5				
**TG54:3**	1.2E-04	-3.7	1.2E-04	-2.5			3.9E-04	-2.3
**TG54:4**	1.2E-04	-2.5		-1.3			3.1E-03	-1.7
**TG54:5**	1.2E-04	-6.1	2.8E-04	-2.3	1.0E-03	-3.2	7.6E-05	-3.1
**TG54:5_2**	1.2E-04	3.0	1.2E-04	2.7	1.1E-04	2.1	3.1E-05	2.7
**TG54:6**	1.2E-04	-19.7	1.2E-04	-5.2	1.1E-04	-7.8	2.8E-05	-6.7
**TG54:7**	1.2E-04	-34.2	1.2E-04	-6.3	4.2E-04	-8.5	3.2E-05	-10.3
**TG54:7_2**	1.2E-04	4.7	1.2E-04	7.1			2.4E-03	9.0
**TG54:8**	1.2E-04	-58.3	2.7E-04	-6.8				
**TG55:2**							5.7E-04	2.5
**TG56:2**		-1.4	2.2E-04	-2.0				
**TG56:3**		-1.2	3.3E-03	-1.8			7.1E-04	-1.9
**TG56:4**							1.8E-03	-1.6
**TG56:6_1**	1.2E-04	53.4	1.2E-04	43.8				
**TG56:6_2**	1.2E-04	1.7	1.2E-04	1.9				
**TG56:7_1**	1.2E-04	7.7	1.2E-04	21.9				
**TG56:7_2**	1.2E-04	13.2	1.2E-04	9.1	9.6E-03	7.2	3.2E-05	27.2
**TG56:8**	1.2E-04	11.8	1.2E-04	12.9				

**TG56:9**	1.2E-04	4.0	1.2E-04	6.3			8.0E-03	10.7
**TG57:2**	1.4E-04	-3.5	2.5E-04	-3.2				
**TG58:10**	1.2E-04	1.9	1.2E-04	4.4				
**TG58:11**		-13.5		-11.9				
**TG58:2**	1.0E-03	-2.4	1.2E-04	-11.5	2.1E-04	-7.8	1.8E-04	-6.7
**TG58:3**		1.0	4.9E-04	-2.3				
**TG58:4**		-1.4	1.2E-04	-3.8	8.7E-04	-3.7	1.4E-03	-2.8
**TG58:5**		2.8		1.1				
**TG58:6**	1.2E-04	74.1	1.2E-04	31.6	4.5E-03	17.1	2.8E-05	20.3
**TG58:7_1**	1.2E-04	38.7	1.2E-04	31.0				
**TG58:7_2**	1.2E-04	19.4	1.2E-04	15.0	9.6E-03	9.9	9.4E-03	31.1
**TG58:8_1**	1.2E-04	45.4	1.2E-04	45.3				
**TG58:8_2**	1.2E-04	12.5	1.2E-04	15.5				
**TG58:9**	1.2E-04	3.0	1.2E-04	4.3				
**TG60:12**		nd in HM		nd in HM	2.0E-03	nd in HM	3.4E-03	-10.8
**TG60:2**	1.9E-04	-3.2	1.2E-04	-17.6	2.3E-04	-8.6	3.7E-05	-13.8
**TG60:3**	1.2E-04	-262.7	1.2E-04	-6.4				
**TG60:4**		-1.9	1.2E-04	-7.1	1.1E-04	-10.4	4.7E-04	-7.7
**TG60:5**	9.2E-03	2.7		-2.2				
**TG62:3**	1.3E-04	6.8	1.3E-04	-3.7				
**TG62:4**	1.2E-04	4.2	4.2E-04	-4.0			2.6E-03	-9.0
**TG62:5**	2.0E-03	2.2	1.4E-04	-6.4			6.4E-03	-16.2
**TG62:6**	1.2E-04	5.1	1.6E-04	-1.7	9.3E-03	nd in HM		
**TG62:7**	1.2E-04	3.7		-2.4				
**TG62:8**	1.2E-04	-1.4	1.2E-04	-36.2				
**TG63:2**	1.2E-04	-6.6	1.2E-04	-22.9				
**TG63:6**	5.8E-04	-1.2	1.2E-04	-2.9				
**TG64:4**	1.2E-04	6.1		-2.9				
**TG64:6**	1.2E-04	14.9		1.2				

**TG64:8**		-1.4	1.2E-04	nd in HM	7.9E-04	nd in HM		
**TG66:18**	1.2E-04	-16.0	1.2E-04	-16.0				
**TGO-50:1**	1.2E-04	109.5	1.2E-04	23.5			1.0E-04	nd in FM
**TGO-52:1**	1.2E-04	16.9	1.2E-04	3.4	7.8E-03	9.0	1.0E-04	nd in FM
**TGO-52:2**	1.2E-04	96.0	1.2E-04	28.6				
**TGO-54:2**	1.2E-04	20.7	1.2E-04	7.6			4.3E-05	nd in FM
**TGO-54:3**	1.2E-04	66.5	1.2E-04	28.2			6.4E-04	nd in FM

nd, not detected; ns, not statistically significant; blank space correspond to the not statistically significant different lipids; [a] negative fold change value value means higher peak area in the FM samples than in the HM samples, positive fold change value means higher peak area in the HM samples than in the FM samples. DG, diacylglycerol PC, glycerophosphocholine; PC-O, ether analogue of glycerophosphocholine; PE, glycerophosphoethanoloamine; PE-O, ether anlogue of glycerophoshoethanoloamine; PS, glycerophosphoserine, PI, glycerophosphoinositol; SM, sphingomyelin; TG, triacylglycerol; TGO, ether analogue of triacyglycerol.

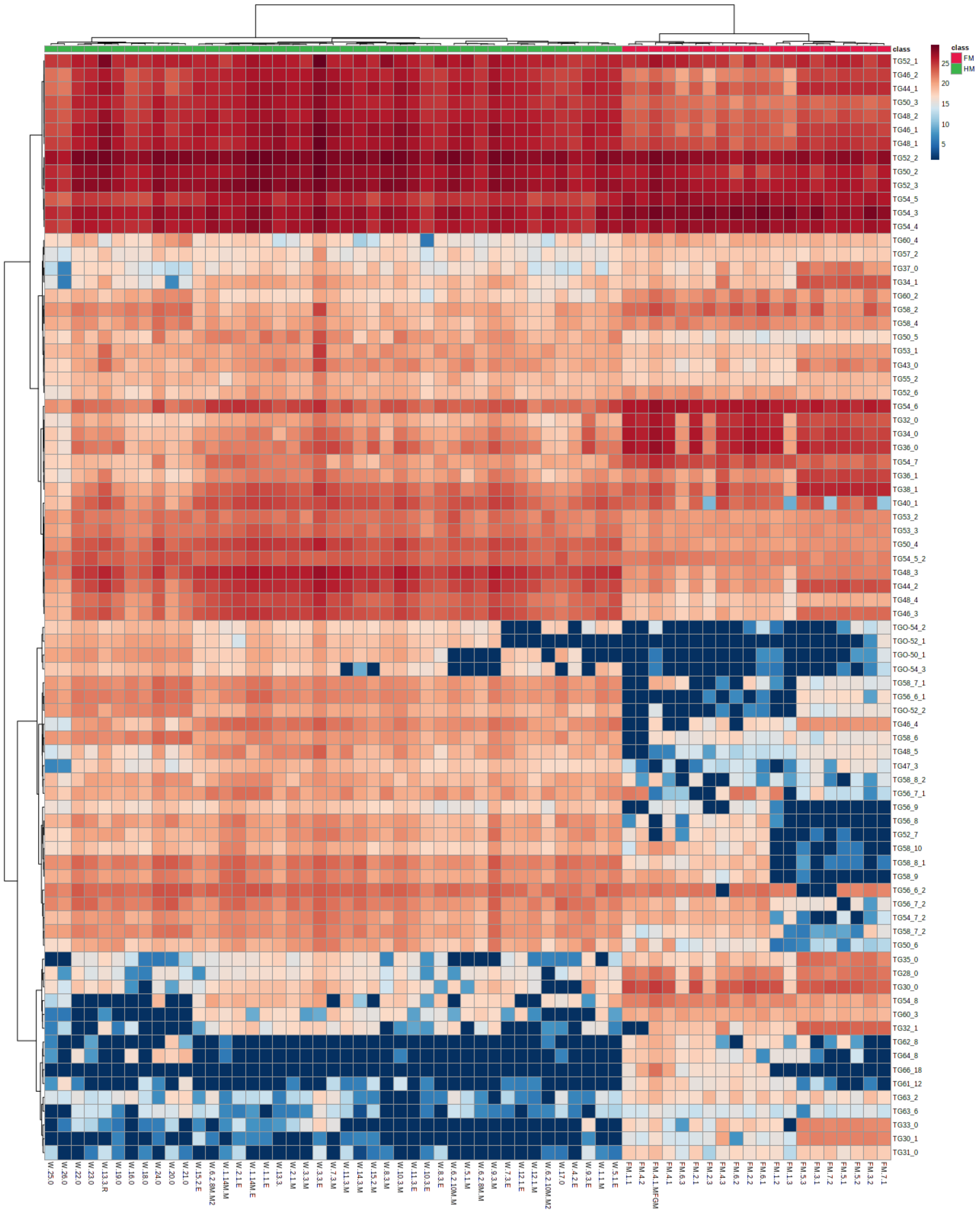

Figure S3. Clustering shown as a heatmap (distance measured using the Euclidean algorithm, and the clustering algorithm using Ward's method, for the top 80 TGs indicated in the Mann-Whitney test unpaired.

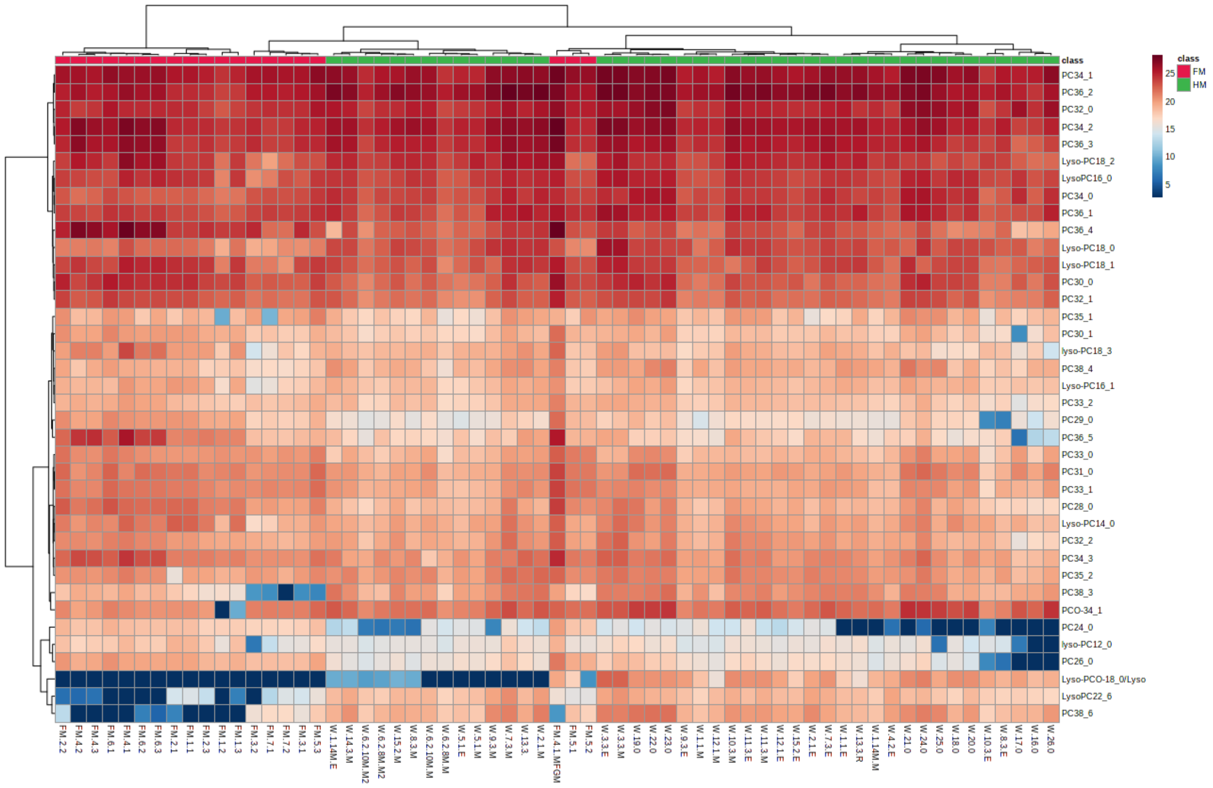

Figure S4. Clustering shown as a heatmap (distance measured using the Euclidean algorithm, and the clustering algorithm using Ward's method for the PCs detected in HM and/or FM samples.

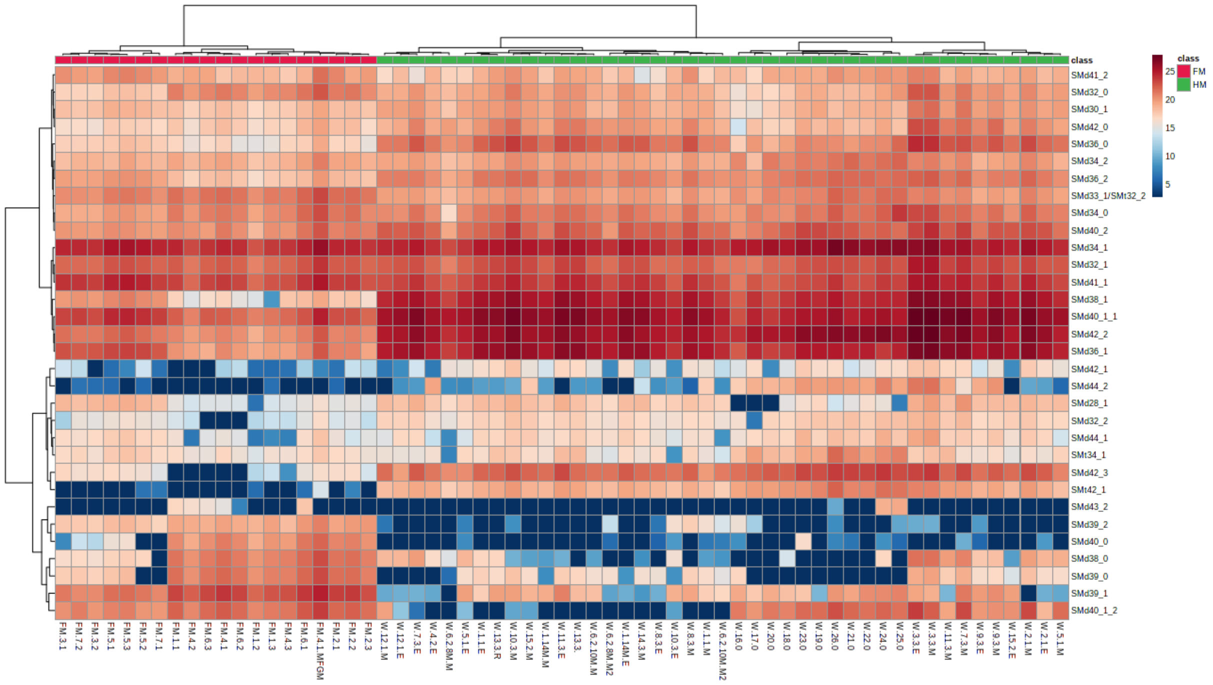

Figure S5. Clustering shown as a heatmap (distance measured using the Euclidean algorithm, and the clustering algorithm using Ward's method for the SMs detected in HM and/or FM samples.

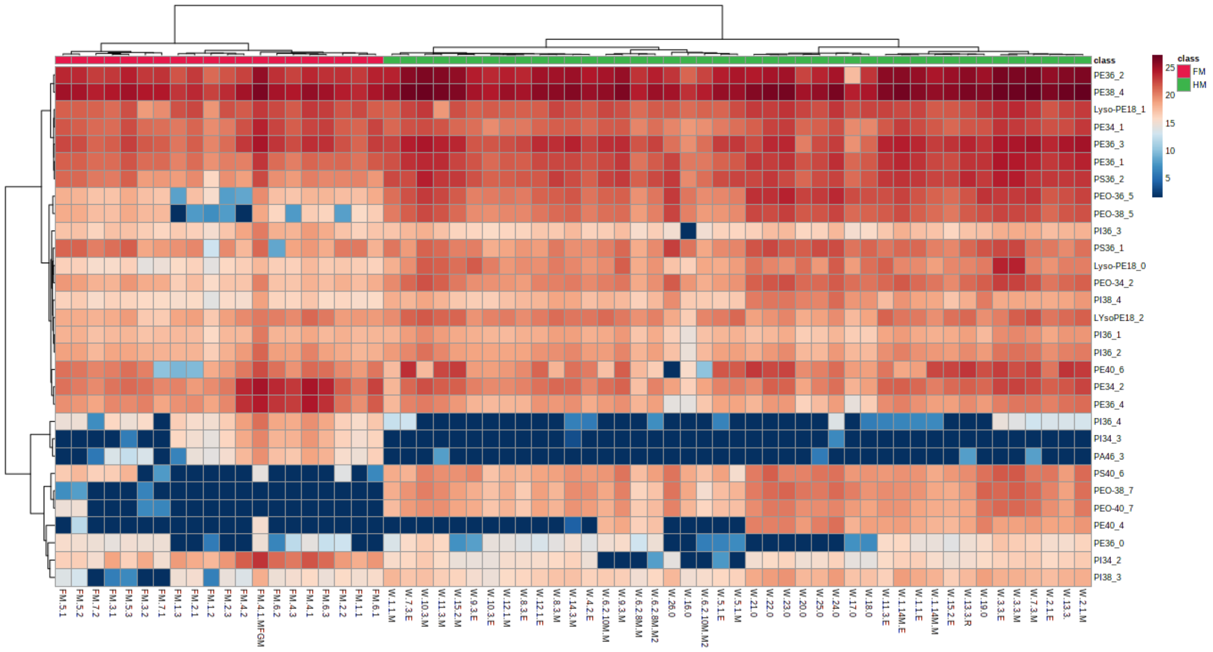

Figure S6. Clustering shown as a heatmap (distance measured using the Euclidean algorithm, and the clustering algorithm using Ward's method for the PEs, PIs and PSs detected in HM and/or FM samples.

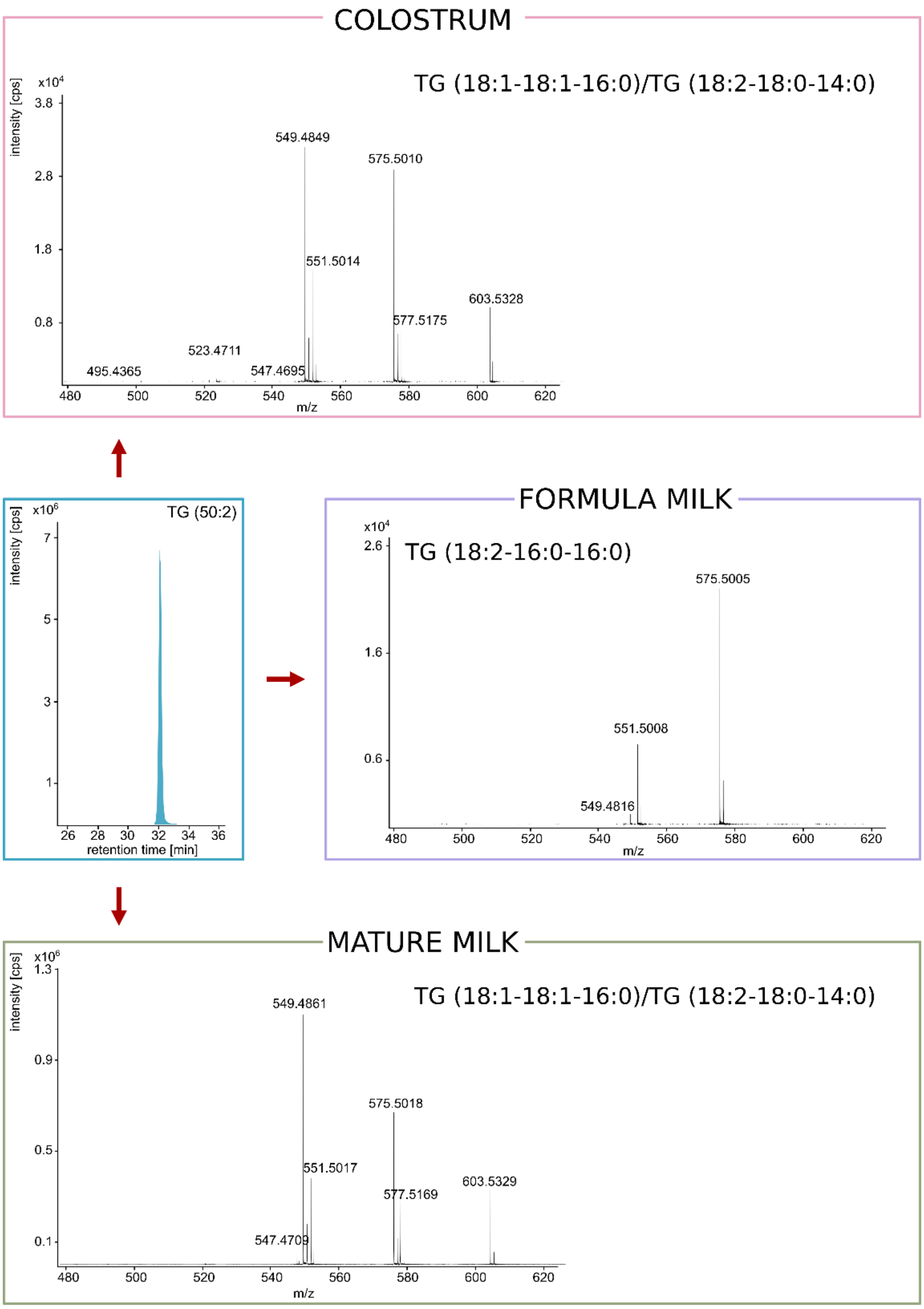

Figure S7. The MS/MS spectra and fatty acyl substituents distribution of the TG50:2 detected in HM and FM samples.TG, triacylglycerol.
